# The Diterpenes Ovoideal A–G from *Tirpitzia ovoidea*

**DOI:** 10.3390/molecules191118966

**Published:** 2014-11-18

**Authors:** Dan Su, Xue-Yan Yang, Xu Feng, Ming-Ying Shang, Shao-Qing Cai

**Affiliations:** Department of Natural Medicines, School of Pharmaceutical Sciences, Peking University Health Science Center, Xueyuan Road, Beijing 100191, China; E-Mails: sudan2000@163.com (D.S.); bucm2009@163.com (X.-Y.Y.); xufeng_pharm@163.com (X.F.); sqcai@bjmu.edu.cn (S.-Q.C.)

**Keywords:** *Tirpitzia ovoidea*, Linaceae, diterpene, cytotoxic activity

## Abstract

Seven new diterpenes, named ovoideal A (**1**), B (**2**), C (**3**), D (**4**), E (**5**), F (**6**) and G (**7**), have been isolated along with eleven known diterpenes **8**–**18** from the petroleum ether soluble fraction of an ethanol extract of the aerial parts of *Tirpitzia ovoidea*. The structures of the new compounds were elucidated primarily by 1D and 2D NMR spectroscopy, as well as by the HR-ESI-MS spectrometry. All compounds were isolated from the Linaceae family for the first time. The *in vitro* cytotoxic activity of compounds **1**, **3**–**5**, **8**–**18** was evaluated against the Hela, HepG2 and K562 cell lines. Among them, compounds **3**, **9**, **11**, **12**, **13**, **14**, **15**, **17**, **18** showed moderate inhibitory activities.

## 1. Introduction

**Figure 1:**
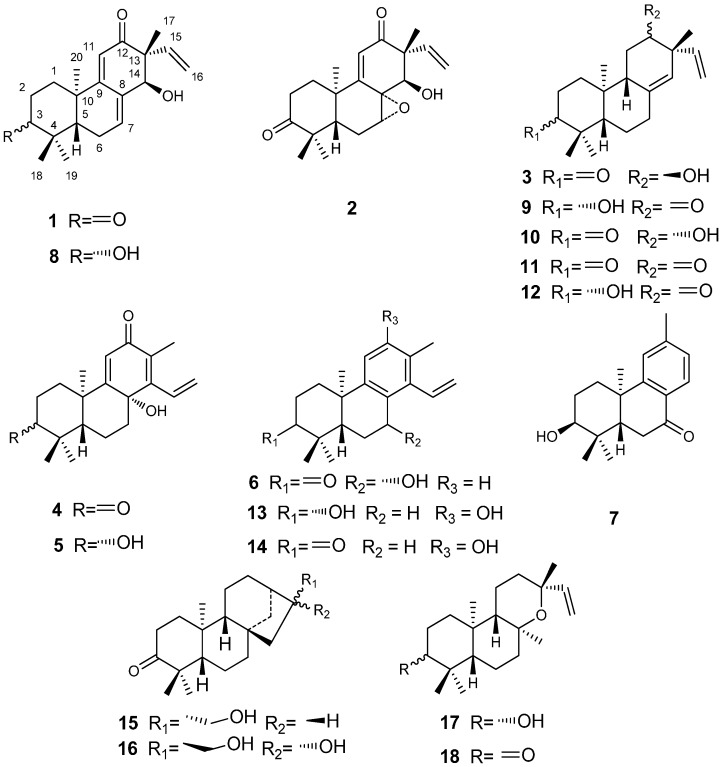
Chemical structures of compounds **1**–**18**.

**Figure 2:**
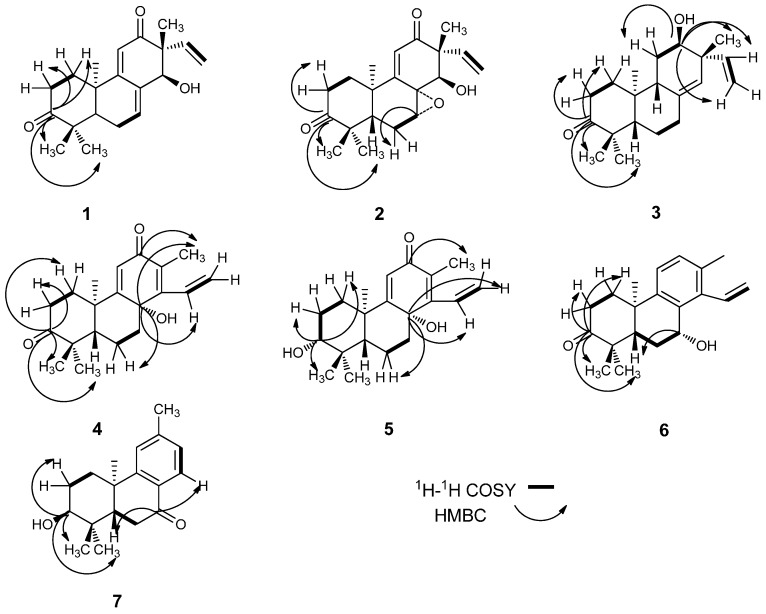
Main ^1^H-^1^H COSY and HMBC correlations of compounds **1**–**7**.

*Tirpitzia ovoidea* Chun et How ex Sha (Linaceae family) is distributed in Chongzuo, Jingxi, Longzhou, Mashan in Guangxi of China, while Vietnam has some distribution too [1]. The dried aerial parts, containing stems, branches and leaves of this species, is known in China as Baihuachai and has been used as a folk medicine in Guangxi. Baihuachai was used to promoting blood circulation for removing blood stasis, relieving muscle rigidity and activating collaterals and in the treatment of rheumatism, traumatic hemorrhage, injuries from falls, fractures, contusions and strains [1]. The Mulao people use it to treat chronic hepatitis, traumatic infections, fractures, hepatalgia and anepithymia caused by liver cancer [2]. As a folk medicine it has precise therapeutic effects, while there has been no research on its chemical constituents and pharmacological activities. In particular, diterpenoids are thought by many to be the largest pool of chemically diverse and physiologically interesting metabolites [3,4], displaying properties such as anti-leishmania activity [5], cytotocity [6], anti-microbial activity [7], anti-HIV activity [8] and neuro-protection activity [9]. In our research eighteen diterpenes, including seven new compounds (ovoideal A, B, C, D, E, F and G, Figure 1 and Figure 2) were isolated from the petroleum ether soluble fraction of an ethanol extract of the stems, branches and leaves of *Tirpitzia ovoidea* and their structures were elucidated. All of them were isolated from Linaceae family for the first time. Fourteen of the isolated compounds were evaluated for their cytotoxic activity.

## 2. Results and Discussion

### 2.1. Chemistry

The petroleum ether soluble fraction of the ethanol extract of the aerial parts of *Tirpitzia ovoidea* was subjected to repeated column chromatography to afford seven new diterpenes, ovoideals A–G (compounds **1**–**7**), and 11 known compounds. The structures of the known compounds were determined by comparing their spectroscopic data with literature values [10,11,12,13,14,15,16,17], and they were thus identified as *ent*-3β,14α-hydroxypimara-7,9(11),15-triene-12-one (**8**), *ent*-3β-hydroxypimara-8(14),15-diene-12-one (**9**), *ent*-12β-hydroxypimara-8(14),15-diene-3-one (**10**), *ent*-pimara-8(14),15-diene-3,12-dione (**11**), *ent*-3β,12α-dihydroxypimara-8(14),15-diene (**12**), spruceanol (**13**), sonderianol (**14**), *ent*-17-hydroxy-16α-kaur-3-one (**15**), abbeokutone (**16**), *ent*-3β-hydroxy-13-*epi*-manoyl oxide (**17**), and *ent*-13-*epi*-manoyl oxide-3-one (**18**). Compounds **13** and **14** were evaluated for their cytotoxic activities before [6], and they showed strong inhibition against the Hela, HT29, MCF-7, MM96L and K562 cell lines (their ED_50_ values were between 2.8 to 18 μg/mL). Compound **17** showed strong inhibition against the Raji, MOLT3 and H9 cell lines [18].

Compound **1** (Figure 1) was obtained as colorless crystals and its molecular formula was determined to be C_20_H_26_O_3_ by HRESIMS, which displayed a quasi-molecular ion peak at *m*/*z* 315.1985 ([M+H]^+^).The ^1^H-NMR spectrum (Table 1), and HSQC spectroscopic data analyses of **1** indicated the presence of an ABX coupling system at δ 5.81 (1H, dd, *J* = 17.5, 10.9 Hz, H-15), 5.20 (1H, dd, *J* = 17.5, 0.8 Hz, H-16a) and 5.14 (1H, dd, *J* = 10.9, 0.8 Hz, H-16b). It displayed resonances for the C-15, C-16 vinyl moiety [19,20,21], which is characteristic of pimarane diterpenoids [11], indicating that compound **1** was likely to be of this skeletal class. The ^1^H-NMR spectrum also indicated the presence of oxygenated methine protons at δ 4.29 (s, H-14) and four tertiary methyl protons at δ 1.12 (3H, s, CH_3_-17), δ 1.20 (3H, s, CH_3_-18), δ 1.09 (3H, s, CH_3_-19) and δ 1.32 (3H, s, CH_3_-20). The ^13^C-NMR (Table 2) and HSQC spectrum showed 20 carbons, including four methyl, four methylene (one olefinic), five methine (three olefinic, one oxygenated), and seven quaternary carbons (two olefinic, two carbonyl).

**Figure 3:**
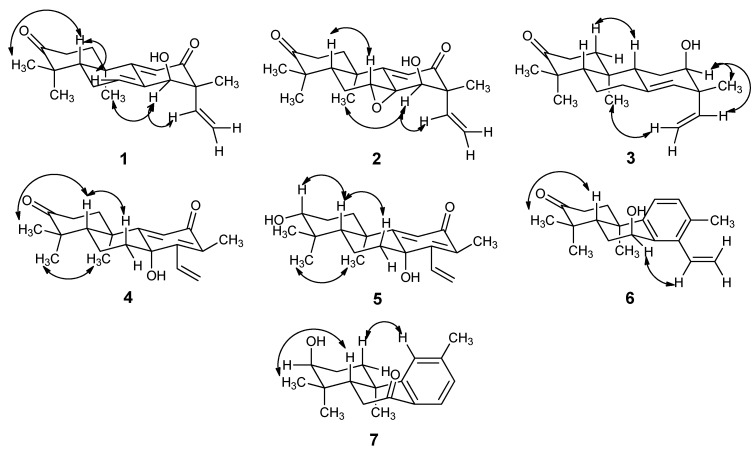
Main NOESY correlations of compounds **1**–**7**.

The ^1^H-^1^H COSY spectrum (Figure 2), in combination with HSQC data, showed cross-peaks at δ 1.84/2.95 (H-1a/H-2b), 2.44/5.75 (H-6/H-7), 5.14/5.81, 5.20/5.81 (H-15/H-16), thus confirming the following structural fragments: -CH_2_CH_2_- (C1-C2), -CH_2_CH- (C6-C7), and -CH=CH_2_- (C15-C16). When the spectroscopic data of **1** were compared with *ent*-3β,14α-hydroxypimara-7,9(11),15-triene-12-one [10], the resonances of a hydroxyl group were replaced with a carbonyl group. The most deshielded signal at δ*_C_* 216.9 belonged to a carbonyl group had the long-range HMBC (Figure 2) couplings of H-2 (216.9/2.95, 216.9/2.32), H-1 (216.9/2.27, 216.9/1.84), H-18 (216.9/1.09) and H-19 (216.9/1.20), indicating that this carbonyl position was assigned at C-3. Cross-peaks in the NOESY (Figure 3) spectrum between H-14 and H-20, H-14 and H-15 at δ 4.29/1.32, 4.29/5.81, revealed that H-14, C_20_-CH_3_, and the vinyl group are on the same face of the molecule. This allowed assignment of relative stereochemistry of compound **1**. Thus the structure of compound **1** was identified as *ent*-14α-hydroxypimara-7,9(11),15-triene-3,12-dione and was named ovoideal A.

Compound **2** (Figure 1) was obtained as colorless crystals and its molecular formula was determined to be C_20_H_26_O_4_ by HRESIMS, which displayed a quasi-molecular ion peak at *m*/*z* 329.1755 ([M−H]^−^). Compared the ^1^H-NMR and ^13^C-NMR (Table 1 and Table 2) spectrum of **2** with **1**, we concluded that **2** was shown to be 7,8-epoxide of *ent*-14-hydroxy-pimara-9(11), 15-diene-3,12-dione. The configuration of the epoxy ring was assigned as α in the **2**. The assignment due to H-7 in **2** had the 2D NOESY (Figure 3) correlation with H-5. Furthermore, the skeleton of *ent*-pimarane had decided the stereochemistry of C-8. Meanwhile, the configuration of the hydroxyl group of C-14 was determined as α-oriented by virtue of the correlation of H-14 and H-15 in NOESY spectrum. We suggest the structure of compound **2** is *ent*-14α-hydroxy-7α,8α-epoxypimara-9(11),15-diene-3,12-dione, named ovoideal B.

Compound **3** (Figure 1) was obtained as a light yellow gum and its molecular formula was determined to be C_20_H_30_O_2_ by HRESIMS, which displayed a quasi-molecular ion peak at *m*/*z* 301.2190 ([M−H]^−^). Comparing the ^1^H-NMR and the ^13^C-NMR spectra (Table 1 and Table 2) of **3** with those of the known compound **11**, we could note that their A and B rings were the same and they just differed in C ring. It was clear that the carbonyl group at C ring in **3** was reduced into a hydroxyl group. The signal at δ*_C_* 72.4 belonged to the carbon connected with the hydroxyl group which had long-range HMBC couplings (Figure 2) of H-11 (72.4/1.65), H-14 (72.4/5.09), H-15 (72.4/5.71), H_2_-16 (72.4/4.98, 4.99) and H-17 (72.4/1.04), indicating that this hydroxyl group was located at C-12. The stereochemistry was determined by the analysis of 2D NOESY (Figure 3) spectrum, which showed correlations between H-12 and H-17, H-12 and H-15, H-16 and H-20, indicating that the hydroxyl group was α-oriented. Consequently we suggest the structure of compound **3** is *ent*-12α-hydroxypimara-8(14),15-diene-3-one, named ovoideal C.

Compound **4** (Figure 1) was obtained as colorless crystals and its molecular formula was determined to be C_20_H_26_O_3_ by HRESIMS, which displayed a quasi-molecular ion peak at *m*/*z* 315.1959 ([M+H]^+^). ^1^H-NMR spectrum (Table 1) showed signals at δ 1.94 (3H, s) for an olefinic methyl group, δ 1.09 (3H, s), 1.16 (3H, s), 1.57 (3H, s) for three tertiary methyl protons, δ 6.06 (1H, s) for an olefinic proton. It also gave signals for an ABX system at δ 6.64 (1H, dd, *J* = 17.9, 11.8 Hz, H-15), 5.57 (1H, dd, *J* = 17.9, 1.76 Hz, H-16a) and 5.67 (1H, dd, *J* = 11.9, 1.8 Hz, H-16b), corresponding to a monosubstituted vinyl group attached to an olefinic carbon nucleus. The combination of these groups in a single nucleus suggested this compound was a member of the cleistanthane series [22]. The ^13^C-NMR spectrum (Table 2) revealed all 20 carbons, which in combination with HSQC measurements were found to be four methyl, five methylene (one olefinic), three methine (two olefinic), and eight quaternary carbons (three olefinic, three oxygenated). ^1^H-^1^H COSY (Figure 2) spectrum, in combination with HSQC data, showed cross-peaks at δ 1.97/2.47, 1.97/2.87 (H-1/H-2), 1.52/1.62 (H-5/ H-6), 1.27/1.62, 1.27/2.13 (H-6/H-7), 6.64/5.56, 6.64/5.67 (H-15/H-16), confirming the following structural fragments: -CH_2_CH_2_- (C1-C2), -CHCH_2_CH- (C5-C6-C7), and -CH=CH_2_- (C15-C16). The most deshielded carbonyl carbon at δ*_C_* 218.0, has the long-range HMBC correlations with H-1 (2.07, 1.97), H-2 (2.87, 2.47), H-18 (1.09) and H-19 (1.16), thus confirmed the location of this carbonyl group was at C-3. Also, another carbonyl group was located at C-12 from the cross-peak at 189.0/1.94 with H-17. The carbon at δ*_C_* 71.5, which was connected with a hydroxyl group, was assigned to C-8 because of the cross-peaks with H-6, H-7, H-11, H-15 and H-17. The coupling constant values of H-5 was 2.6 and 12.6, demonstrating that H-5 was axial-oriented [23]. Also cross-peaks in the NOESY (Figure 3) spectrum at 1.16/1.57 (H-19/H-20) revealed that C-20 methyl group was axial-oriented. Therefore we propose that the 8β-hydroxy-cleistanth-9(11),13,15-triene-3,12-dione structure for compound **4**, which was named ovoideal D.

**Table 1:** ^1^H-NMR Data of Compounds **1**−**7**.

POSITION	δ_H_ Mult (*J*)						
	1 *^a^*	2 *^b^*	3 *^b^*	4 *^a^*	5 *^b^*	6 *^b^*	7 *^b^*
1a	1.84 m	1.83 td	1.43 m	1.97 m	1.58 m	2.02 m	2.09 m
1b	2.27 td	2.25 m	1.89 m	2.07 td	1.73 m	2.47 m	2.09 m
2a	2.32 m	2.42 ddd	2.25 dt	2.47 ddd	1.78 m	2.66 dd (6.4, 8.8)	1.84 m
2b	2.95 m	2.74 td	2.58 td	2.87 ddd	1.78 m	2.66 dd (6.4, 8.8)	2.16 m
3					3.21 dd (5.1, 10.9)		3.57 brs
4							
5	1.88 t (5.4)	2. 02 m	1.49 m	1.52 dd (2.6, 12.6)	0.94 dd (2.2, 12.6)	2.43 dd (4.8, 10.8)	2.34 dd (5.0, 13.1)
6a	2.44 m	2.02 m	1.56 m	1.62 m	1.66 m	1.92 m	2.63 m
6b	2.44 m	2.25 m	1.56 m	2.13 m	1.96 ddd	1.94 m	2.63 m
7a	5.75 s	3.80 d (1.6)	2.09 m	1.27 m	1.25 td	5.01 t	
7b			2.36 dd (14.4, 2.7)	2.39 m	2.37 dt		
8							
9			1.89 m				
10							
11	6.51 brs	6.20 s	1.65 m	6.06 s	6.03 s	7.14 d (8.0)	7.18 s
12			3.63 brs			7.17 d (8.0)	
13							7.10 d (7.9)
14	4.29 s	4.13 brs	5.09 s				7.90 d (7.9)
15	5.81 dd (10.9 17.5)	5.95 dd (10.8, 17.2)	5.71 dd (10.1, 17.7)	6.64 dd (17.9, 11.8)	6.56 dd (11.8, 17.9)	6.96 dd (11.2, 17.6)	
16a	5.20 dd (17.5, 0.8)	5.31 brd (17.2)	4.98 d (10.1)	5.56 dd (17.9, 1.76)	5.52 dd (1.8, 17.9)	5.38 dd (2.0, 17.6)	
16b	5.14 dd (10.9, 0.8)	5.41 brd (10.8)	4.99 d (17.7)	5.67 dd (11.9, 1.8)	5.64 dd (1.8, 11.8)	5.67 dd (2.0, 11.6)	
17	1.12 s	1.29 s	1.04 s	1.94 s	1.91 s	2.29 s	2.39 s
18	1.09 s	1.12 s	1.05 s	1.09 s	0.99 s	1.21 s	1.03 s
19	1.20 s	1.15 s	1.02 s	1.16 s	0.91 s	1.13 s	1.02 s
20	1.32 s	1.31 s	0.91 s	1.57 s	1.39 s	1.22 s	1.25 s

*^a^* In MeOD: δ (ppm), *J* (Hz) 400 MHz for ^1^H-NMR; *^b^* In CDCl_3_: δ (ppm), *J* (Hz) 400 MHz for ^1^H-NMR.

**Table 2:** ^13^C-NMR Data of Compounds **1**−**7**.

POSITION	δ_C_						
	1 *^a^*	2 *^b^*	3 *^b^*	4 *^a^*	5 *^b^*	6 *^b^*	7 *^b^*
1	36.5	35.1	37.1	37.5	36.1	37.5	30.5
2	35.3	34.0	34.5	35.3	27.3	34.6	25.5
3	216.9	213.8	216.5	218.0	78.3	217.3	75.2
4	48.8	46.8	37.9	49.1	39.7	37.6	37.7
5	50.7	40.3	55.2	55.9	54.2	43.2	42.7
6	25.5	22.4	23.1	19.7	17.5	28.6	35.6
7	118.1	53.1	35.0	40.5	39.7	64.9	199.1
8	134.5	55.5	137	71.5	70.9	138.5	144.8
9	164.7	162.5	45.6	171.1	169.0	144.9	155.9
10	38.6	37.5	47.7	42.0	41.3	46.7	37.6
11	134.4	127.8	26.0	122.2	121.3	124.7	128.6
12	204.5	201.1	72.4	189.3	187.5	130.6	124.1
13	56.3	55.2	43.6	130.1	129.6	133.8	127.1
14	75.8	69.5	125.4	159.1	155.8	133.9	127.5
15	140.6	138.6	146.0	134.0	132.3	135.1	
16	116.7	117.3	114.0	123.6	123.2	121.2	
17	15.9	16.3	23.3	12.5	12.2	20.6	22.1
18	25.1	24.9	25.6	26.4	28.3	27.0	27.5
19	22.7	22.8	22.1	22.3	15.5	21.0	21.7
20	20.1	21.8	14.3	20.2	20.7	24.0	23.3

*^a^* In MeOD: δ (ppm) 100 MHz for ^13^C-NMR; *^b^* In CDCl_3_: δ (ppm) 100 MHz for ^13^C-NMR.

Compound **5** (Figure 1) was obtained as a white amorphous powder and its molecular formula was determined to be C_20_H_28_O_3_ by HRESIMS, which displayed a quasi-molecular ion peak at *m*/*z* 317.2115 ([M+H]^+^). Comparing the ^1^H-NMR (Table 1) and ^13^C-NMR (Table 2) spectra of **5** with those of compound **4**, we found that **5** just differed from **4** in the presence of a hydroxyl group instead of a carbonyl group. The carbon connected with the hydroxyl group at δ_C_ 78.3, has the long-range HMBC (Figure 2) correlations with H-1, H-2, H-18 and H-19, indicated the location of this hydroxyl group at C-3. In NOESY spectrum there were correlations between H-3 and H-5, so we suggest the structure of compound **5** to be 3β,8β-dihydroxycleistanth-9(11),13,15-triene-12-one, named ovoideal E.

Compound **6** (Figure 1) was obtained as colorless crystals and its molecular formula was determined to be C_20_H_26_O_2_ by HRESIMS, which displayed a quasi-molecular ion peak at *m*/*z* 297.1862 ([M−H]^−^). Comparing the ^1^H-NMR (Table 1) and ^13^C-NMR (Table 2) spectra of **6** with those of the known compound **14**, we noted that **6** differed from **14** only in the location of the hydroxyl group, as the carbon connected with the hydroxyl group at δ*_C_* 64.9, has long-range HMBC (Figure 2) correlations with H-5 and H-6; also the proton of this carbon correlated with C-8 and C-9, so we propose the *peri* location of this hydroxyl group at C-7. In the NOESY spectrum, there were correlations between H-7 and H-15, suggesting that the hydroxyl group was β-oriented. Considering the stereochemistry of compound **14**, we propose that the structure of compound **6** is 7β-hydroxycleistanth-8,11,13,15-tetraene-3-one, named ovoideal F.

Compound **7** (Figure 1) was obtained as colorless crystals and its molecular formula was determined to be C_18_H_24_O_2_ by HRESIMS, which displayed a quasi-molecular ion peak at *m*/*z* 273.1855 ([M+H]^+^). The molecular formula indicated the presence of seven double-bond equivalents in the molecule. The ^13^C-NMR spectrum (Table 2) further showed that **7** had one ketonic carbonyl carbon conjugated with the aromatic ring (δ 199.1), three tertiary and three quaternary olefinic (of the aromatic ring), four methyl, three methylene, two methane, and two saturated quaternary carbons. The ^1^H-NMR spectrum (Table 1) showed four singlets of three protons each at δ 1.02 (H-17), 1.03 (H-16), 1.25 (H-18) and 2.39 (H-15). One one-proton singlet, two one-proton doublets were observed at 7.18 (s), 7.10 (dd, *J* = 7.9) and 7.90 (dd, *J* = 7.9), attributed to protons at C-11, C-13 and C-14, respectively. These downfield splitting signals for H-11, H-13 and H-14 and their coupling constant values showed that there existed a 1,2,4-trisubstituted benzene ring in the molecule. These data, along with the seven double-bond equivalents, indicated that **7** was a diterpenoid with a tricyclicpodocarpane type of skeleton [24]. The ^1^H-^1^H COSY (Figure 2) spectrum, in combination with HSQC data, showed cross-peaks at δ 1.84/2.09 (H-1/H-2), 1.84/3.57 (H-2/H-3), 2.34/2.63 (H-5/H-6), 7.10/7.90 (H-13/H-14), thus confirming the following structural fragments: -CH_2_CH_2 _CH- (C1-C2-C3), -CHCH_2_- (C5-C6), and ‑CH=CH- (C13-C14), so the aromatic methyl substituent was at C-12. The assignment of the location of the carbonyl group was at C-7 by virtue of the HMBC correlations with C-7 and H-14. We also observed cross-peaks at 75.2/1.84, 75.2/1.02, confirming that the hydroxyl group was located at C-3. The H-3 peak was a broad singlet, revealing that the hydroxyl group was axial. On the basis of the general stereochemistry of the compounds isolated from this plant, we propose that the absolute configuration of **7** is 3*S*,5*S*,10*R*, so we suggest the structure of compound **7** to be 3α-hydroxy-12-methyl-podocarpa-8,11,13-triene-7-one, named ovoideal G.

### 2.2. Cytotoxity Activity

Many diterpenes have strong anti-tumor activities on human cancer cell lines [25,26], so the *in vitro* cytotoxity activities for all these compounds, except for **2**, **6** and **7**, against Hela, HepG2 and K562 were evaluated in our study. 

**Table 3:** IC_50_ values (μM) of compound **3**, **9**, **11**–**15**, **17**–**18** for human cell lines.

Compound *^a^*	IC_50_ (μM)		
	Hela	HepG2	K562
**3**	92.3	80.2	>100
**9**	85.0	>100	>100
**11**	84.2	>100	86.4
**12**	83.0	54.7	66.3
**13**	87.4	59.0	91.0
**14**	89.5	>100	45.1
**15**	92.1	66.2	58.6
**17**	91.4	>100	>100
**18**	95.0	>100	>100
Taxol *^b^*	<0.1	<0.1	8.18
5-Fu *^b^*	64.12	33.69	82.0

*^a^* Compounds not listed in the table were inactive (IC_50_ > 100 μM) against all cell lines; *^b^* Taxol and 5-Fu were used as positive controls.

Clonogenic-type assays for comparing the growth of treated cells showed some inhibitory activity for compounds **3**, **9**, **11**–**15**, **17**, **18** in a range of tumor cell lines (Table 3). The anti-cancer activity of compounds **3**, **11**, **12**, **15** and **18** are reported for the first time in this study.

## 3. Experimental Section 

### 3.1. General

Optical rotations were recorded on a JASCO DIP-140 digital polarimeter (Tokyo, Japan). IR spectra (KBr) were obtained on a Nicolet Nexus 470 FTIR Spectrometer (Madison, WI, USA). NMR data were acquired using Bruker AVANCE DRX 400 spectrometer (Fällanden, Switzerland) at 400 MHz (^1^H) and 100 MHz (^13^C), with tetramethylsilane (TMS) as an internal standard, and chemical shifts were indicated in δ values (ppm). HR-ESI-MS spectra were measured on a Waters Xevo G2 Q-TOF mass analyzer (Milford, MA, USA). Column chromatography (CC) was performed with silica gel (100–200 and 200–300 mesh, Qingdao Marine Chemical Factory, Qingdao, China) and Sephadex LH-20 (GE Healthcare Bio-Science AB, Uppsala, Sweden). TLC was conducted on silica gel GF254 plates (10–40 μm; Qingdao Marine Chemical, Inc., Qingdao, China). Spots were observed by UV light as well as by spraying with 5% anisaldehyde-H_2_SO_4_-EtOH followed by heating. Semipreparative HPLC was performed using a Thermo Scientific ODS Hypersil column (250 mm × 21.2 mm i.d.) on a Shimadzu HPLC system (Tokyo, Japan) consisting of a LC-20AD pump and a SPD-M20A UV/vis detector. Tumor cells (Hela: CCL-2^TM^, HepG2: CCL-185^TM^ and K562: HB-8065^TM^) were purchased from American Type Culture Collection (ATCC, Rockville, MD, USA), were incubated in a NuAir CO_2_ incubator (Nuair, Tampa, FL, USA) and observed in a CKX41 inverted microscope (Motic, Xiamen, China). Optical density (OD) values were read under a Model 680 microplate reader (BIO-RAD, Hercules, CA, USA). 5-Fluorouracil (5-FU) (128K1409) and taxol (BCBB4101) were purchased from Sigma Inc. (Saint Louis, MO, USA).

### 3.2. Plant Material

The aerial parts of *Tirpitzia ovoidea* were collected from Wuming, Guangxi, China in April 2012 and identified by Associate Professor Ming-Ying Shang. A voucher specimen (No. 7349) was deposited in the Herbarium of Pharmacognosy, School of Pharmaceutical Sciences, Peking University Health Science Center.

### 3.3. Extraction and Isolation

The air-dried and powdered aerial parts of *Tirpitzia ovoidea* (29 kg) were sequentially extracted with refluxing 95% ethanol (290 L) and 50% ethanol (232 L) for 2 h each. The extract was combined and concentrated under reduced pressure to obtain a crude extract (1,695 g). After suspension in water, the crude extract was extracted with petroleum ether, EtOAc and *n*-BuOH, successively. The extracts were concentrated under reduced pressure at 40 °C yielding petroleum ether (200 g), ethyl acetate (100 g) and *n*-butanol (563 g) fractions, respectively. The petroleum ether soluble fraction (190 g) was subjected to silica gel (200–300 mesh) column chromatography eluting with PE-EtOAc (17:8), to generate fractions V1-V6. Fraction V2 (88.1 g) was subjected to column chromatography over polyamide, eluted with H_2_O-MeOH (9:1→0:1), to give sub-fractions V2-1 to V2-5. Sub-fraction V-2-2 was subjected to silica gel column chromatography and semipreparative HPLC to afford compound **18** (16.42 mg) and compound **11** (43.22 mg). 

V3 (7.6 g) was chromatographed on Sephadex LH-20 eluting with *n*-hexane-CH_2_Cl_2_-MeOH (4:5:1), to yield V3-1 to V3-9. Sub-fraction V3-6 was subjected to repeated silica gel column chromatography and then semipreparative HPLC to yield compound **17** (55.50 mg). Sub-fraction V3-7 was subjected to vacuum silica gel column chromatography, eluted with *n*-hexane-EtOAc (19:1→0:1) to generate sub-fractions V3-7-1 to V3-7-8. Sub-fractions V3-7-5 and V3-7-7 were subjected to preparative thin layer plate chromatography with *n*-hexane-EtOAc (7:3) as the developing solvent, and then purified by semipreparative HPLC to afford compounds **7** (3.55 mg), **6** (1.02 mg), **3** (61.98 mg) and **10** (3.96 mg). Sub-fraction V3-7-6 was purified over SephadexLH-20, eluting with MeOH to yield compound **9** (68.41 mg). Sub-fraction V3-9 was subjected to vacuum silica gel column chromatography eluting with *n*-hexane-EtOAc (17:3) to give V3-9-2 and V3-9-5, which were purified by semipreparative HPLC to get compounds **14** (61.29 mg) and **4** (7.08 mg).

V5 was subjected to medium-pressure silica gel column chromatography, eluting with PE-EtOAc (78:22→55:45) to afford V5-1 to V5-9. Sub-fraction V5-5 was subjected to Sephadex LH-20 chromatography eluting with *n*-hexane-CH_2_Cl_2_-MeOH (4:5:1) to generate sub-fractions V5-5-1 to V5-5-9. Sub-fraction V5-5-8 was subjected to silica gel column chromatography eluting with *n*-hexane-EtOAc (gradient from 80%), then purified over Sephadex LH-20, eluting with MeOH to yield compound **13** (25.47 mg). Sub-fraction V5-7 was subjected to silica gel column chromatography eluting with *n*-hexane-EtOAc (gradient 3:1→1:1) to afford V5-7-1 to V5-7-5. Sub-fraction V5-7-3 was applied to a vacuum silica gel column, and purified by semipreparative HPLC to afford compound **1** (45.70 mg). 

Fraction V4 was subjected to column chromatography over polyamide, eluted with H_2_O-MeOH (9:1→0:1), to give sub-fractions V4-1 to V4-7. Sub-fractions V4-2, V4-3, V4-4 and V4-5 were chromatographed on a vacuum silica gel column with *n*-hexane/EtOAc (1:0→95:5), then purified by semipreparative HPLC to afford compounds **2** (2.74 mg), **5** (7.10 mg), **15** (29.59 mg) and **8** (54.30 mg).

V6 was subjected to polyamide column chromatography, eluted with H_2_O/MeOH (9:1→0:1), to give sub-fractions V6-1 to V6-6. Sub-fraction V6-2 was subjected to medium-pressure silica gel column chromatography, eluting with *n*-hexane-EtOAc (9:1→1:1), and then purified by semipreparative HPLC to yield compound **16** (14.16 mg). V6-3 was chromatographed on a vacuum silica gel column with *n*-hexane/EtOAc (4:1→3:2), then purified by semipreparative HPLC to afford compound **12** (17.12 mg).

### 3.4. Compound Characterization

*ent-14α-Hydroxypimara-7,9(11),15-triene-3,12-dione* (ovoideal A, **1**): Colorless crystals (MeOH); ${\left[\text{α}\right]}_{\text{D}}^{23}$ +8.6 (*c* 0.007, MeOH); UV, λ_max_(MeOH) 290 nm; IR, ν_max_ 3462, 2973, 2935, 2870, 1709, 1664, 1643, 1583, 1453, 1431, 1386, 1365, 1269, 1221, 1111, 1082, 1011, 988, 918 cm^−1^; ^1^H-NMR and ^13^C-NMR data, see Table 1; HRESIMS positive mode *m*/*z* [M+H]^+^ 315.1985 (calcd 315.1960).

*ent-14α-Hydroxy-7α,8α-epoxypimara-9(11),15-diene-3,12-dione* (ovoideal B, **2**): Colorless crystals (MeOH); UV, λ_max_ (MeOH) 238 nm; ^1^H-NMR and ^13^C-NMR data, see Table 1; IR, ν_max_ 3367, 2971, 2936, 1705, 1667, 1605, 1558, 1455, 1387, 1268, 1079, 1048, 921, 880, 827 cm^−1^; HRESIMS negative mode *m*/*z* [M−H]^−^ 329.1755 (calcd 329.1753).

*ent-12α-Hydroxypimara-8(14),15-diene-3-one* (ovoideal C, **3**): Light yellow gum (MeOH); UV, λ_max_ (MeOH) 190 nm; IR, ν_max_ 3461, 3080, 2941, 2869, 2107, 1704, 1633, 1535, 1456, 1432, 1385, 1367, 1313, 1196, 1143, 1106, 1054, 997, 973, 917, 891, 846, 731 cm^−1^; ^1^H-NMR and ^13^C-NMR data, see Table 1; HRESIMS negative mode *m*/*z* [M−H]^−^ 301.2190 (calcd 301.2168).

*8β-Hydroxycleistanth-9(11),13,15-triene-3,12-dione* (ovoideal D, **4**): Colorless crystals (MeOH); [α]^23^_D_ −10.0 (*c* 0.001, MeOH); UV, λ_max_ (MeOH) 200 nm; IR, ν_max_ 3441, 2961, 2922, 2866, 1706, 1649, 1619, 1459, 1432, 1376, 1190, 1040, 1012, 957, 934, 888 cm^−1^; ^1^H-NMR and ^13^C-NMR data, see Table 1; HRESIMS positive mode *m*/*z* [M+H]^+^ 315.1959 (calcd 315.1960).

*3β,8β-Dihydroxycleistanth-9(11),13,15-triene-12-one* (ovoideal E, **5**): Colorless gel (MeOH); UV, λ_max_ (MeOH) 207 nm; IR, ν_max_ 3429, 2943, 2868, 1650, 1616, 1456, 1362, 1191, 1095, 1043, 1003, 929, 888 cm^−1^; ^1^H-NMR and ^13^C-NMR data, see Table 1; HRESIMS positive mode *m*/*z* [M+H]^+^ 317.2115 (calcd 317.2117).

*7β-Hydroxycleistanth-8,11,13,15-tetraene-3-one* (ovoideal F, **6**): Colorless crystals (MeOH); UV, λ_max_ (MeOH) 220 nm; IR, ν_max_ 3369, 2969, 2929, 1703, 1671, 1605, 1553, 1455, 1383, 1263, 1076, 1047, 920, 880, 825 cm^−1^; ^1^H-NMR and ^13^C-NMR data, see Table 1; HRESIMS negative mode *m*/*z* [M−H]^−^ 297.1862 (calcd 297.1855).

*3α-Hydroxy-12-methyl-podocarpa-8,11,13-triene-7-one* (ovoideal G, **7**): Light yellow crystals (MeOH); UV, λ_max_ (MeOH) 260 nm; IR, ν_max_ 3423, 2963, 2874, 1672, 1604, 1559, 1456, 1411, 1388, 1295, 1267, 1210, 1186, 1137, 1051, 992, 943, 881, 825, 690, 642, 590, 545, 507 cm^−1^; ^1^H-NMR and ^13^C-NMR data, see Table 1; HRESIMS positive *m*/*z* [M+H]^+^ 273.1855 (calcd 273.1855).

### 3.5. Cytotoxicity Activity 

The cytotoxic effects of compounds were estimated against the Hela, HepG2 and K562 cancer cell lines by using the MTT assay method. The cell suspensions were distributed into 96-well cell culture plates and cultured at 37 °C under a 5% CO_2_ atmosphere in an incubator for 24 h. The compounds were dissolved with limited DMSO and diluted to five different concentrations (0.1, 1, 10, 100, 200 µM) with RPMI 1640 before adding to the corresponding well. After 48 h cultivation, MTT was added to each well for 4 h cultivation. Finally, the supernatant was discarded and limited DMSO was added to the well to dissolve the blue-violet crystal, then the optical density (OD) values were read on the microplate reader at 492 nm. All tests and analyses were carried out in triplicate. DMSO, 5-FU and taxol were applied as the blank control and positive control, respectively.

## 4. Conclusions

Seven new diterpenes: *ent*-14α-hydroxypimara-7,9(11),15-triene-3,12-dione, named ovoideal A (**1**), *ent*-14α-hydroxy-7α,8α-epoxypimara-9(11),15-diene-3,12-dione, named ovoideal B (**2**), *ent*-12α-hydroxypimara-8(14),15-diene-3-one, named ovoideal C (**3**), 8β-hydroxycleistanth-9(11),13,15-triene-3,12-dione, named ovoideal D (**4**), 3β,8β-dihydroxycleistanth-9(11),13,15-triene-12-one, named ovoideal E (**5**), 7β-hydroxycleistanth-8,11,13,15-tetraene-3-one, named ovoideal F (**6**) and 3α-hydroxy-12-methylpodocarpa-8,11,13-triene-7-one, named ovoideal G (**7**), were isolated from the petroleum ether soluble fraction of ethanol extract of the aerial parts of *Tirpitzia ovoidea* along with the eleven known diterpenes **8**–**18**. The cytotoxity of these compounds was tested against the Hela, HepG2 and K562 cancer cell lines using the MTT assay method, which indicated some compounds showed moderate inhibitory activities.

## Supplementary Materials

Supplementary materials can be accessed at: http://www.mdpi.com/1420-3049/19/11/18966/s1.

## Supplementary Files

Supplementary File 1
