# Supplementary material for: The Diterpenes Ovoideal A–G from Tirpitzia ovoidea
Source: Molecules. 2014 Nov 18;19(11):18966–79. doi: 10.3390/molecules191118966 (PMC6270903; doi:10.3390/molecules191118966)

Supplementary

Figure S1. UV spectrum of compound **1** in CH_3OH.

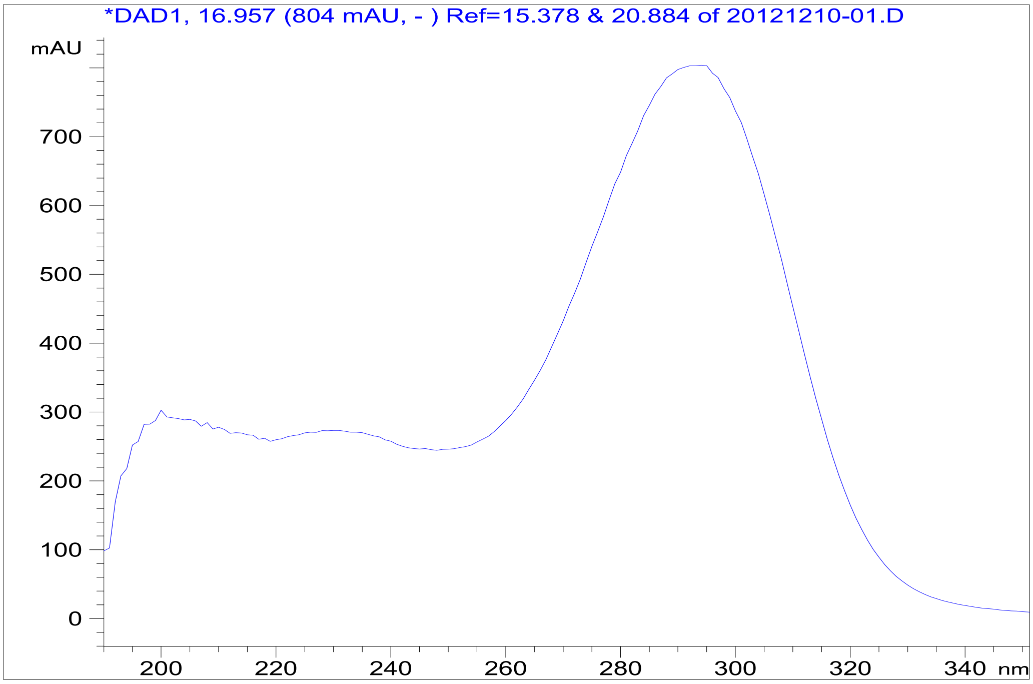

Figure S2. IR spectrum of compound **1**.

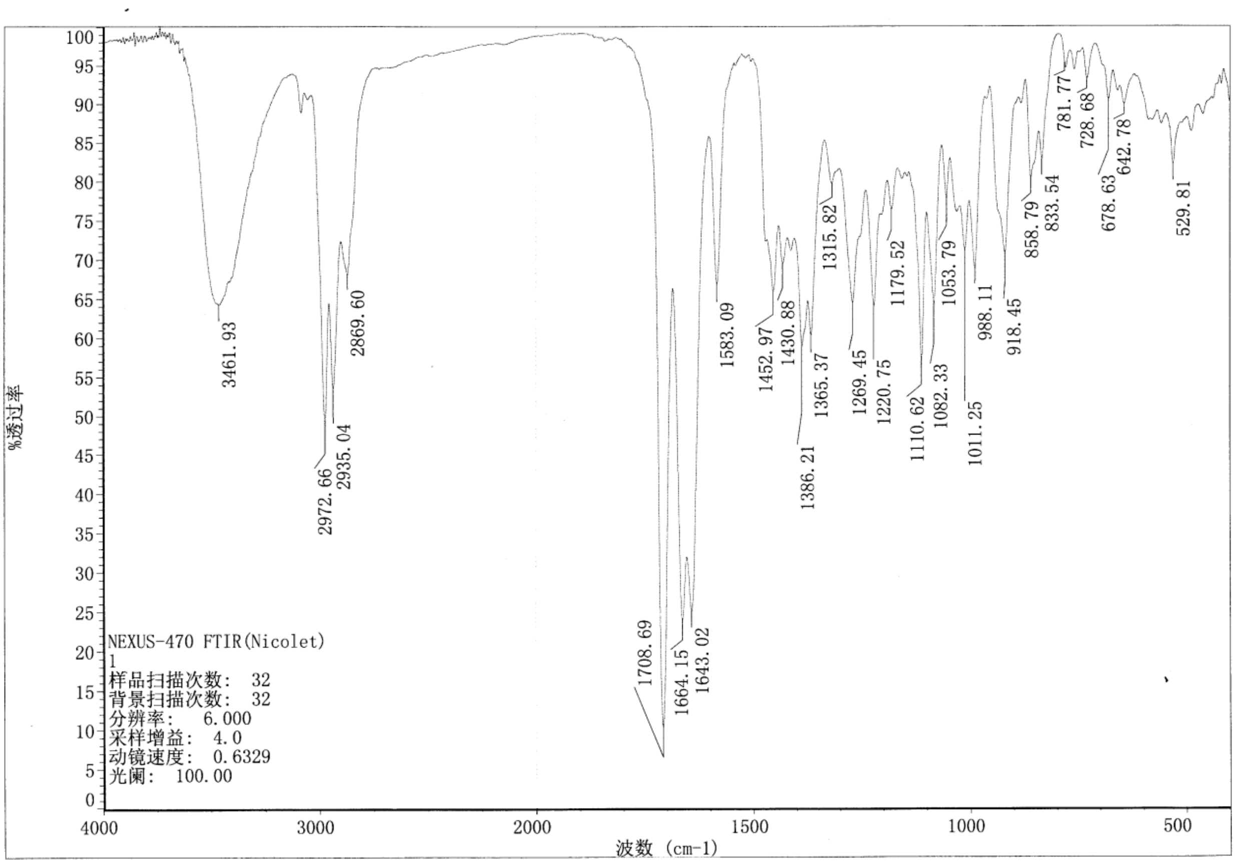

Figure S3. HR-ESI-TOF-MS spectrum of compound **1**.

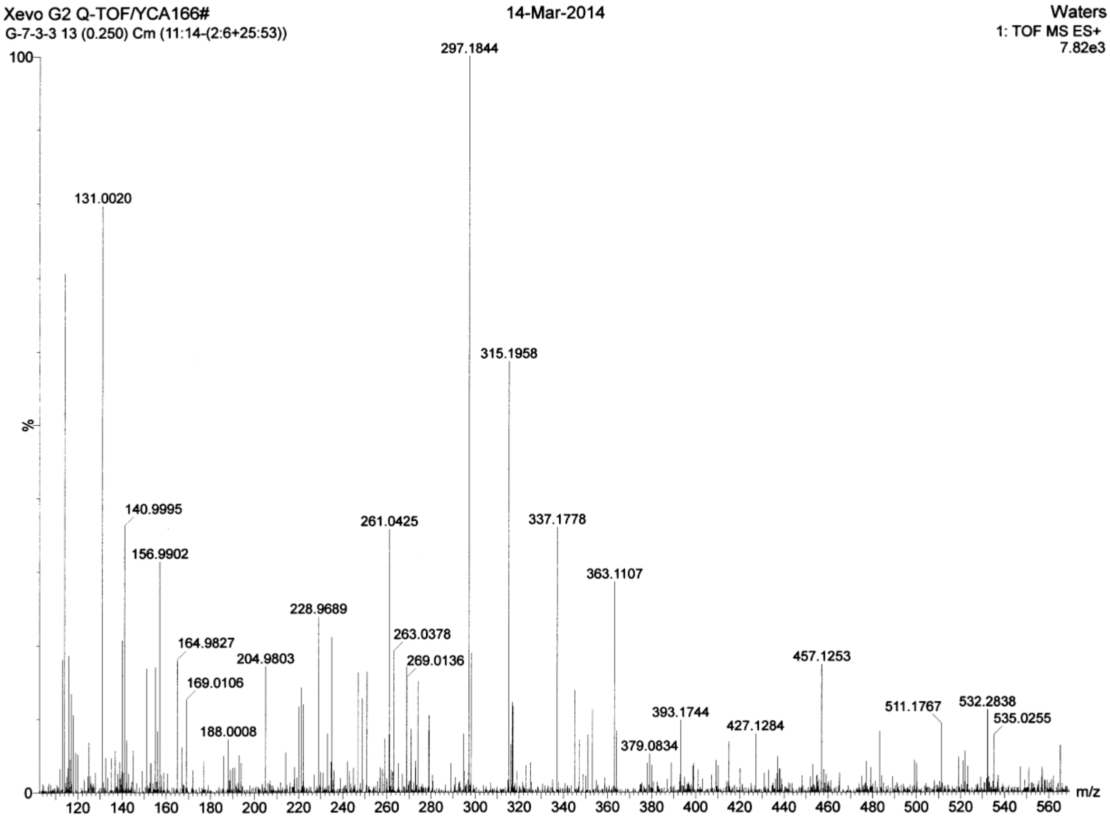

Figure S4. ^{1}H-NMR spectrum of compound **1** in CD_3OD.

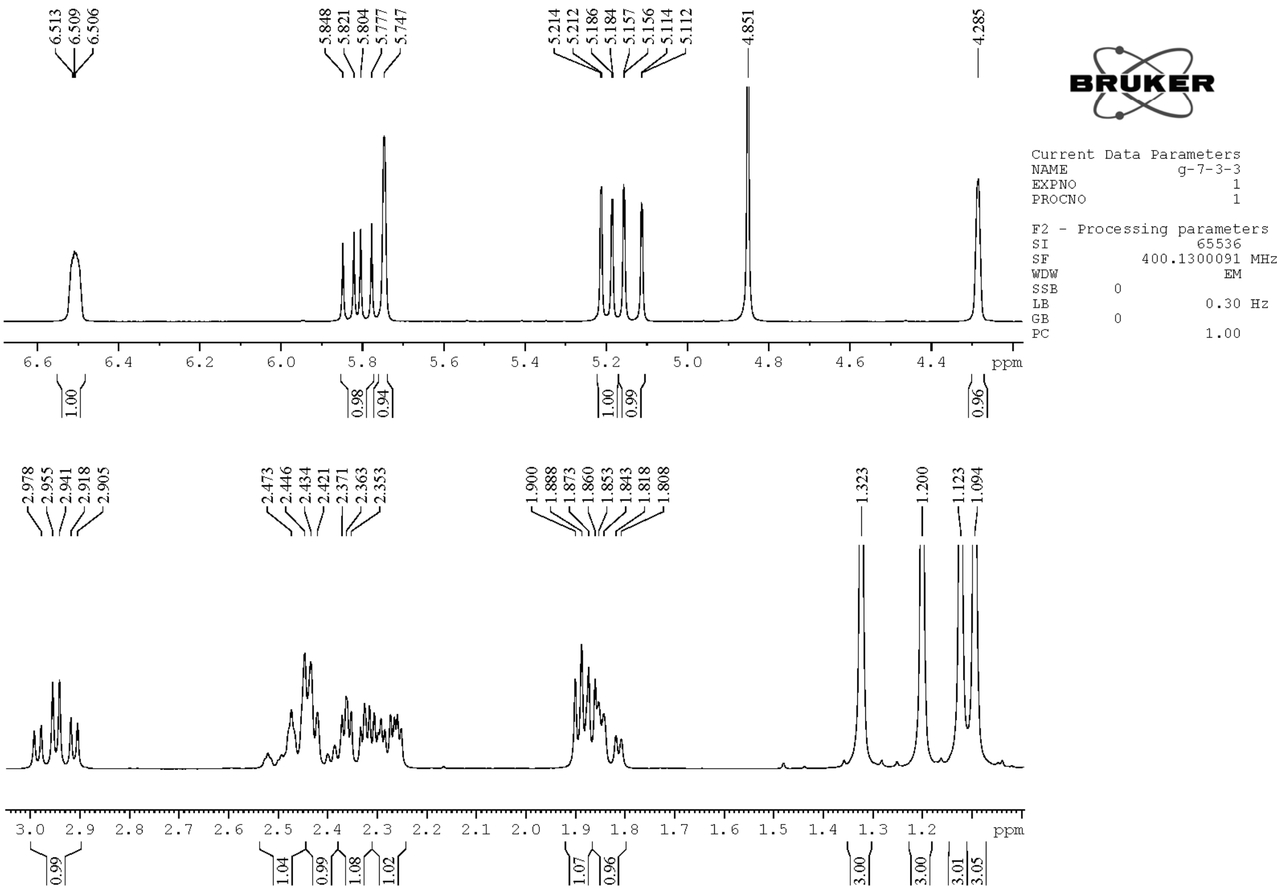

Figure S5. ^{13}C-NMR spectrum of compound **1** in CD_3OD.

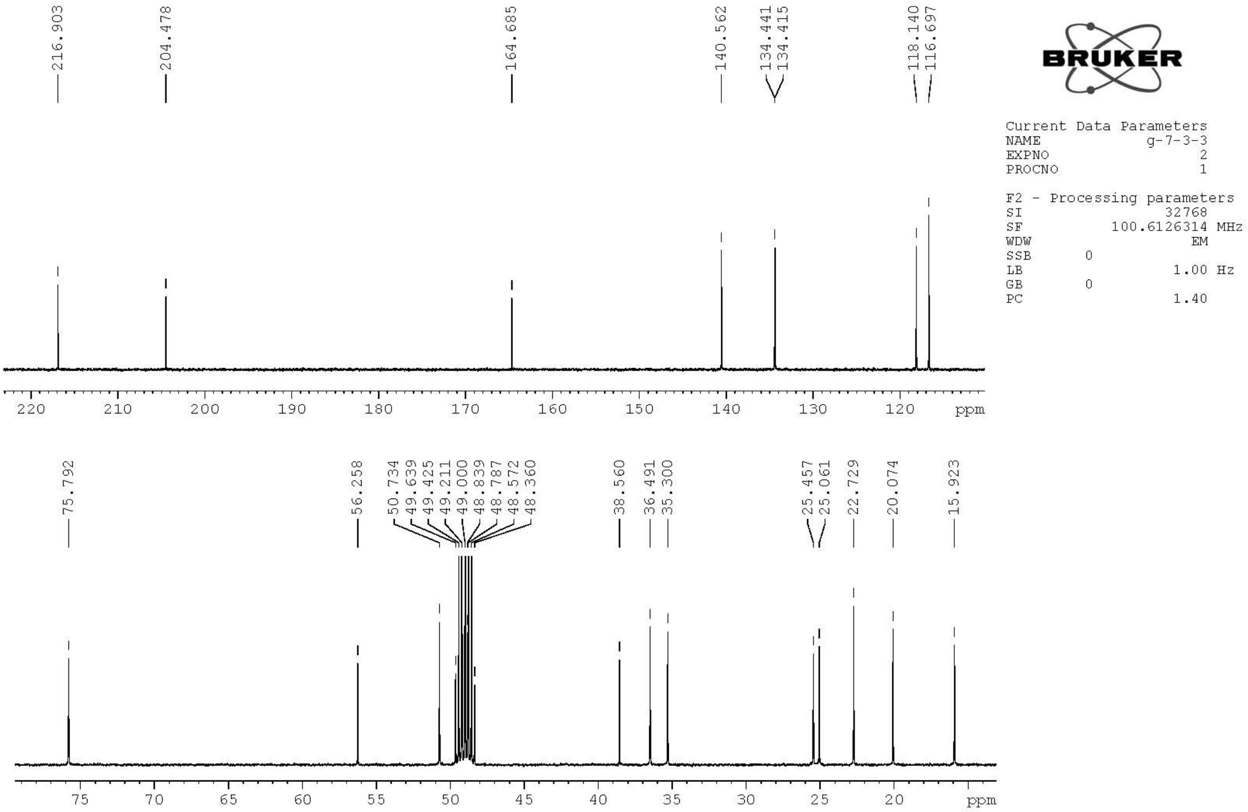

Figure S6. ^{1}H^{1}H COSY spectrum of compound **1** in CD_3OD.

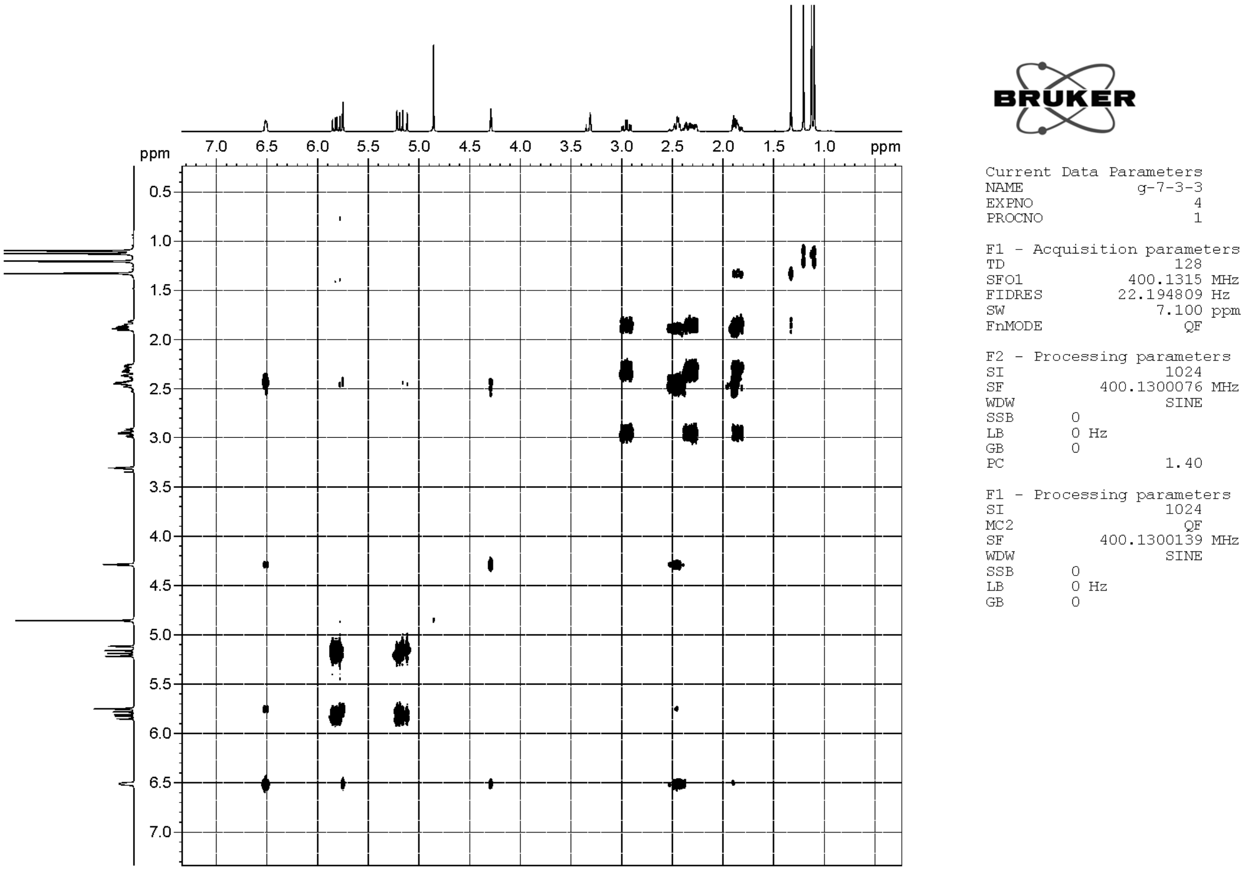

Figure S7. HSQC spectrum of compound **1** in CD_3OD.

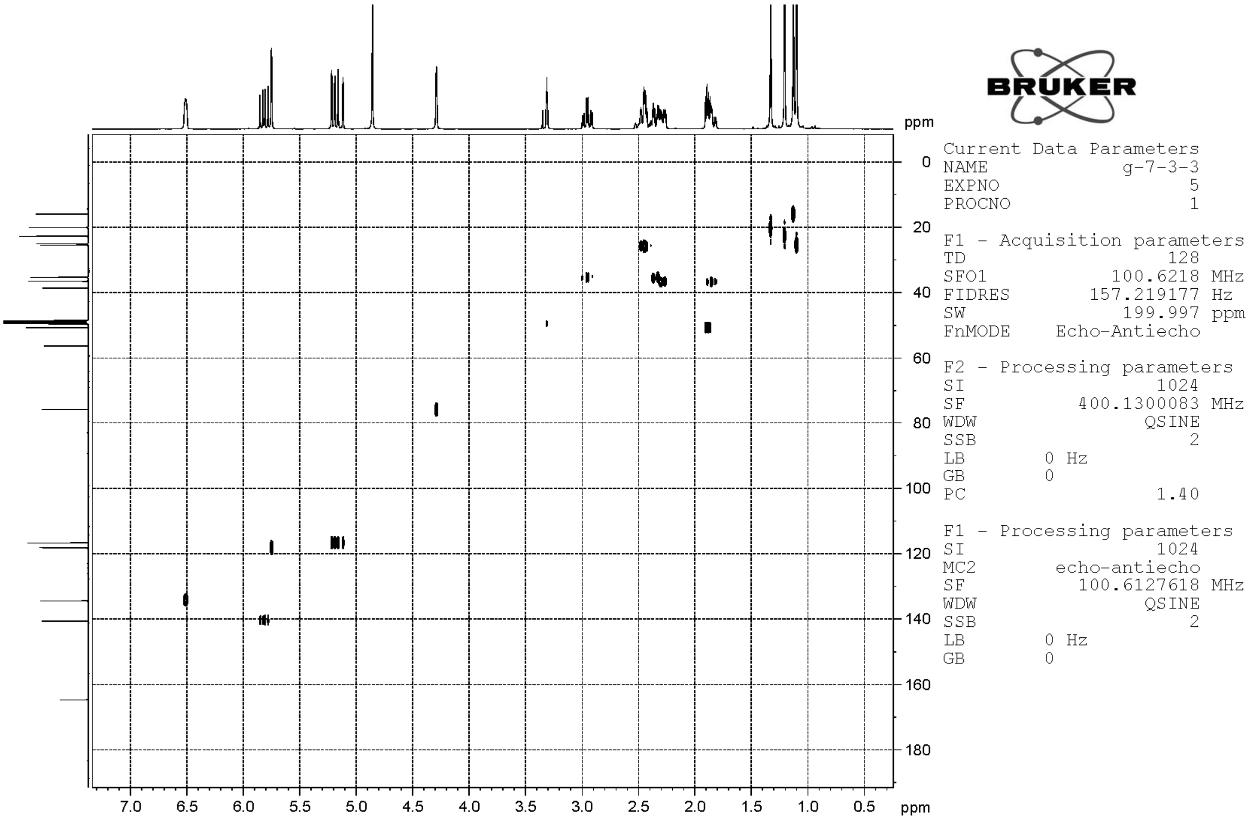

Figure S8. HMBC spectrum of compound **1** in CD_3OD.

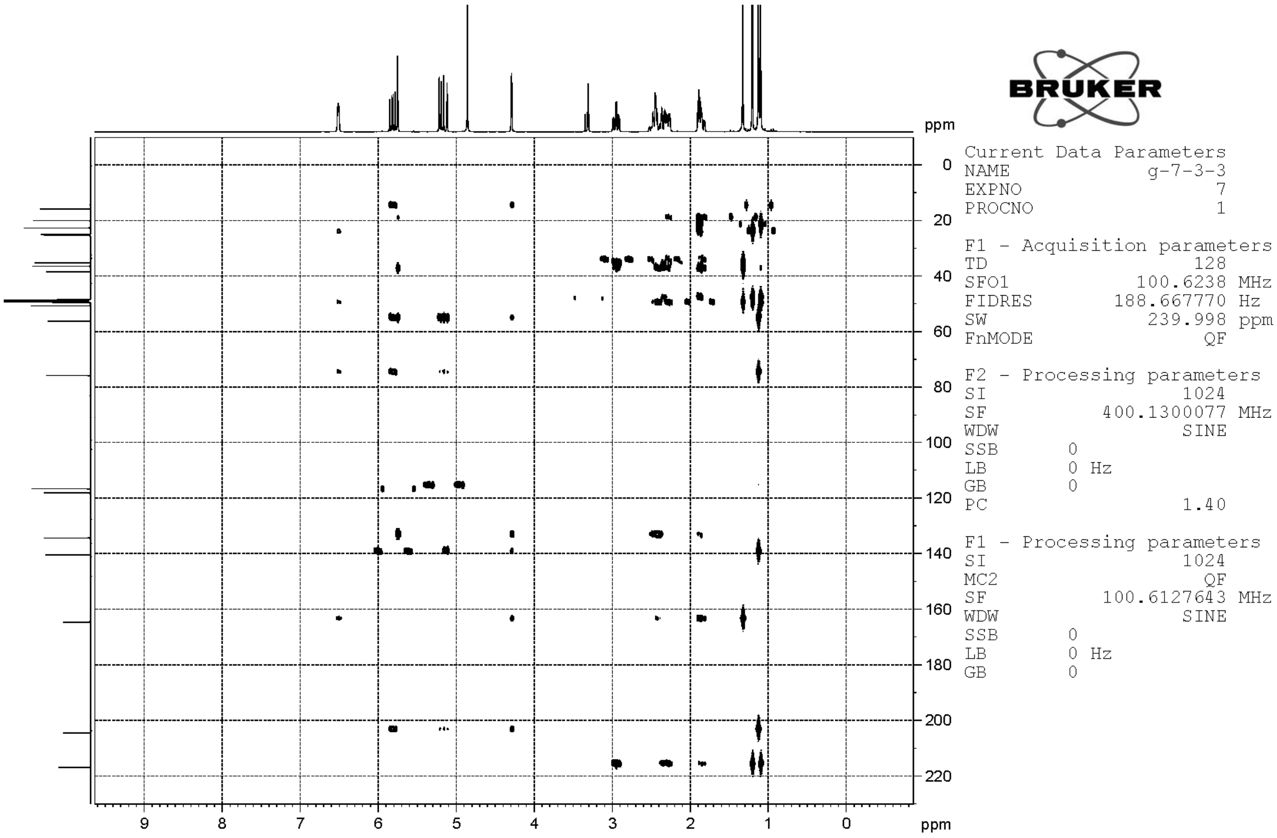

Figure S9. NOESY spectrum of compound **1** in CD_3OD.

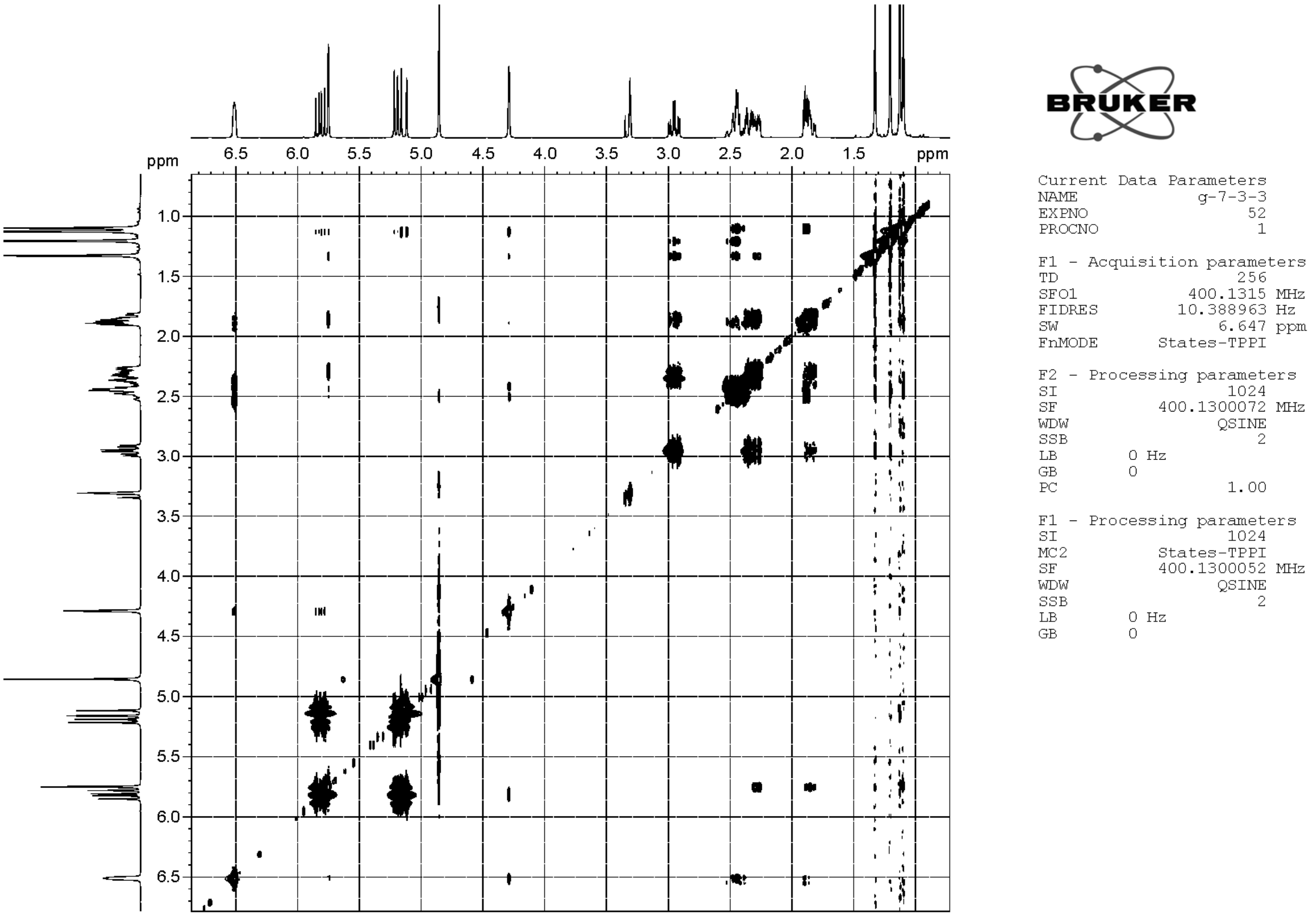

Figure S10. UV spectrum of compound **2** in CH_3OH.

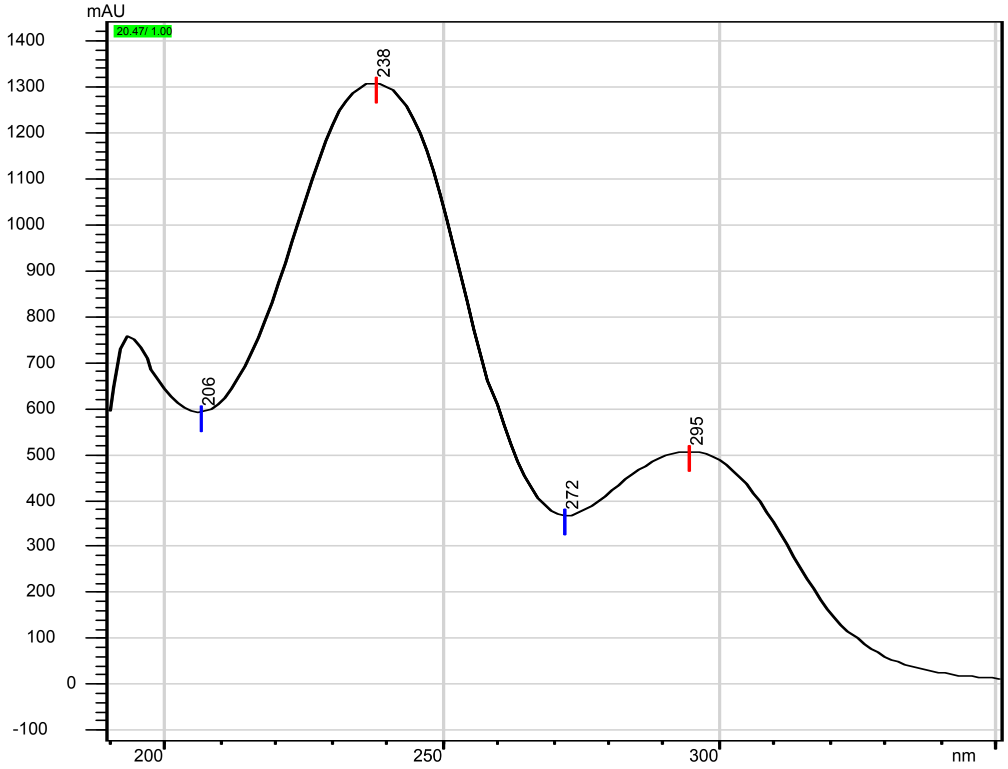

Figure S11. IR spectrum of compound **2**.

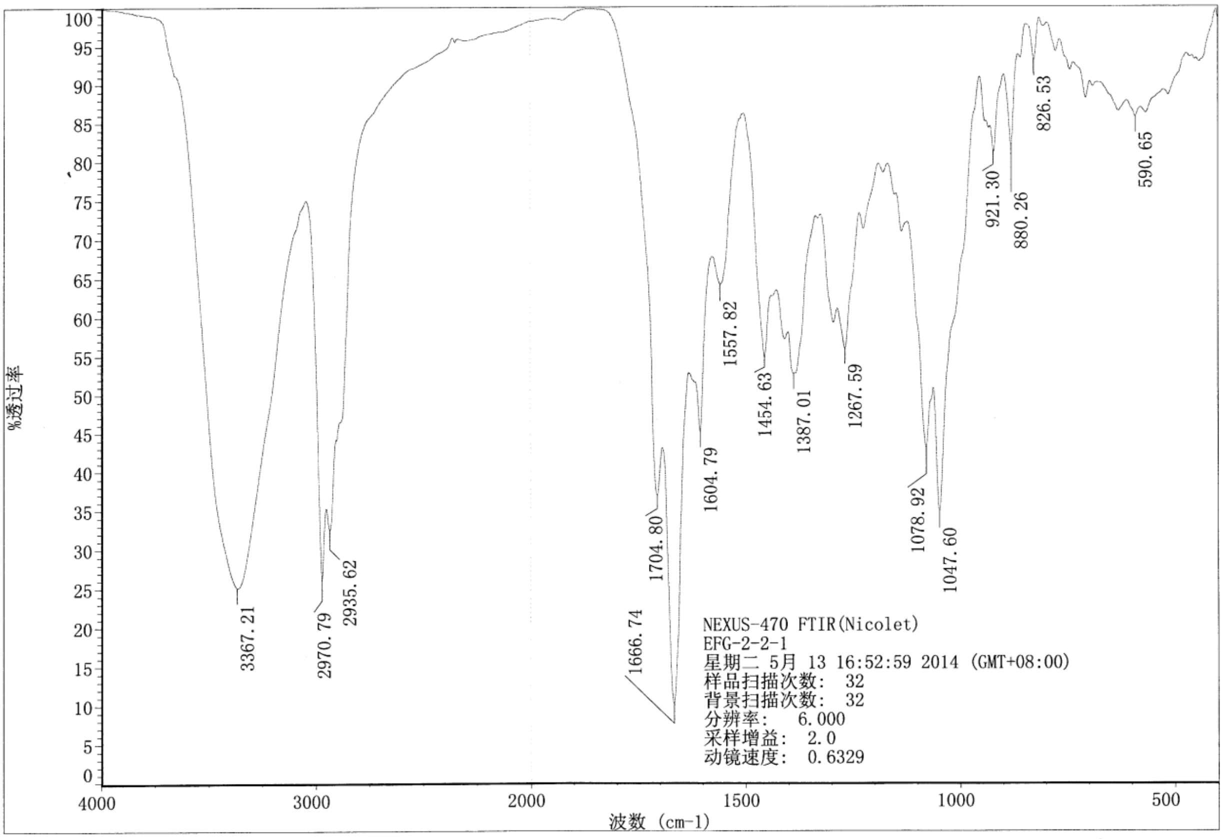

Figure S12. HR-ESI-TOF-MS spectrum of compound **2**.

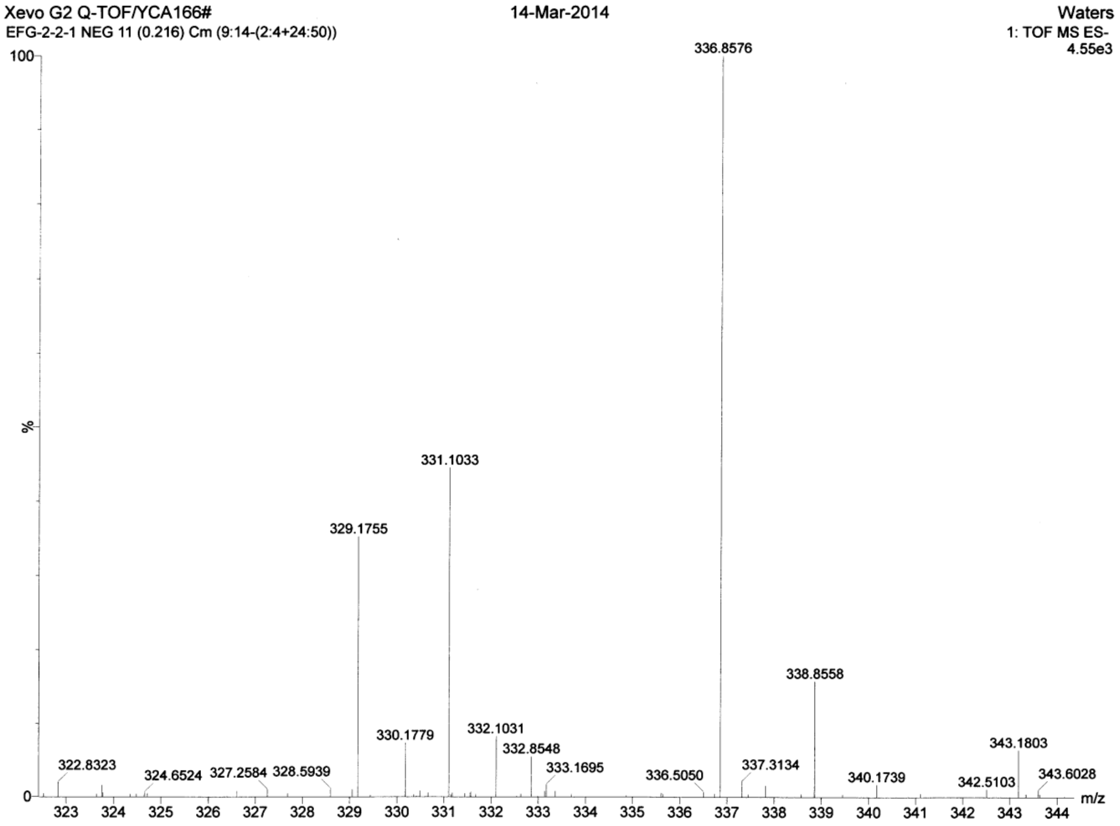

Figure S13. ^{1}H-NMR spectrum of compound **2** in $CDCl_3$.

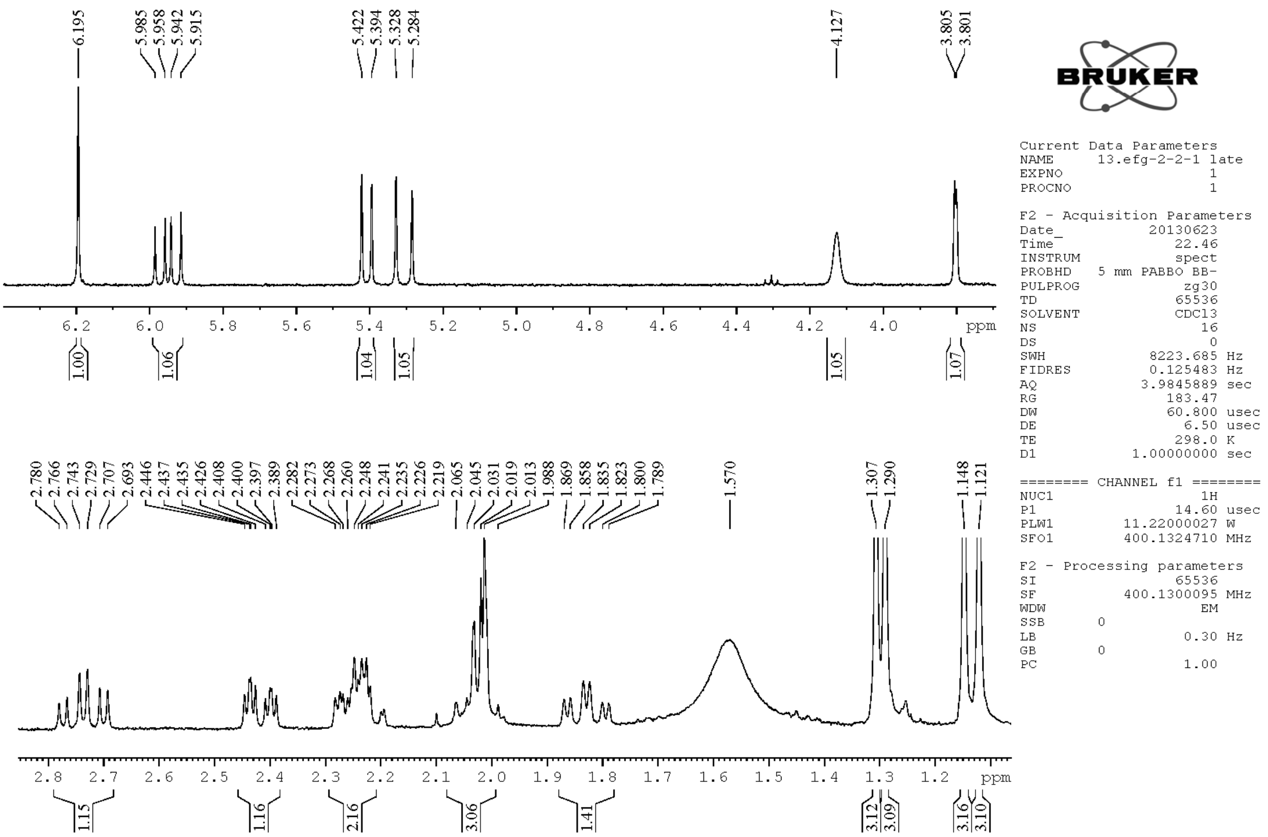

Figure S14. ^{13}C-NMR spectrum of compound **2** in $CDCl_3$.

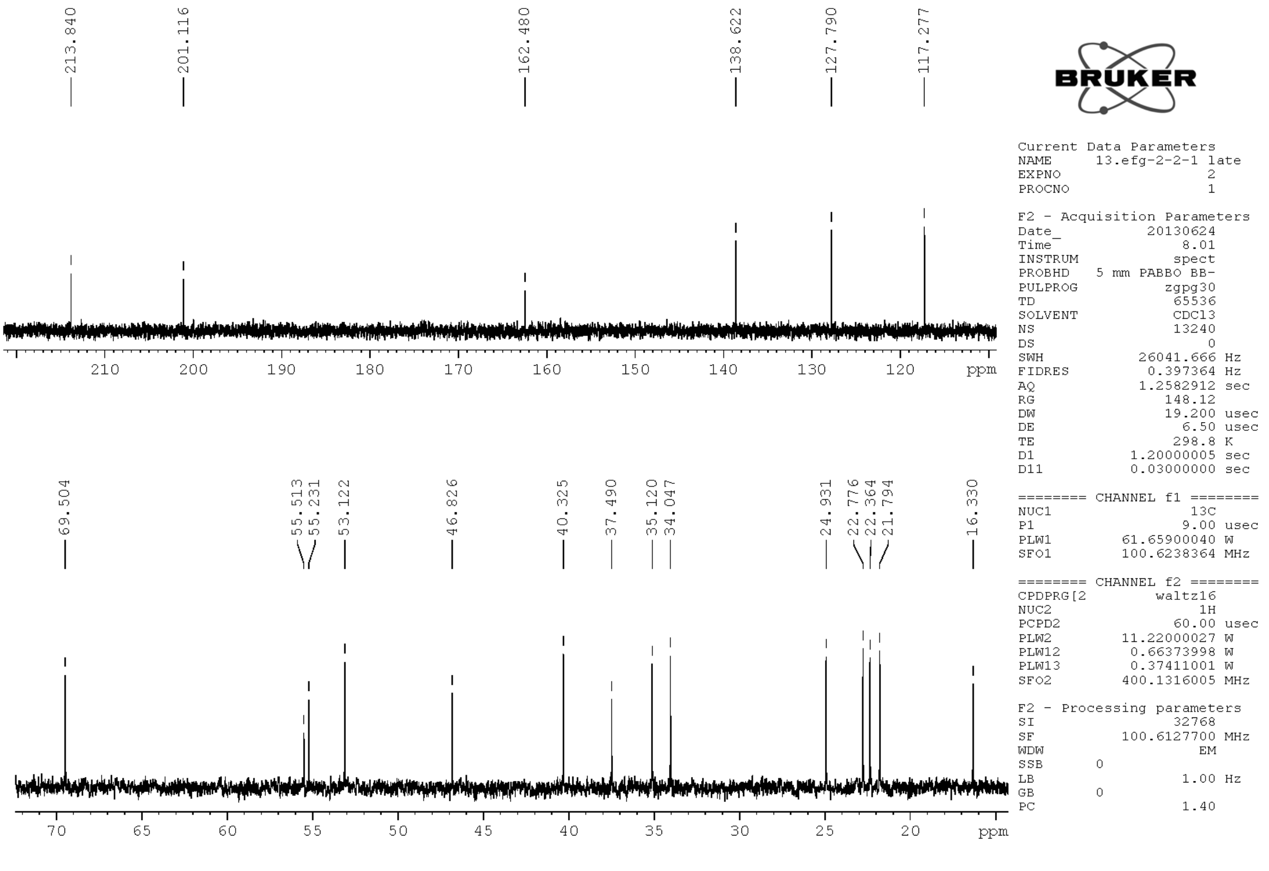

Figure S15. $^{1}H^{1}H$ COSY spectrum of compound **2** in $CDCl_3$.

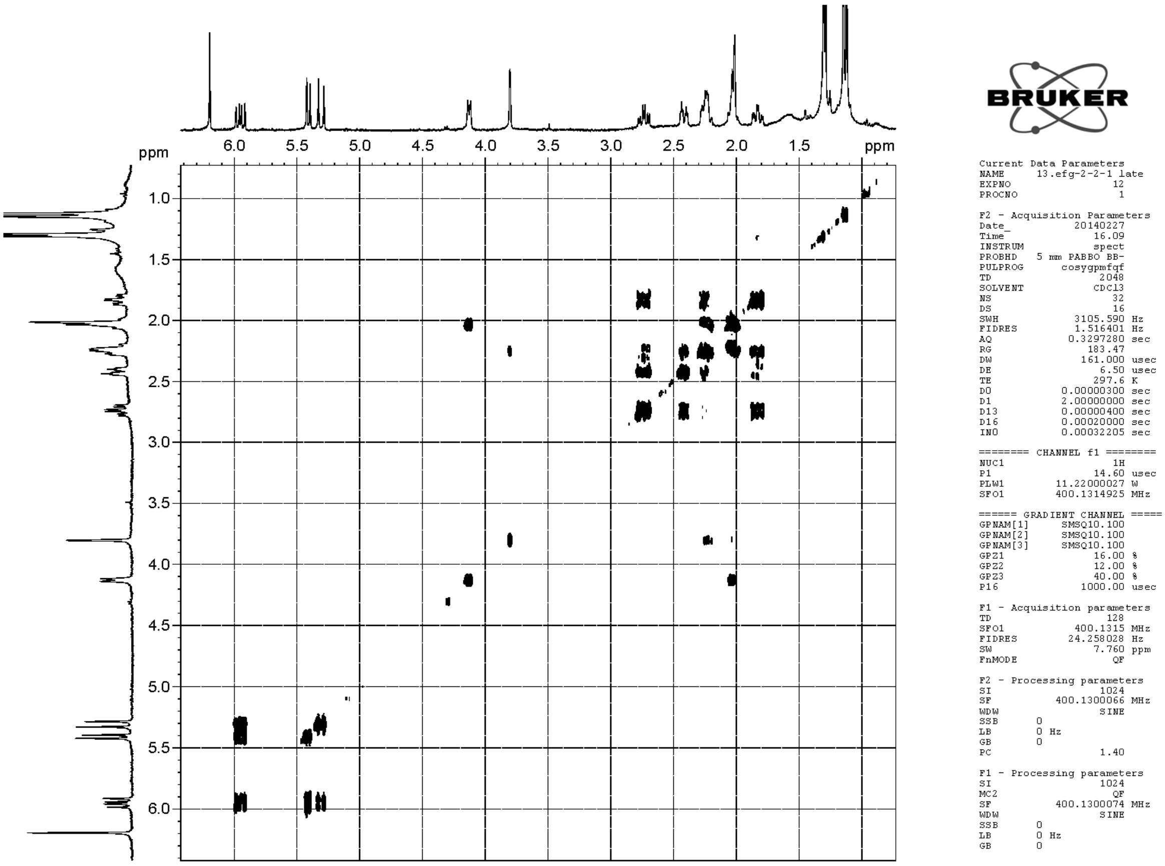

Figure S16. HSQC spectrum of compound **2** in $CDCl_3$.

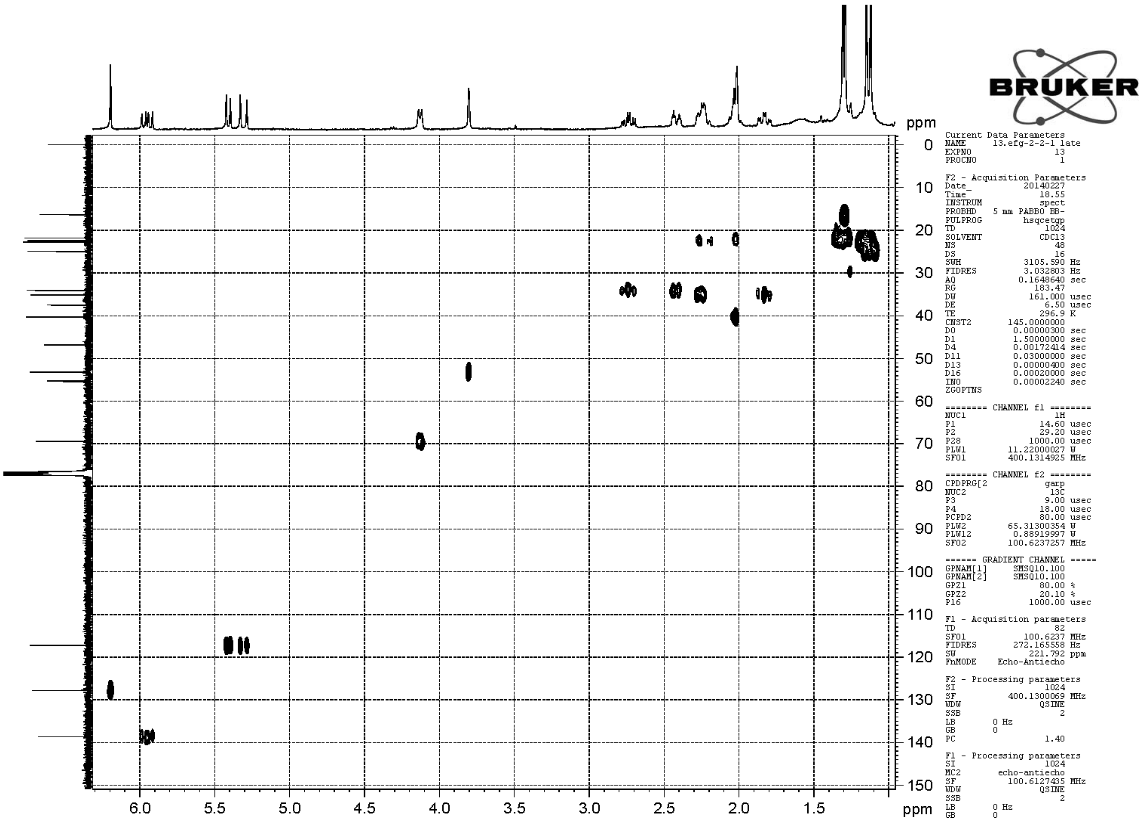

Figure S17. HMBC spectrum of compound **2** in $CDCl_3$.

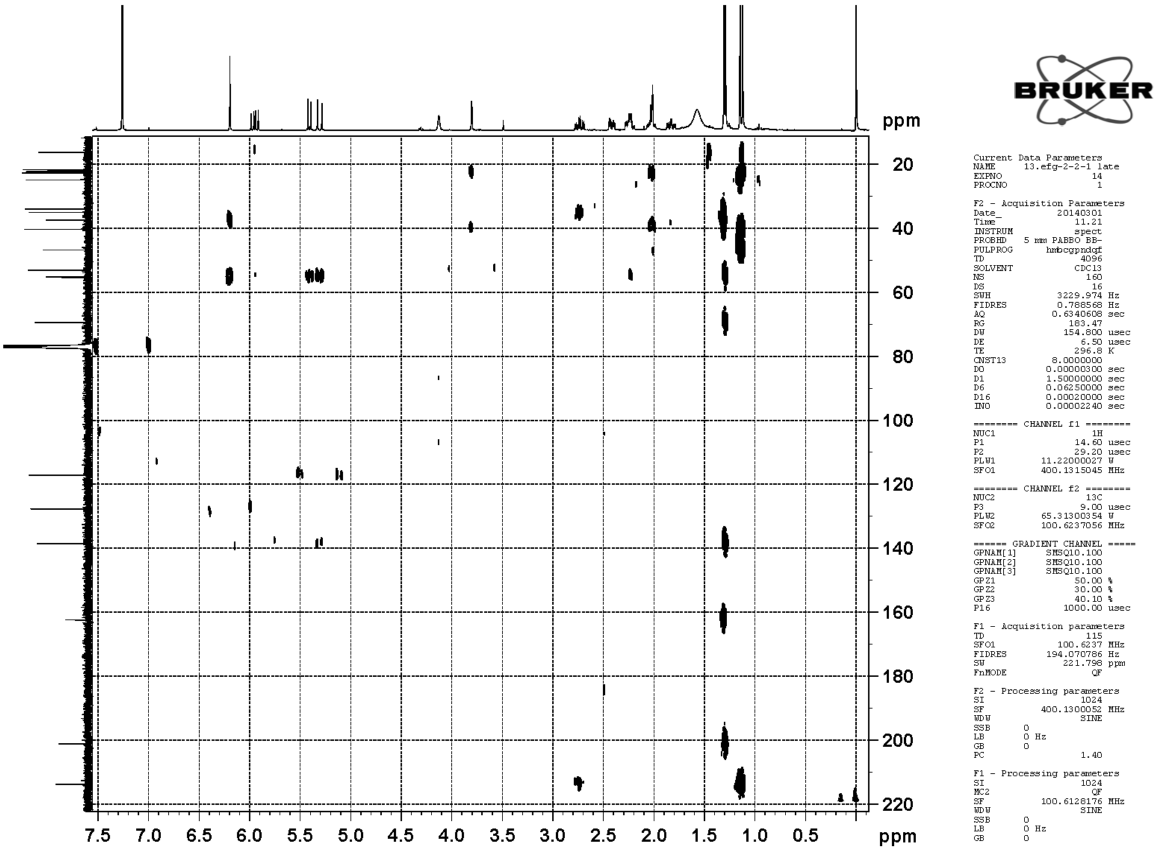

Figure S18. NOESY spectrum of compound **2** in $CDCl_3$.

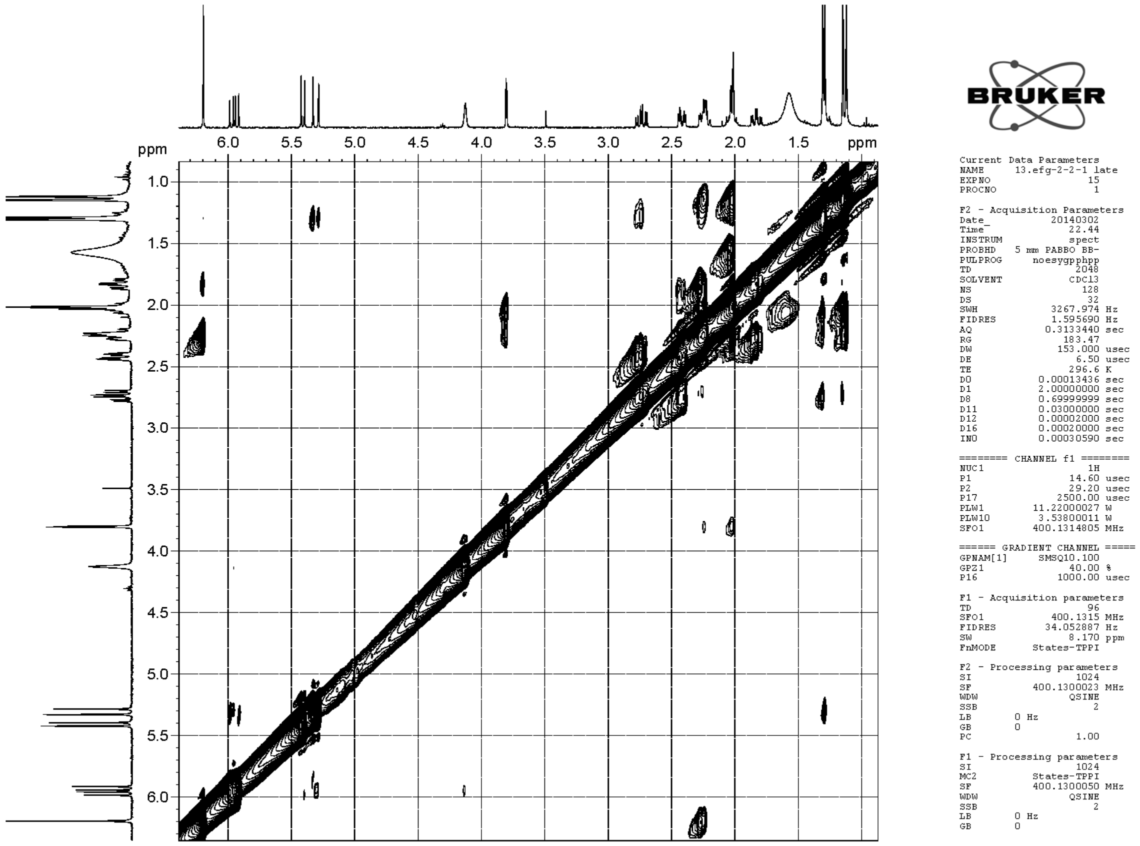

Figure S19. UV spectrum of compound **3** in CH_3OH.

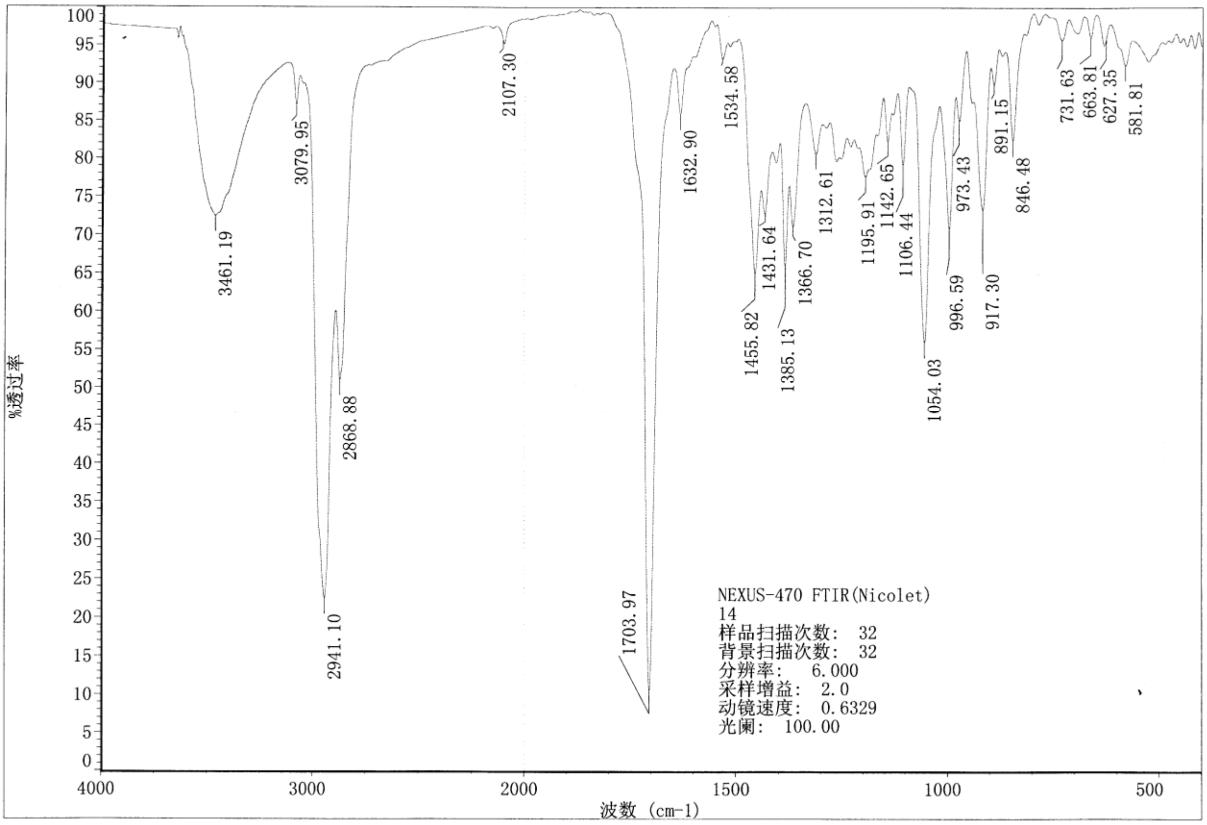

Figure S20. IR spectrum of compound **3**.

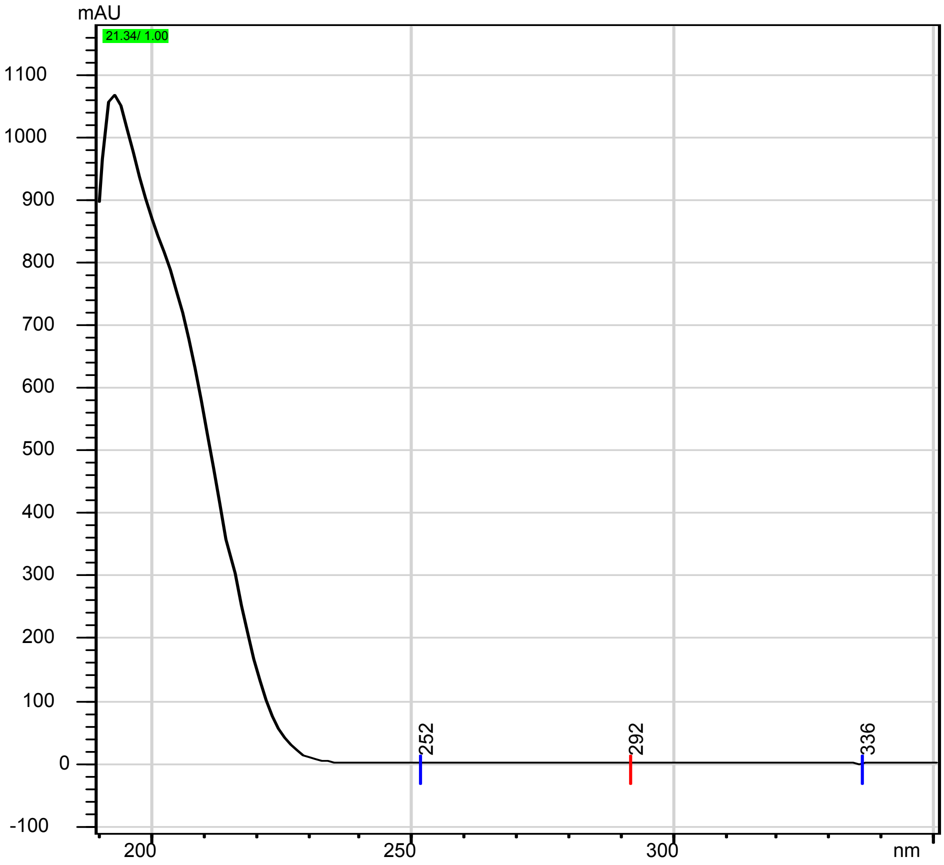

Figure S21. HR-ESI-TOF-MS spectrum of compound **3**.

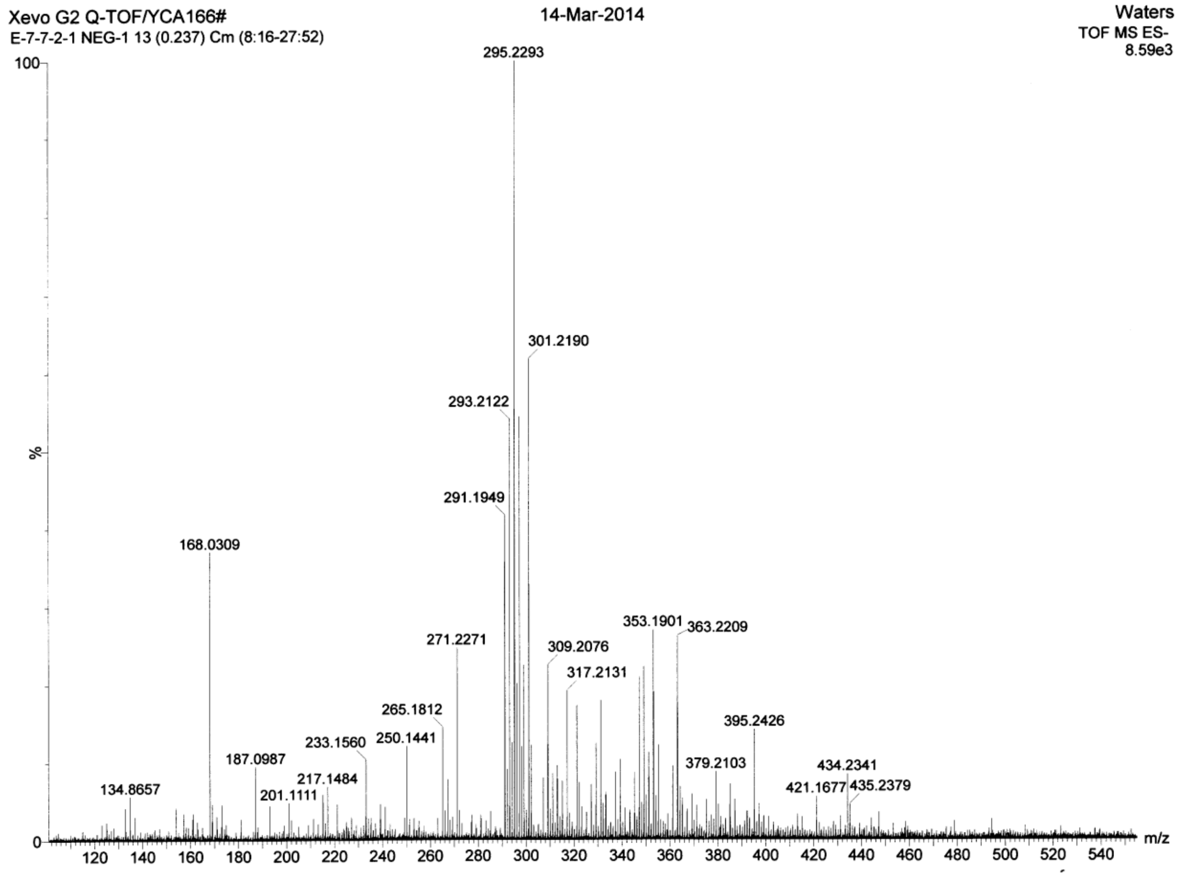

Figure S22. ^{1}H-NMR spectrum of compound **3** in $CDCl_3$.

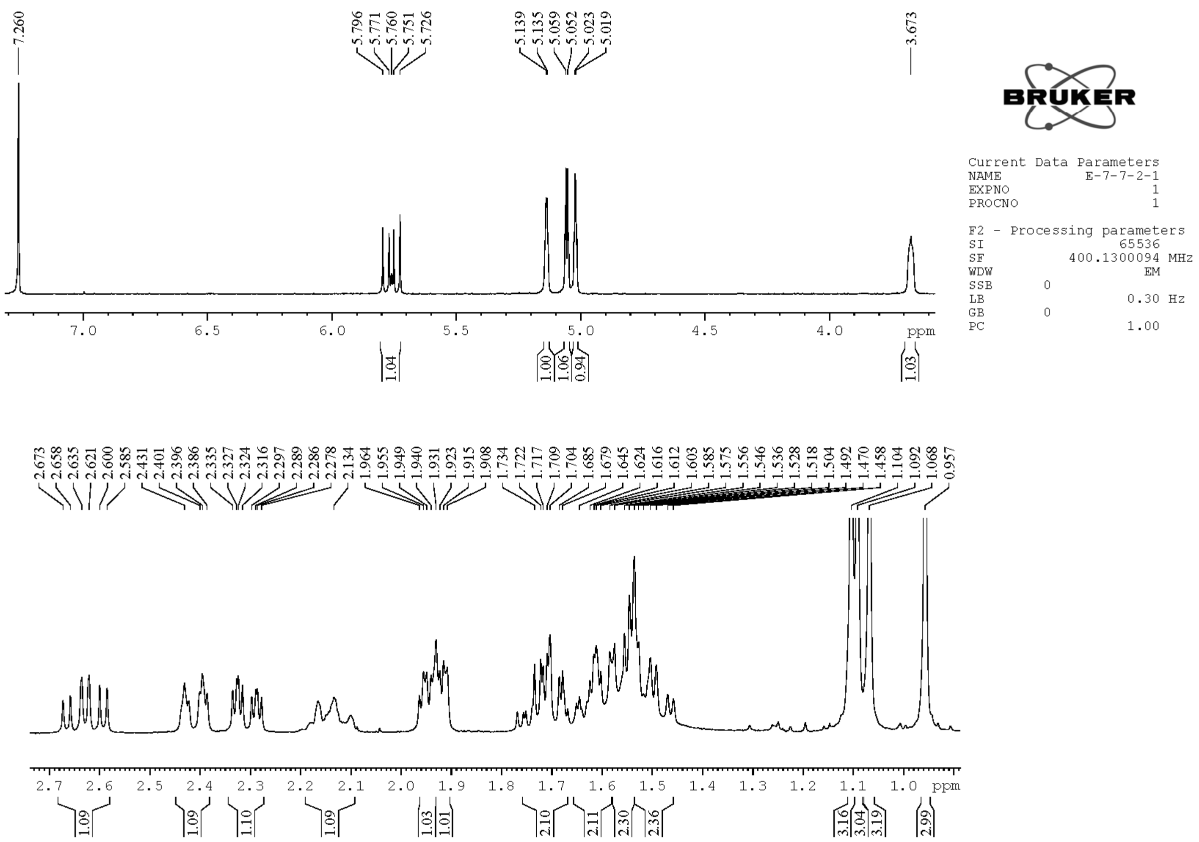

Figure S23. ^{13}C-NMR spectrum of compound **3** in $CDCl_3$.

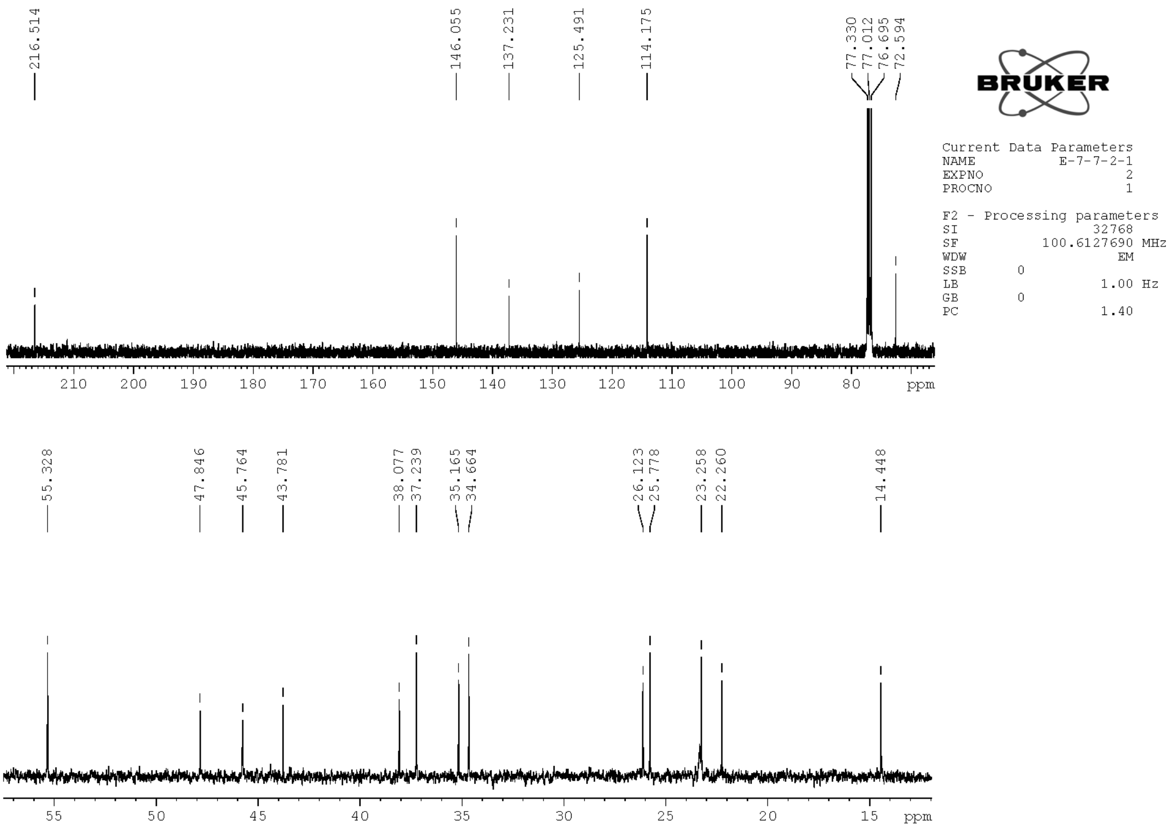

Figure S24. ^{1}H^{1}H COSY spectrum of compound **3** in $CDCl_3$.

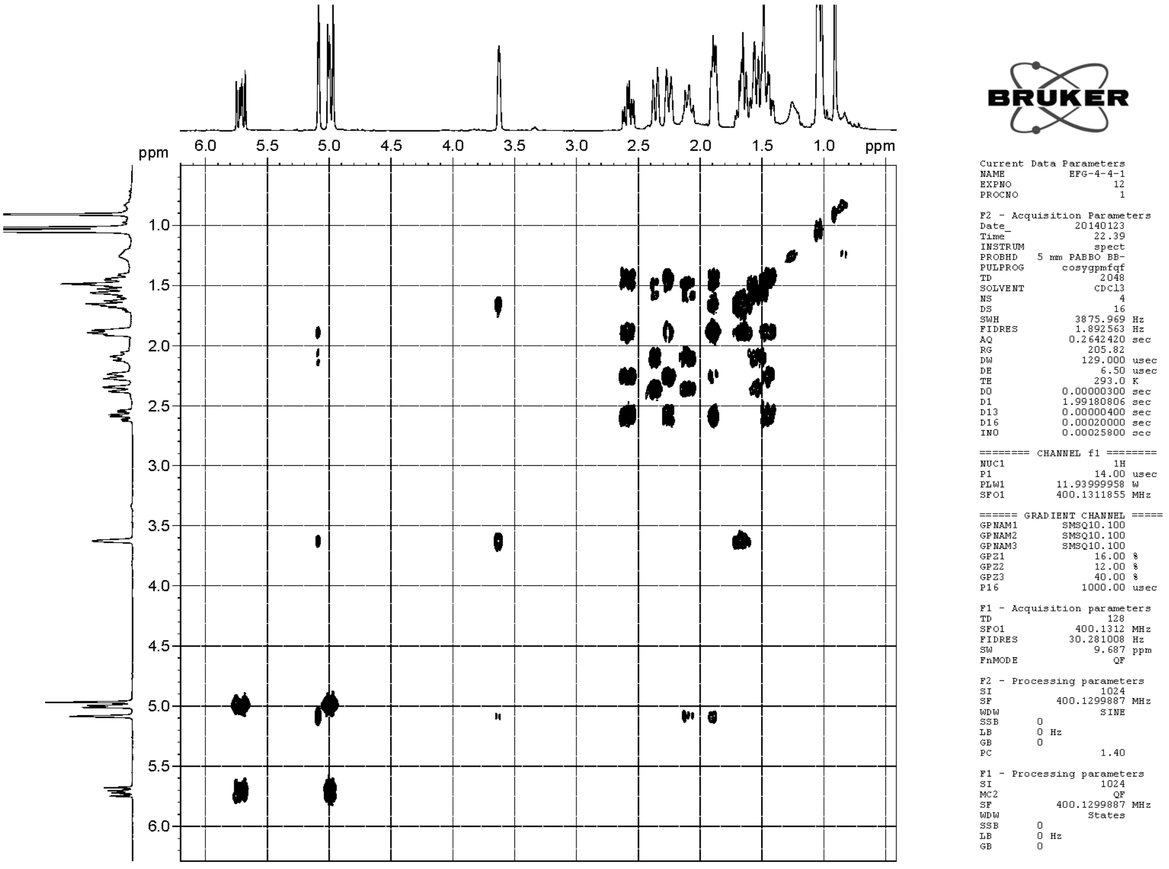

Figure S25. HSQC spectrum of compound **3** in $CDCl_3$.

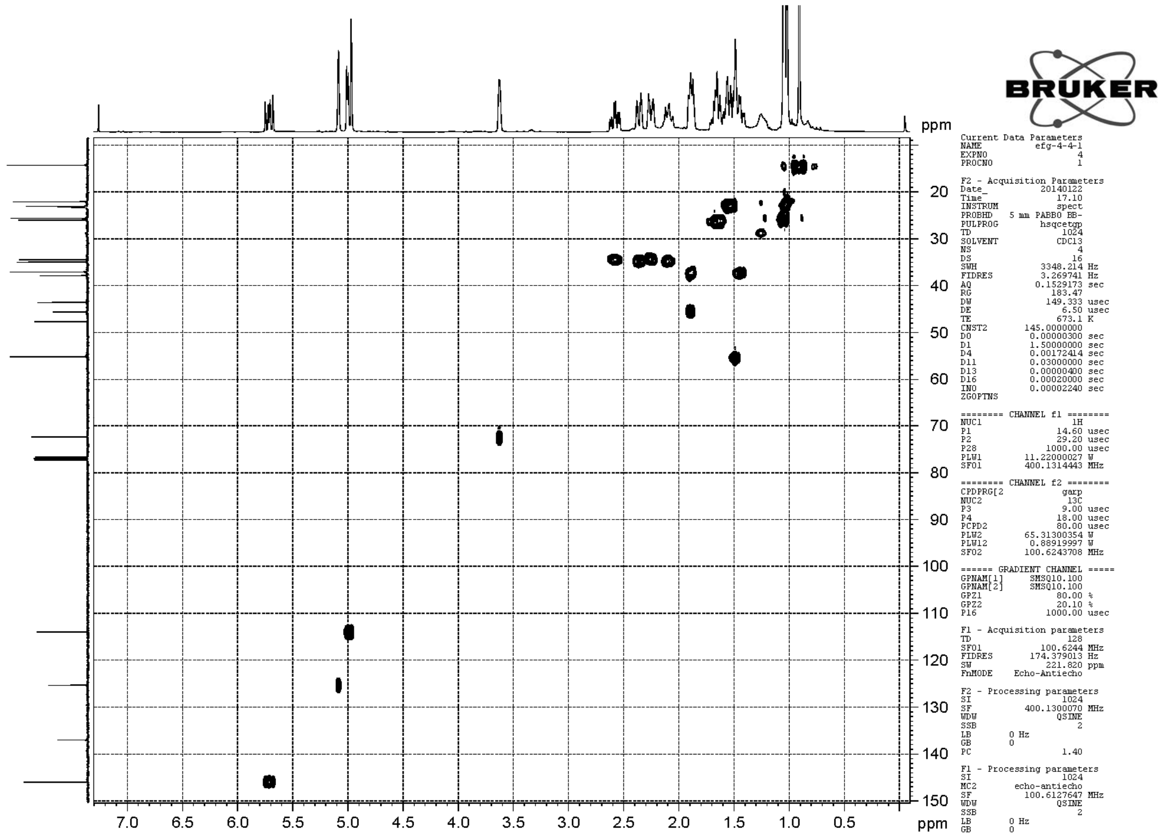

Figure S26. HMBC spectrum of compound **3** in $CDCl_3$.

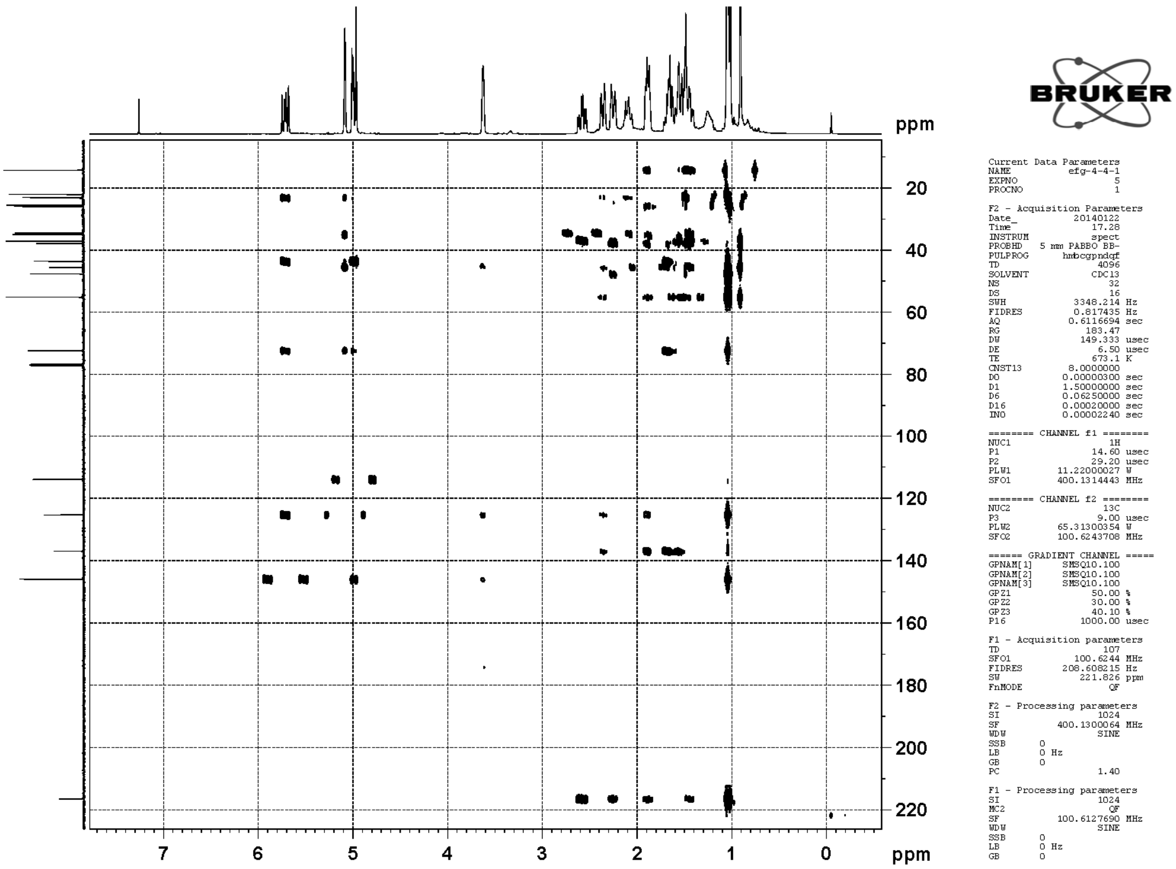

Figure S27. NOESY spectrum of compound **3** in $CDCl_3$.

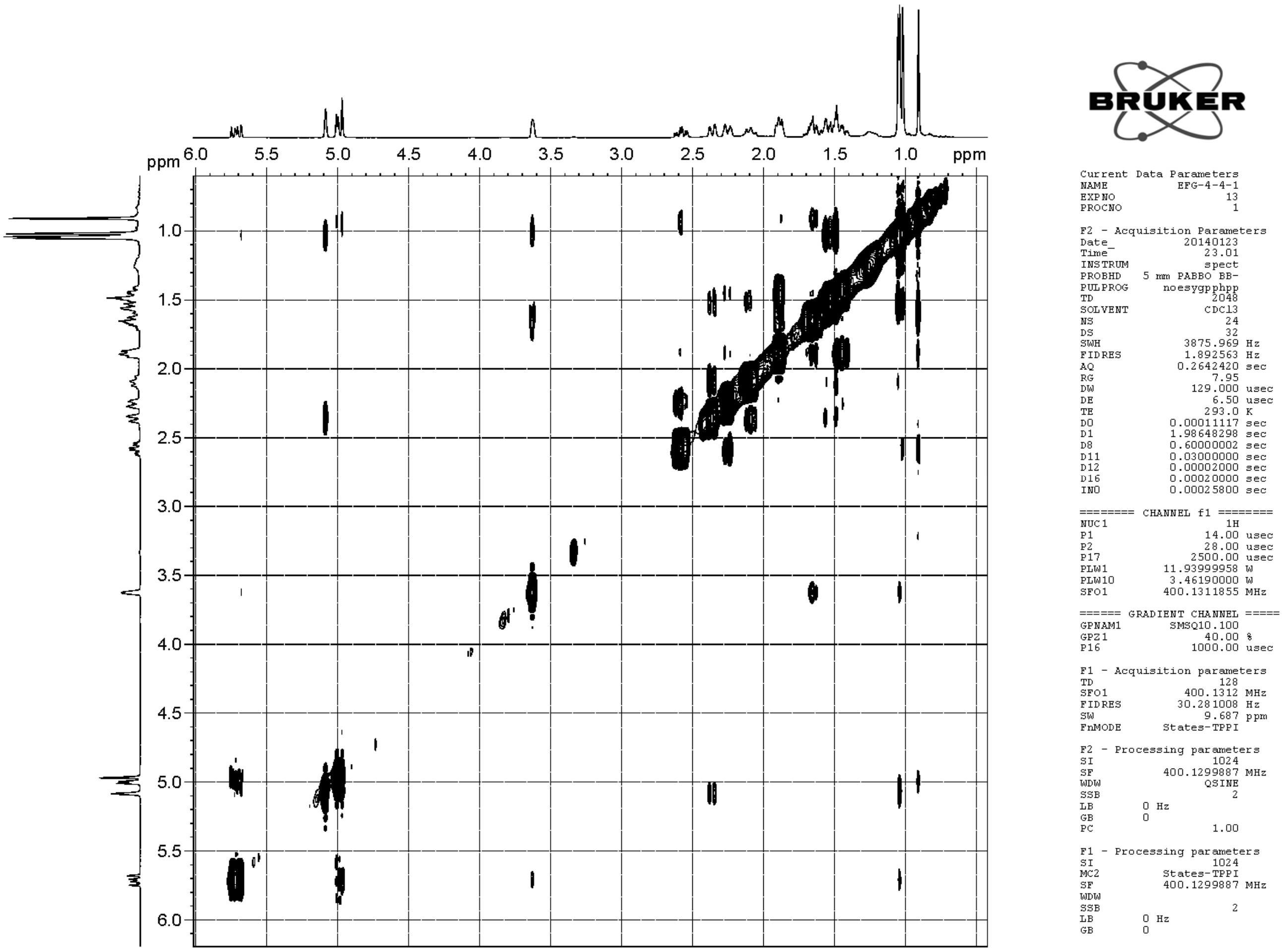

Figure S28. UV spectrum of compound **4** in CH_3OH.

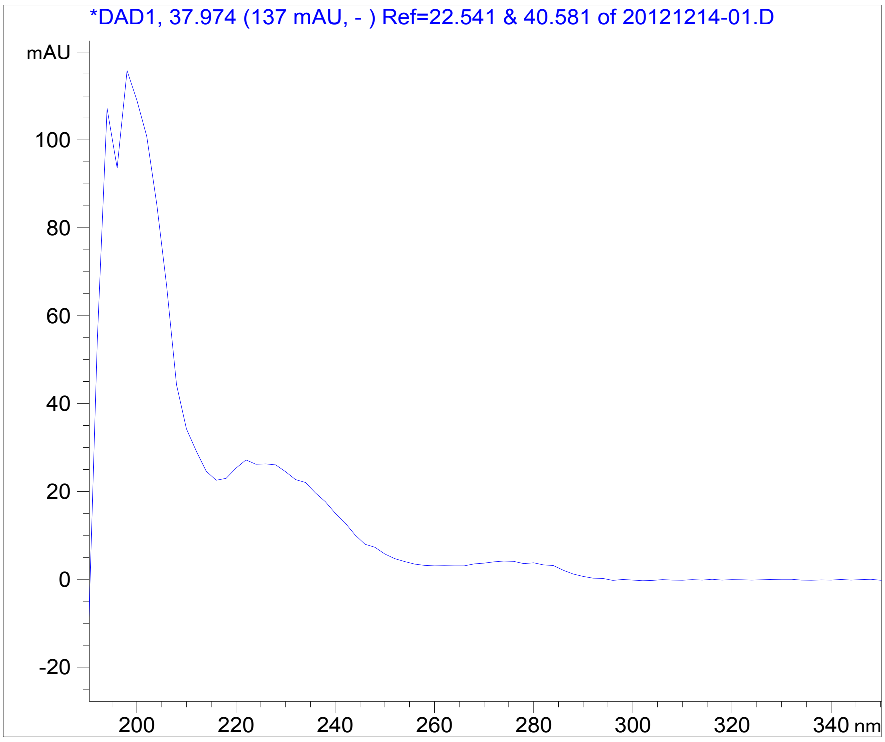

Figure S29. IR spectrum of compound **4**.

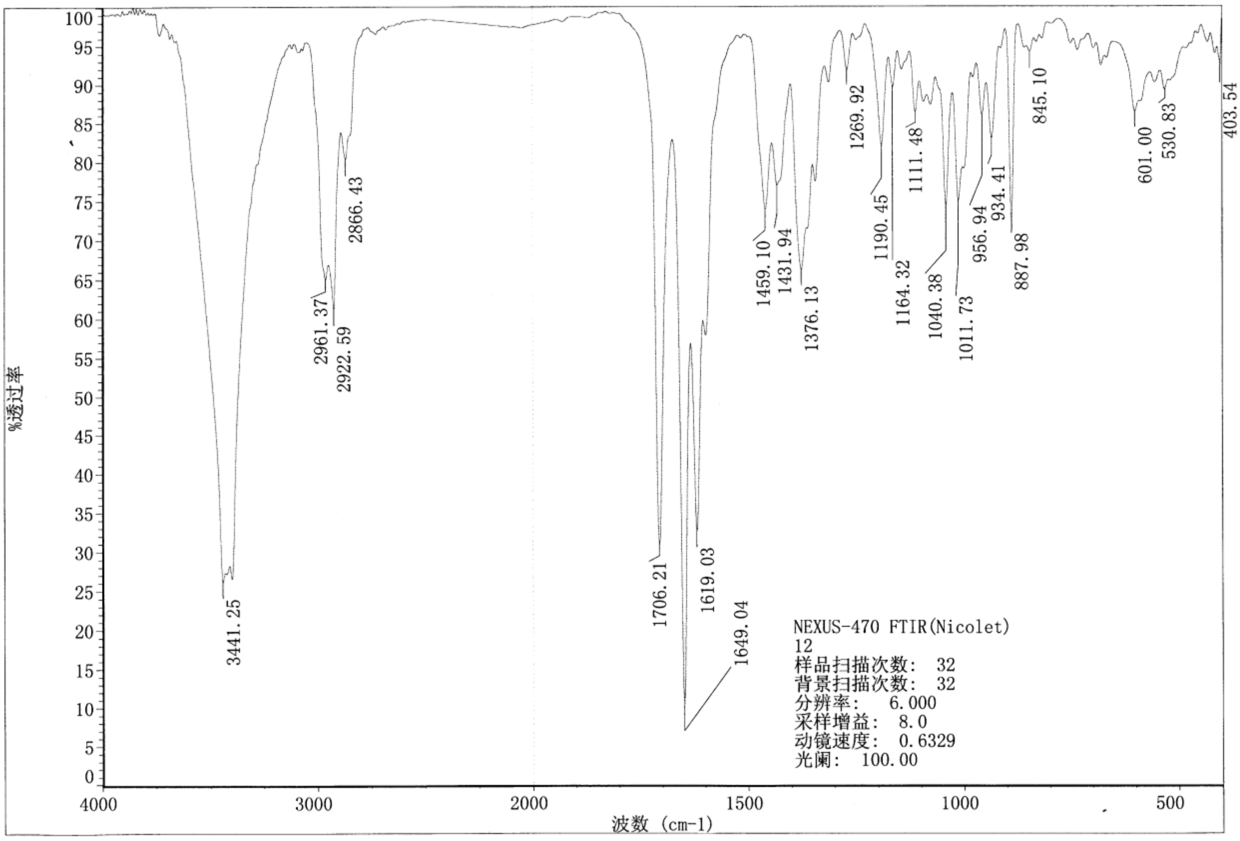

Figure S30. HR-ESI-TOF-MS spectrum of compound **4**.

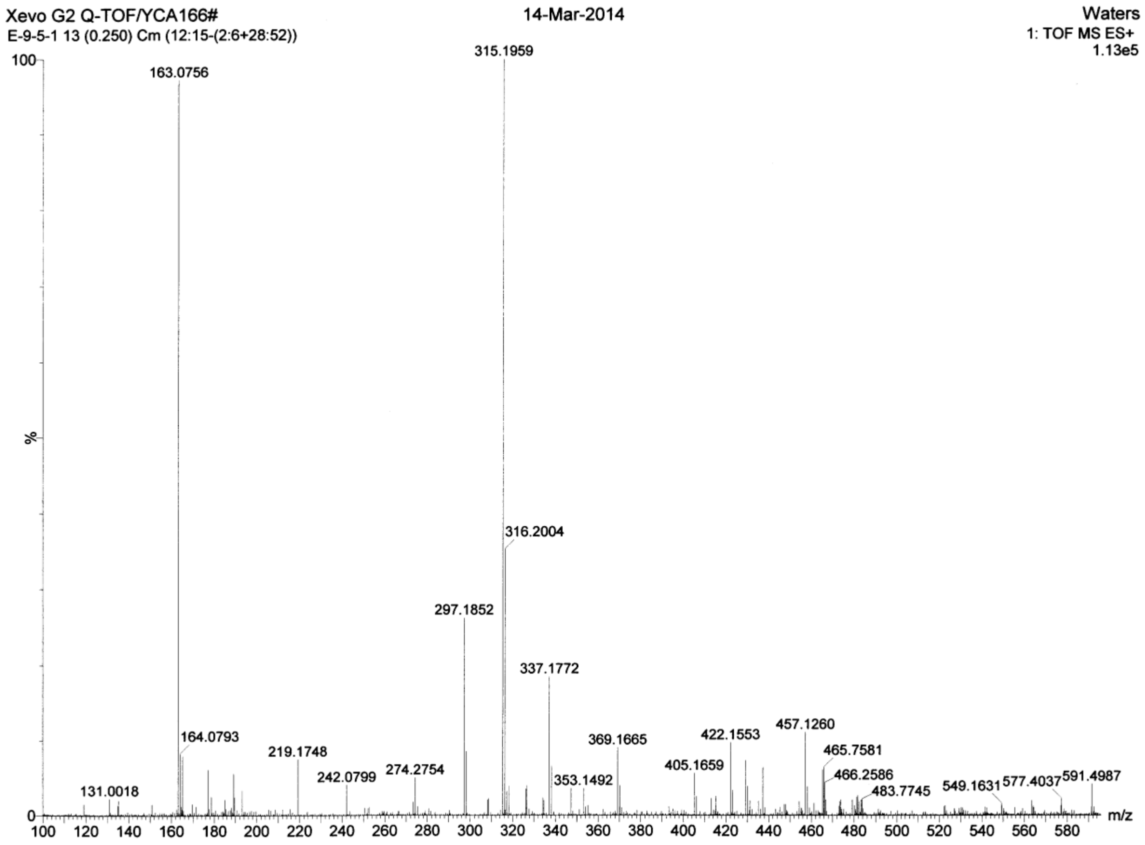

Figure S31. ^{1}H-NMR spectrum of compound **4** in CD_3OD.

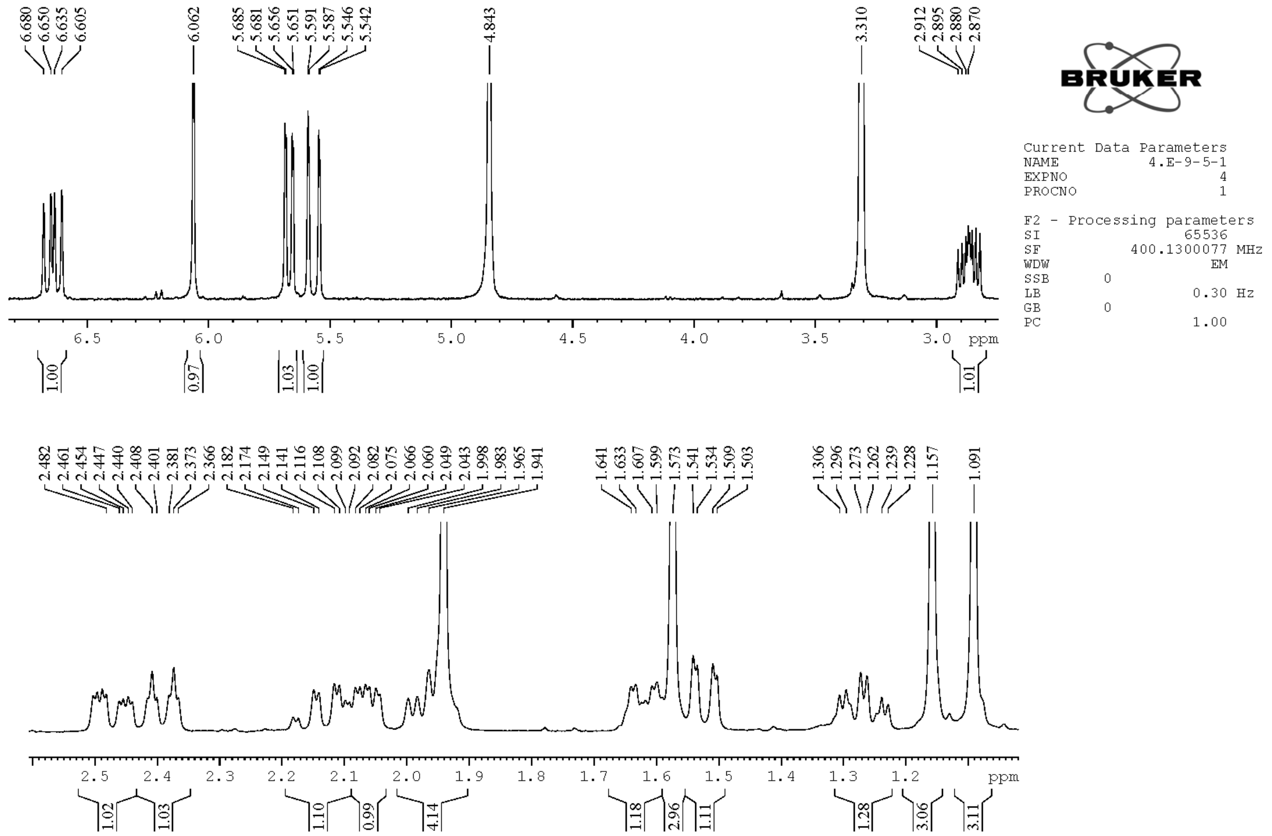

Figure S32. ^{13}C-NMR spectrum of compound **4** in CD_3OD.

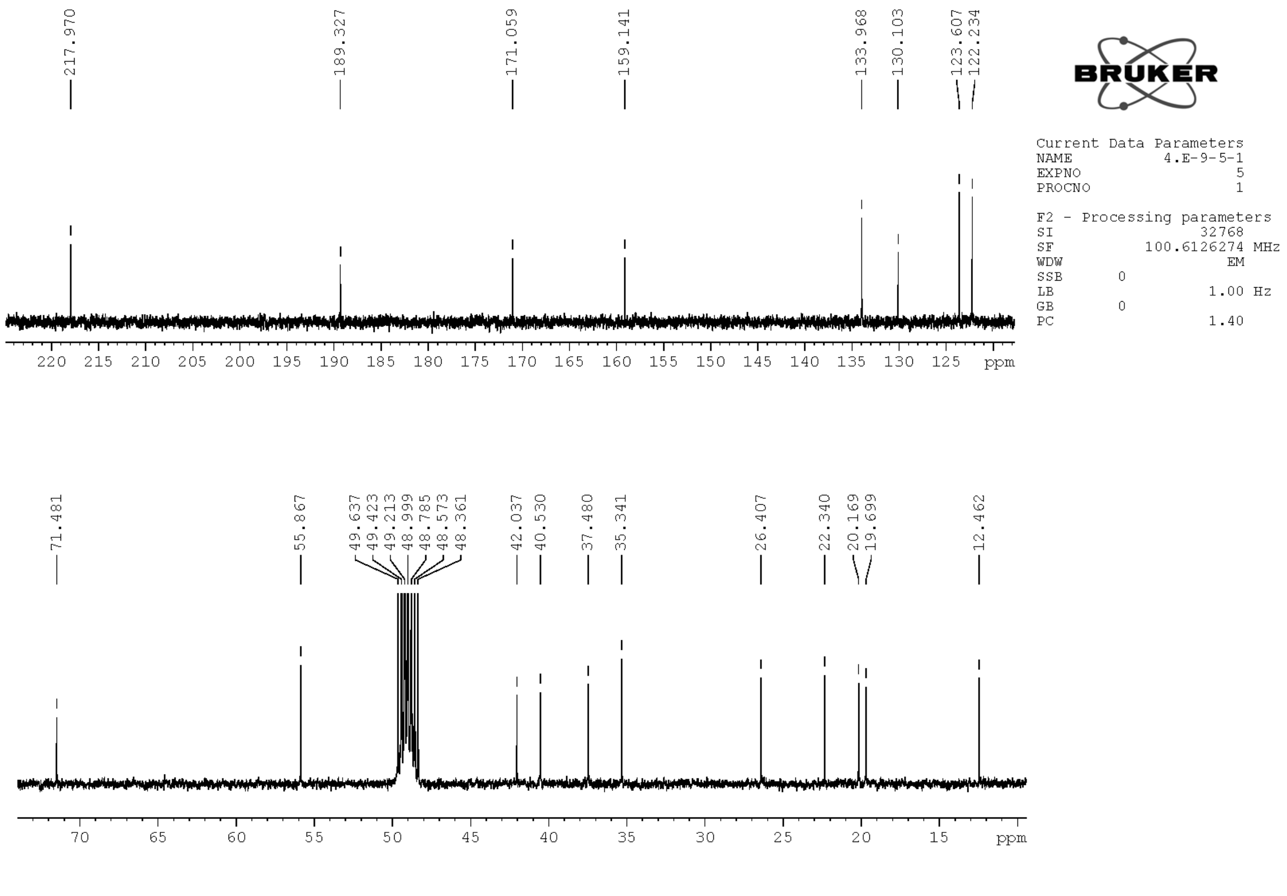

Figure S33. $^{1}H^{1}H$ COSY spectrum of compound **4** in CD_3OD.

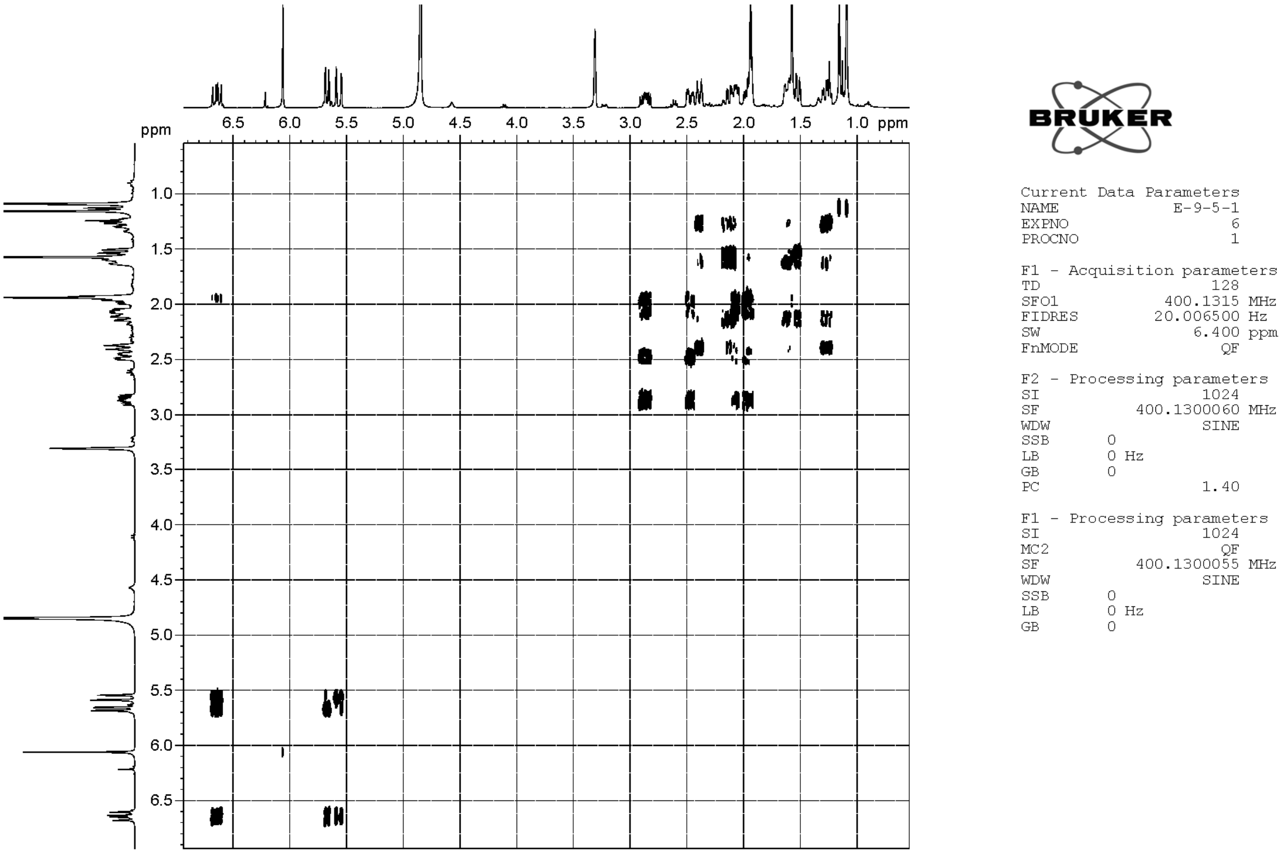

Figure S34. HSQC spectrum of compound **4** in CD_3OD.

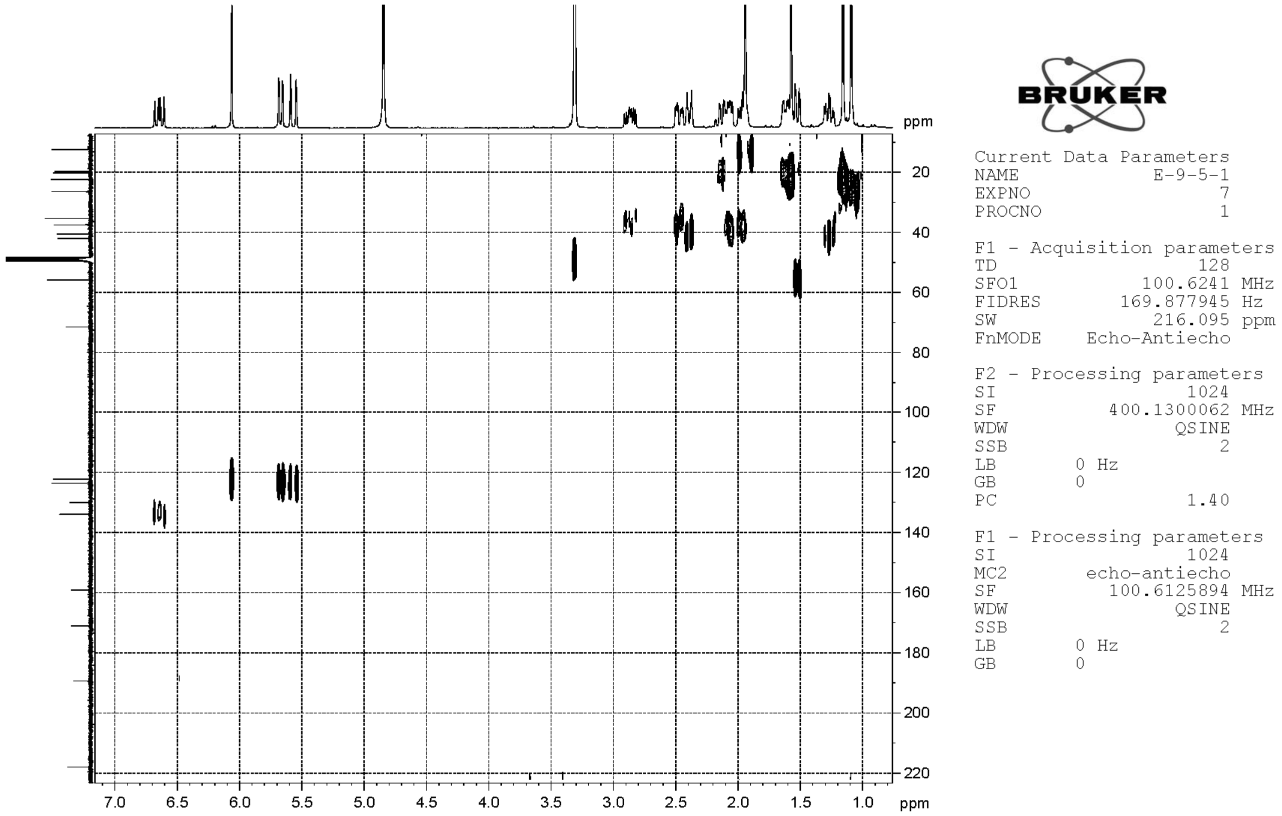

Figure S35. HMBC spectrum of compound **4** in CD_3OD.

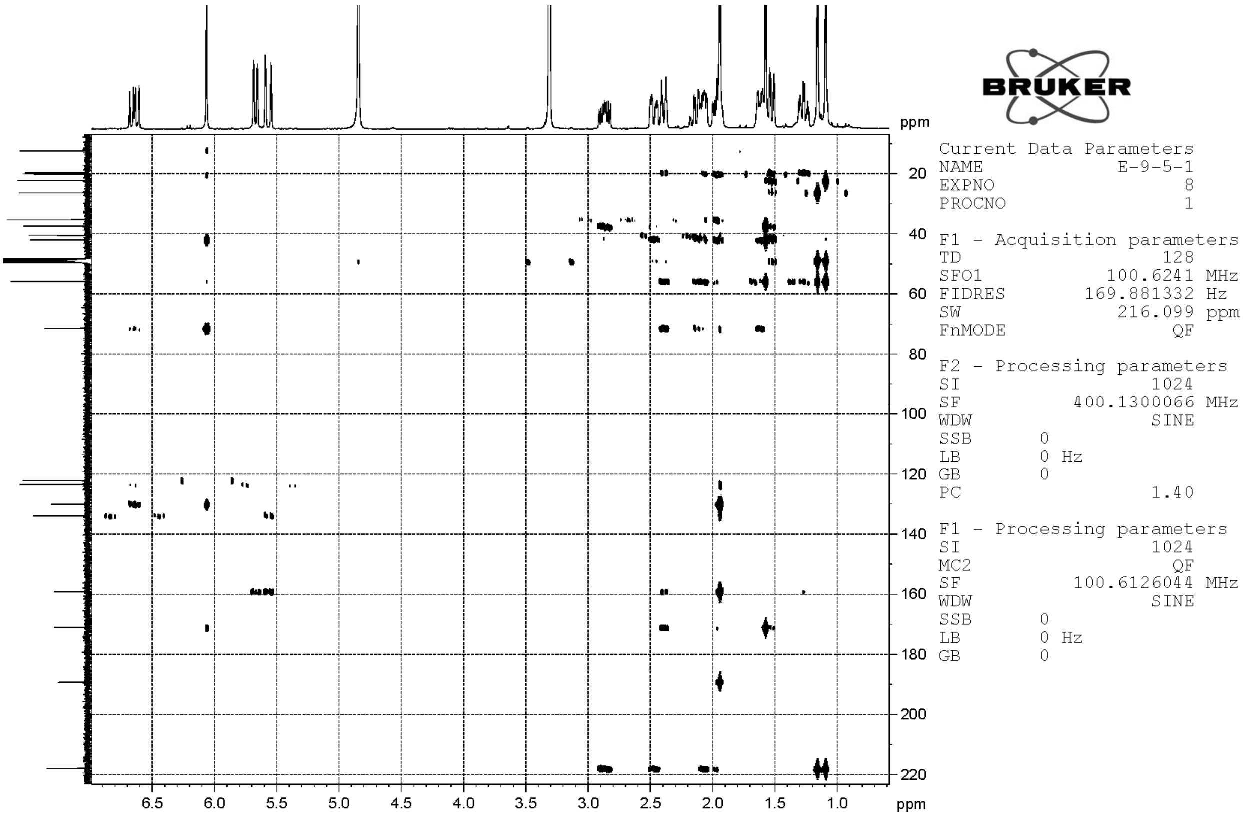

Figure S36. NOESY spectrum of compound **4** in CD_3OD.

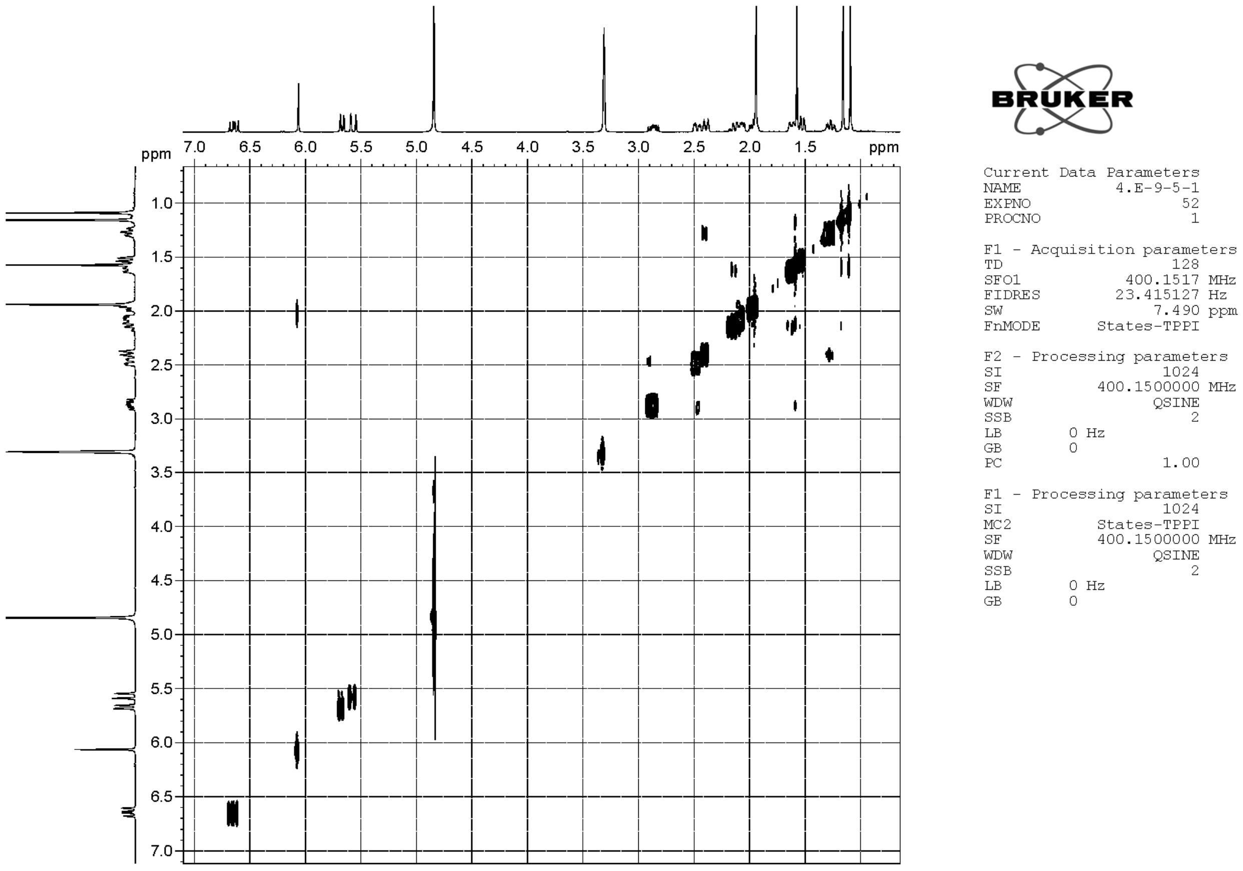

Figure S37. UV spectrum of compound **5** in CH_3OH.

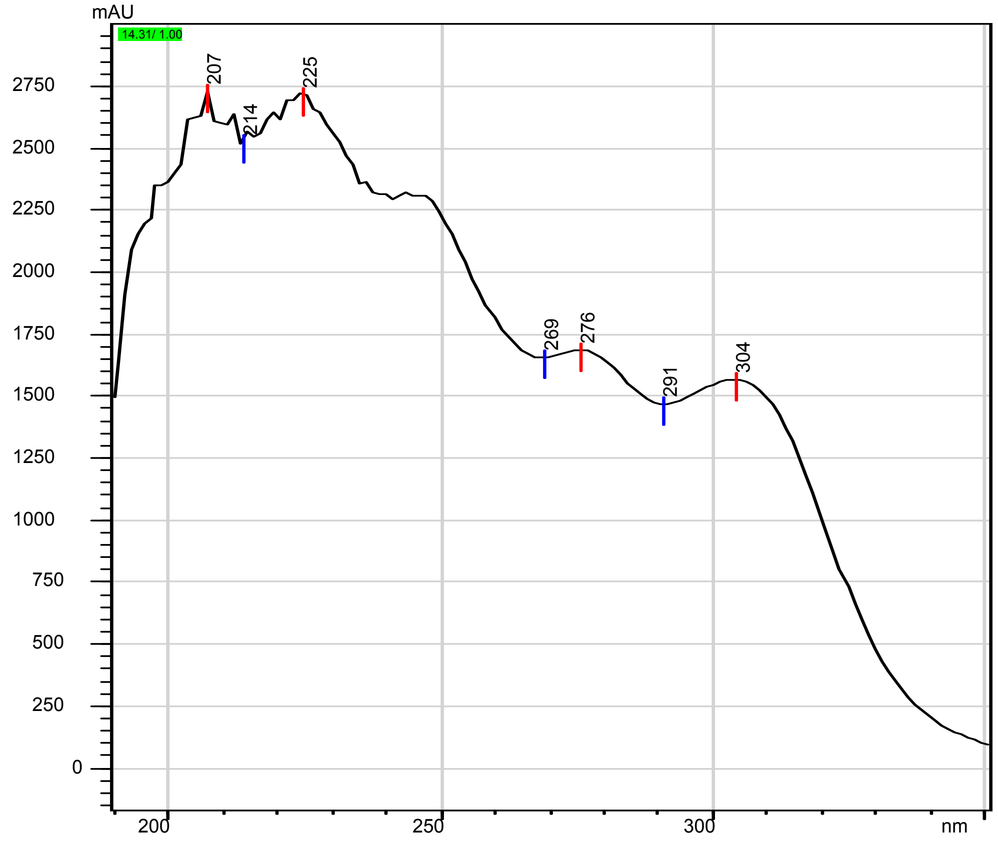

Figure S38. IR spectrum of compound **5**.

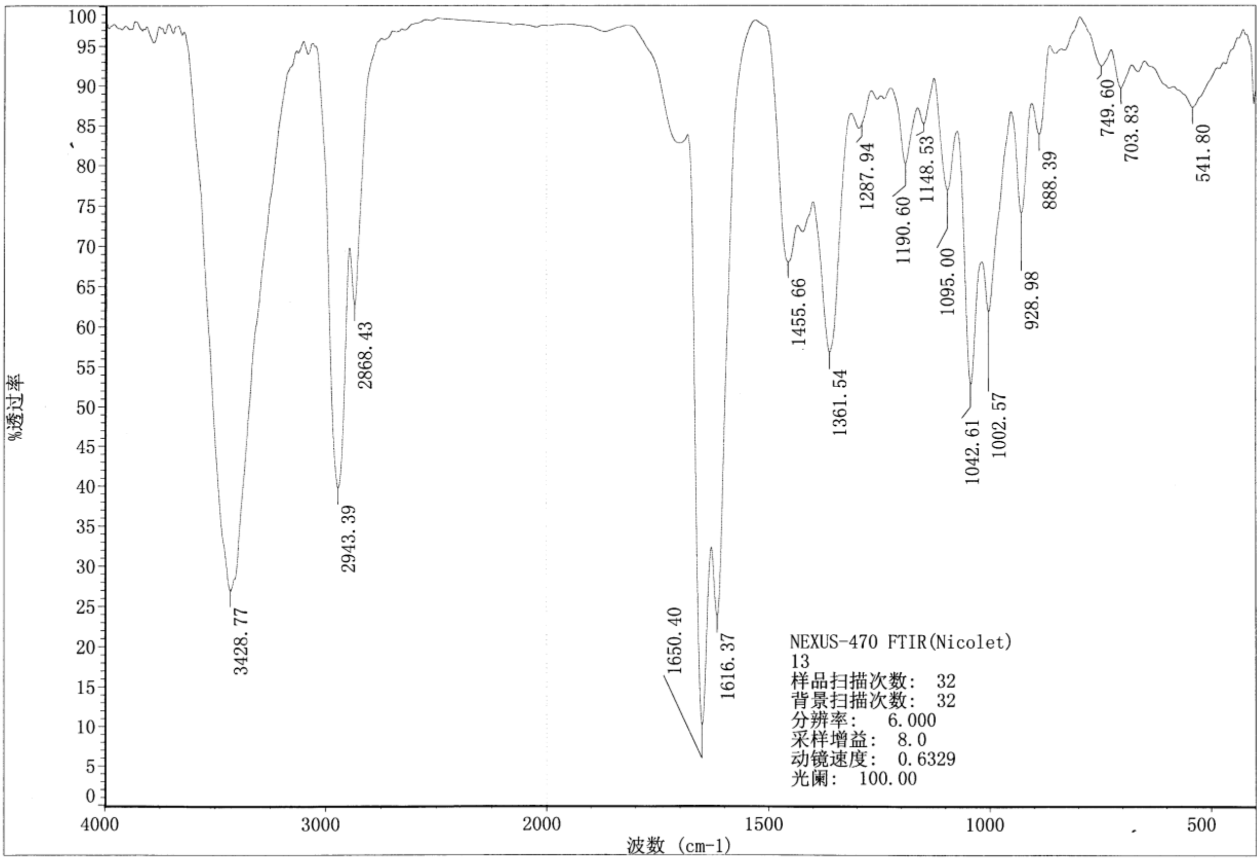

Figure S39. HR-ESI-TOF-MS spectrum of compound **5**.

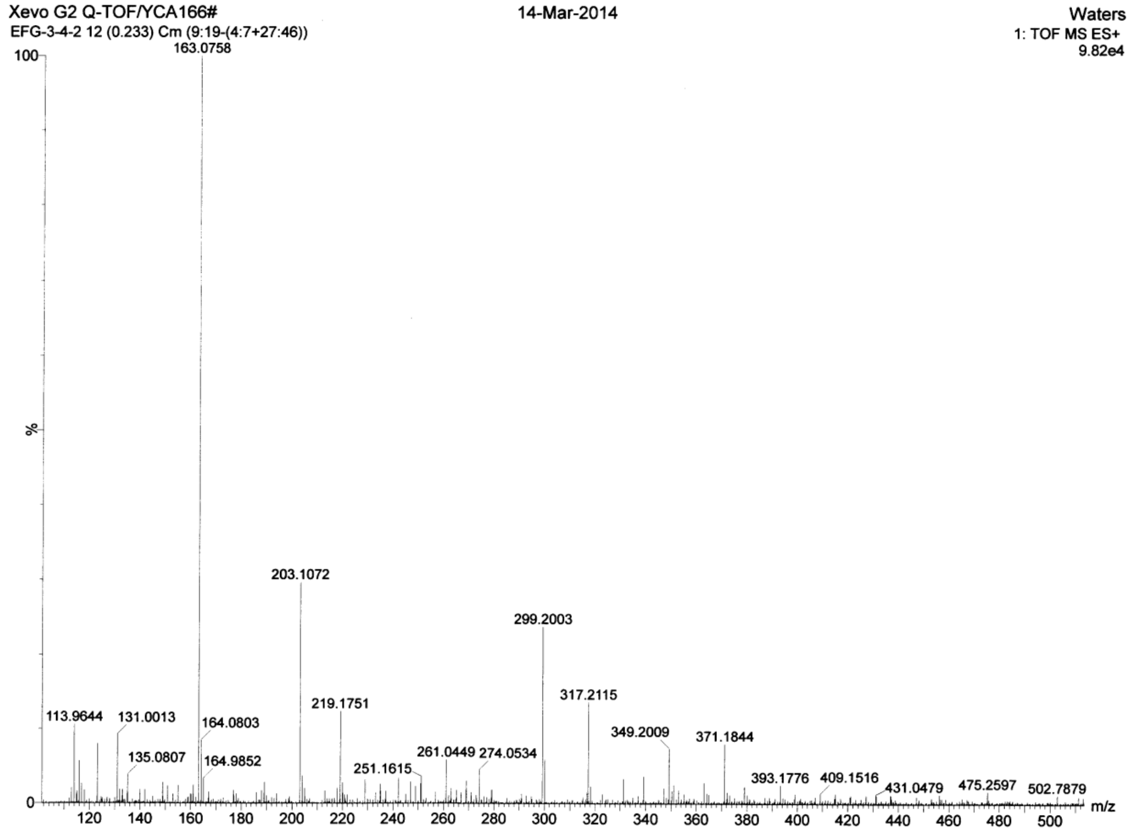

Figure S40. ^{1}H-NMR spectrum of compound **5** in $CDCl_3$.

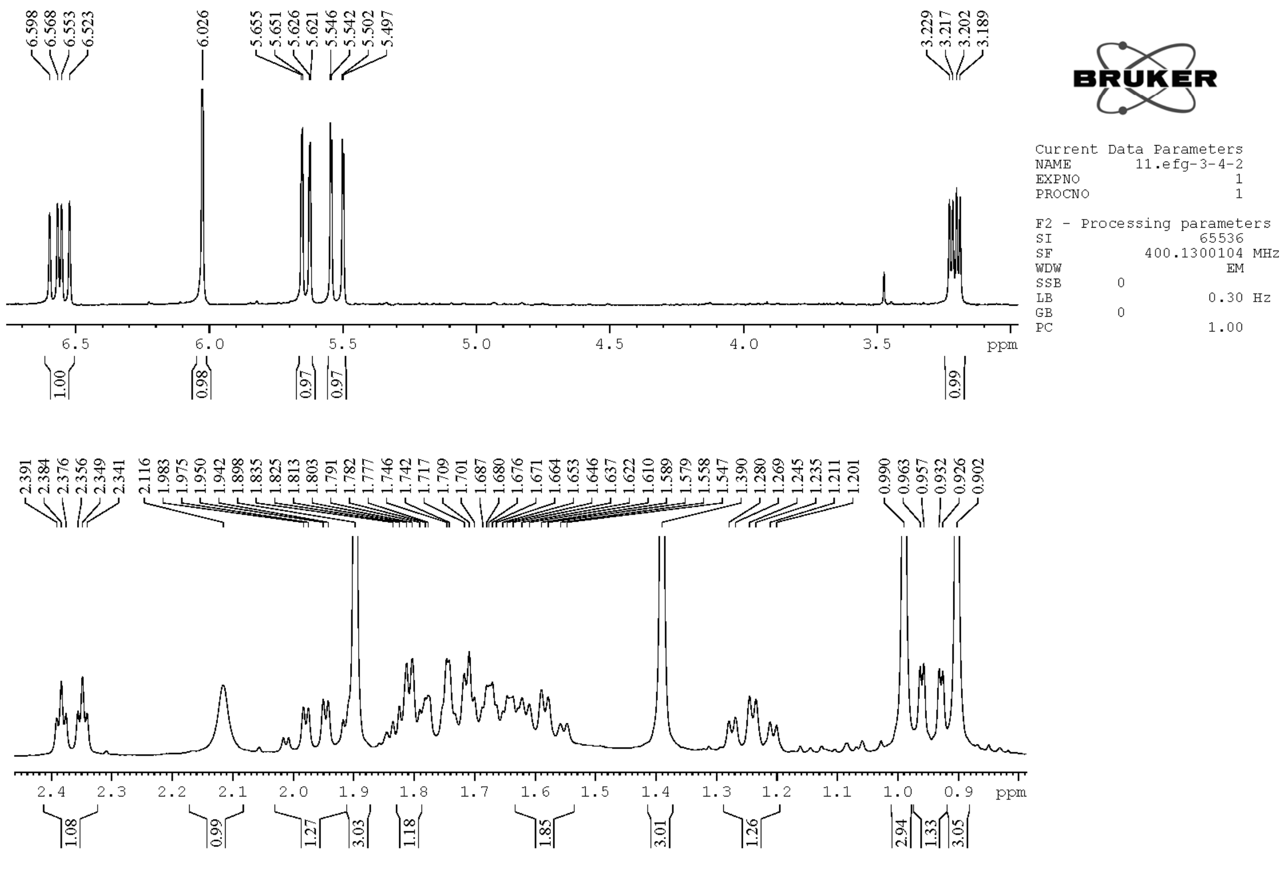

Figure S41. ^{13}C-NMR spectrum of compound **5** in $CDCl_3$.

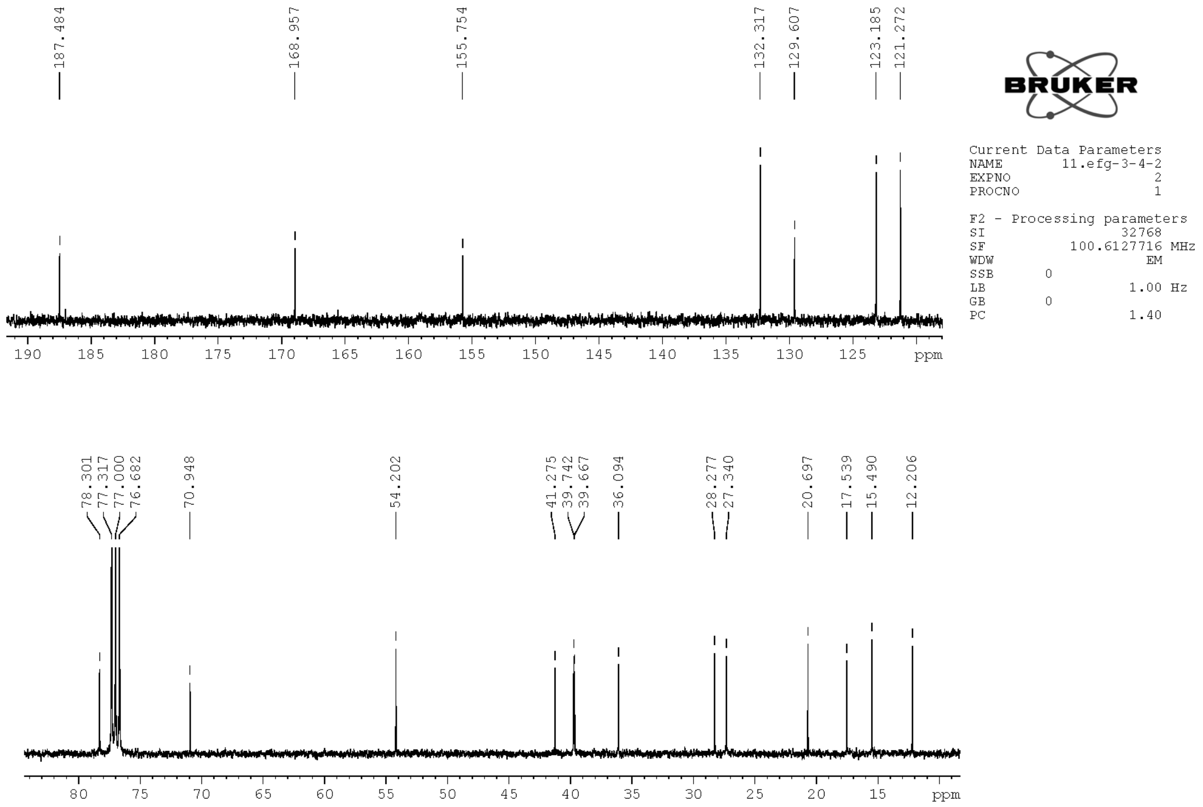

Figure S42. ^{1}H^{1}H COSY spectrum of compound **5** in $CDCl_3$.

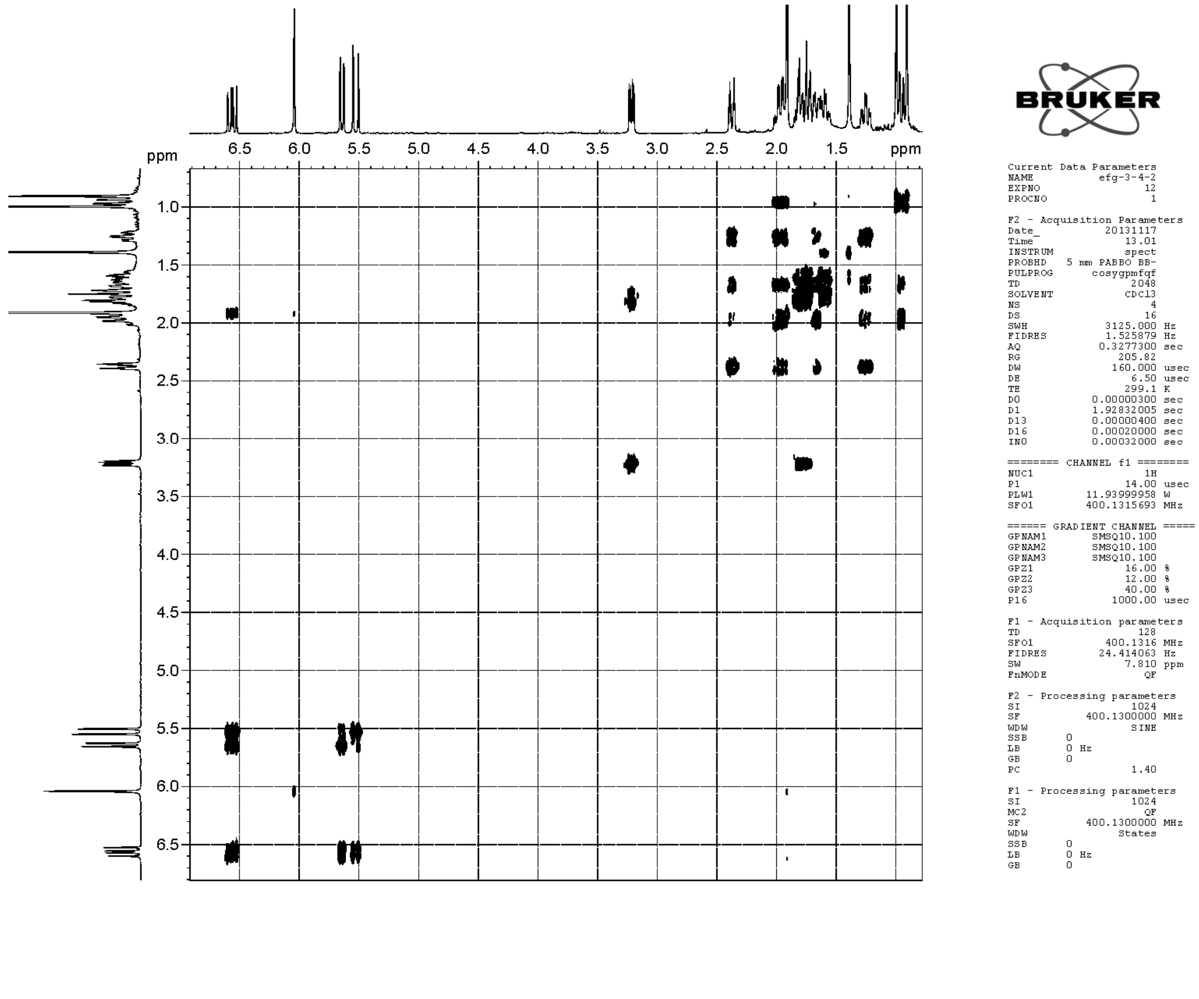

Figure S43. HSQC spectrum of compound **5** in $CDCl_3$.

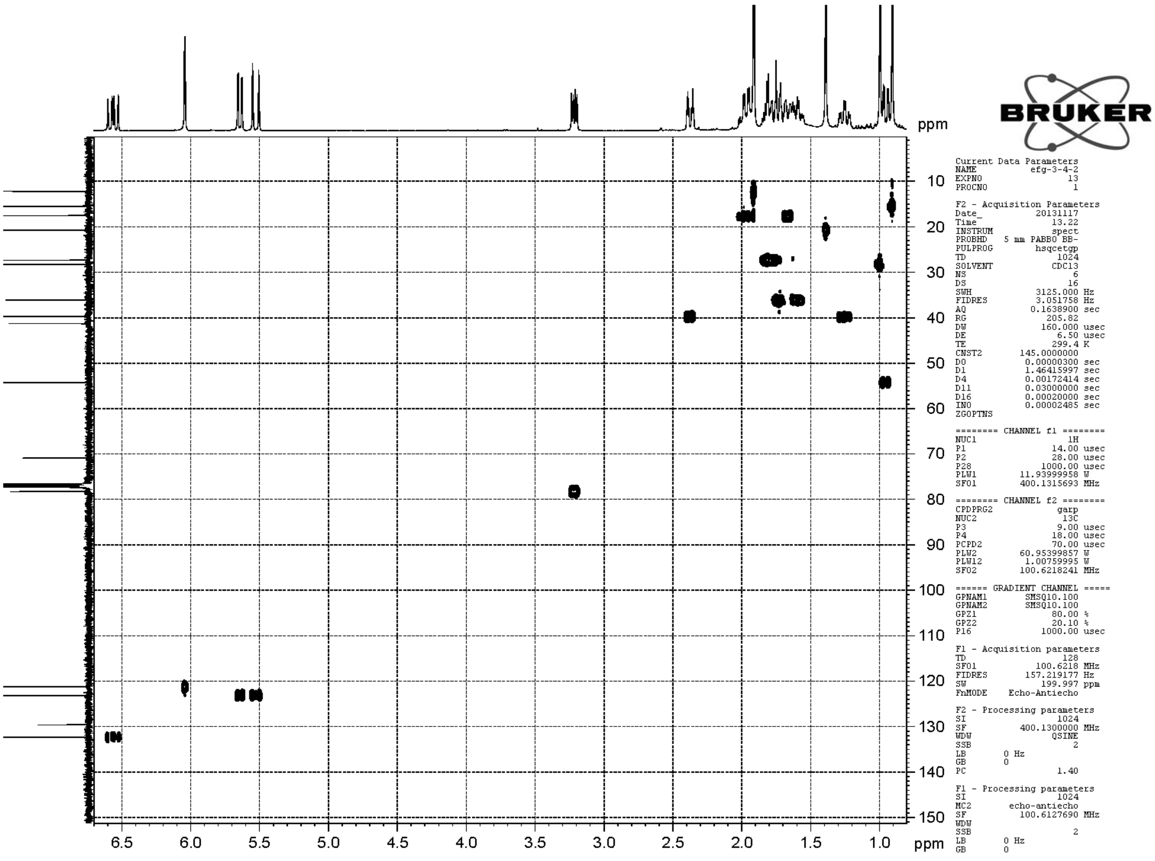

Figure S44. HMBC spectrum of compound **5** in $CDCl_3$.

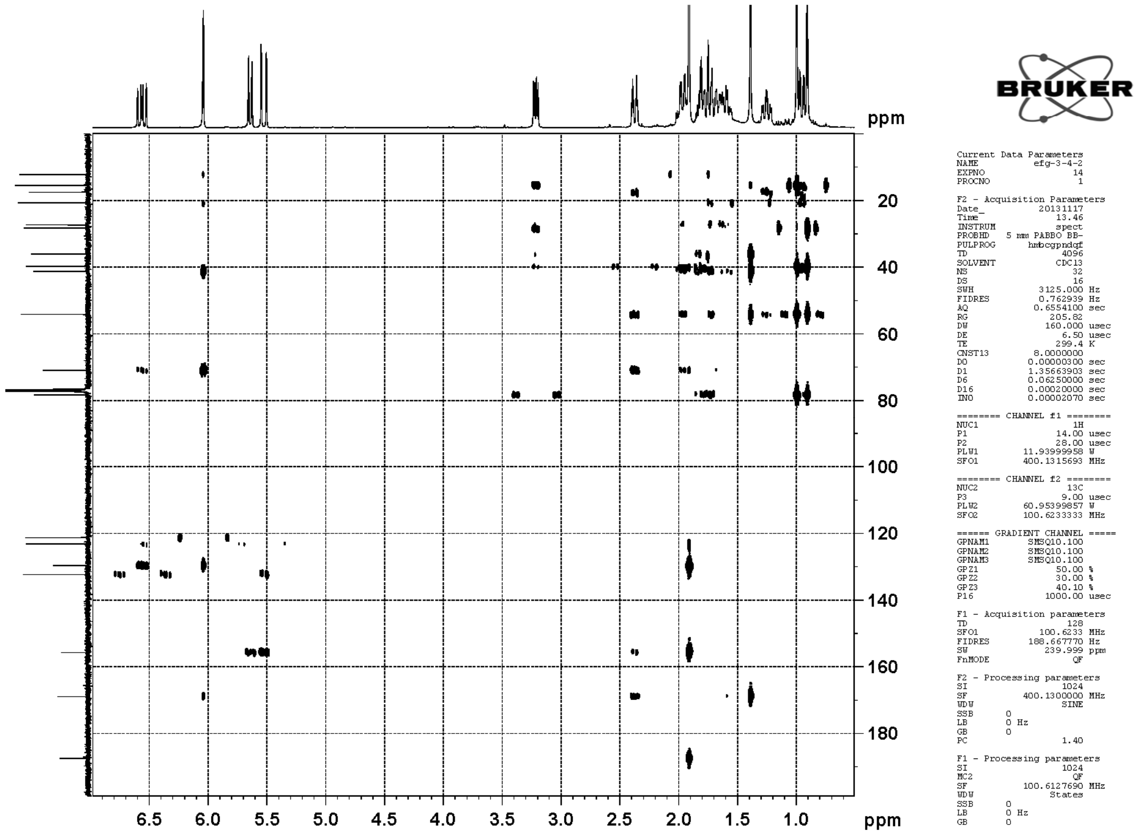

Figure S45. NOESY spectrum of compound **5** in $CDCl_3$.

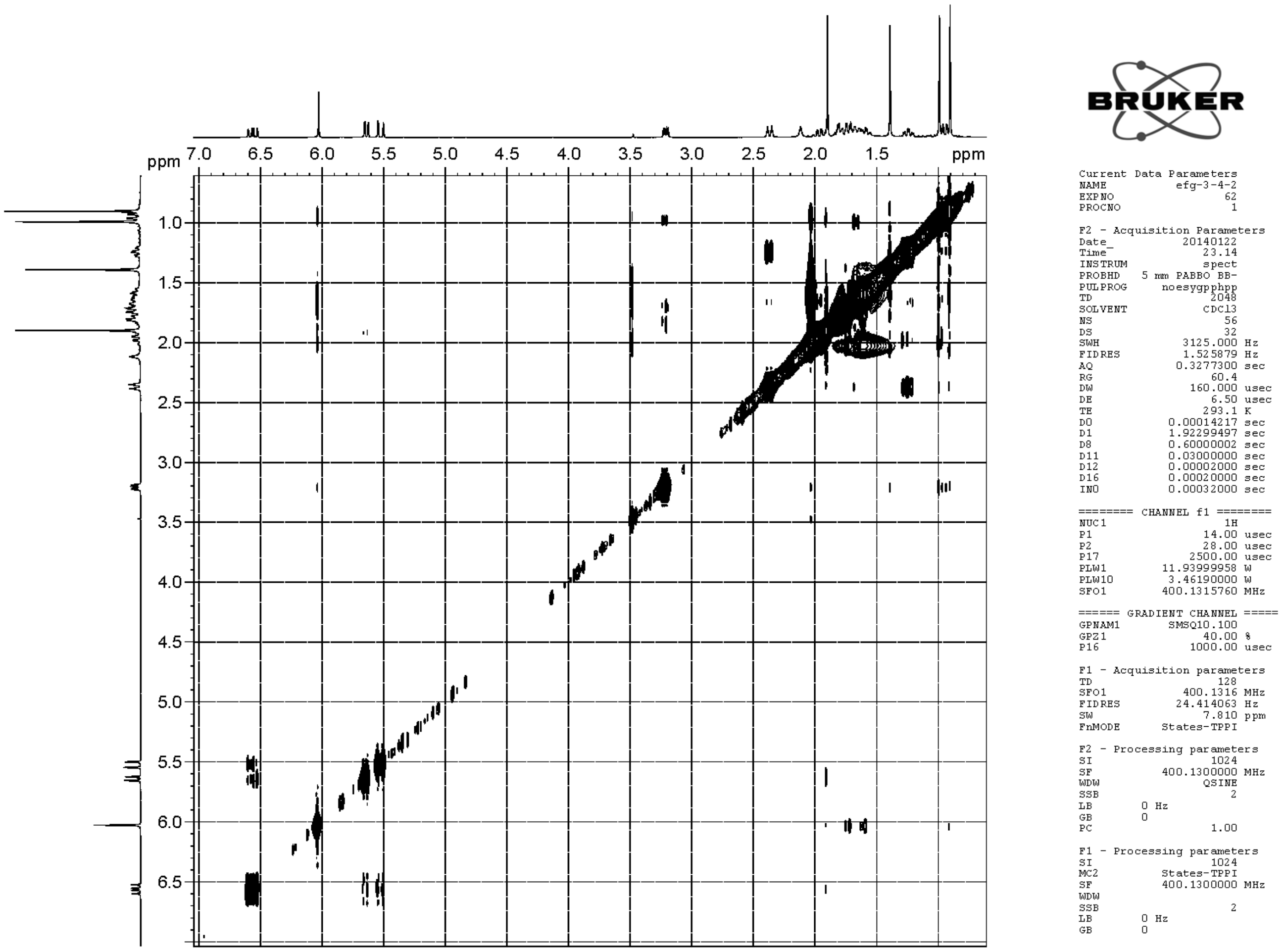

Figure S46. UV spectrum of compound **6** in CH_3OH.

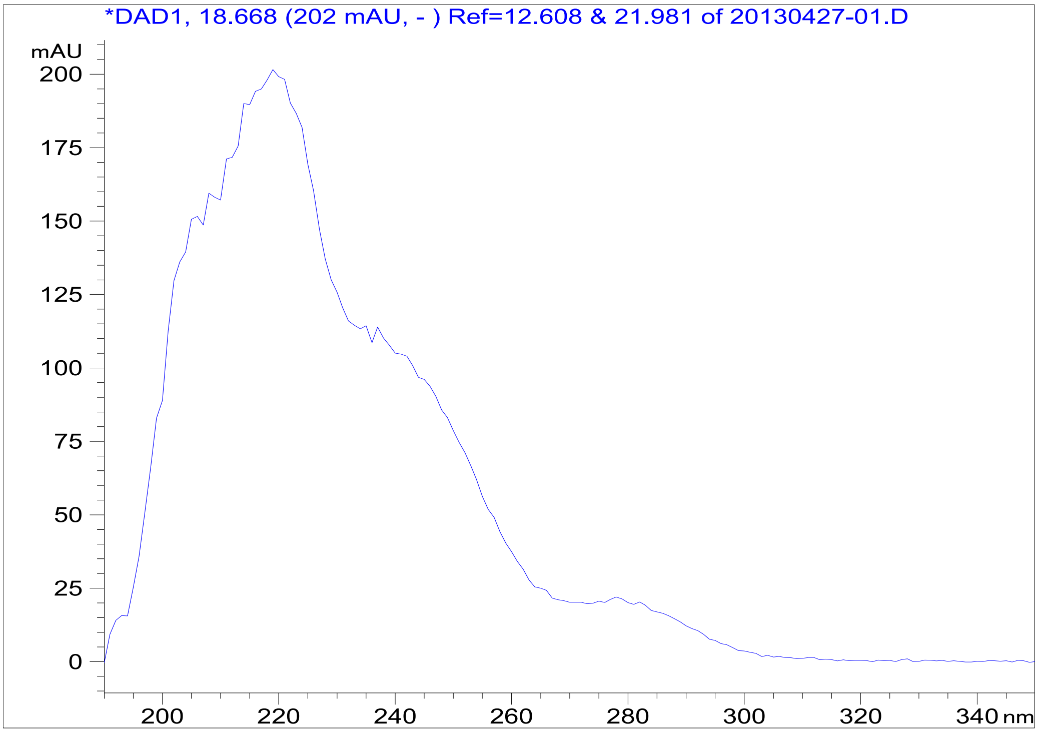

Figure S47. IR spectrum of compound **6**.

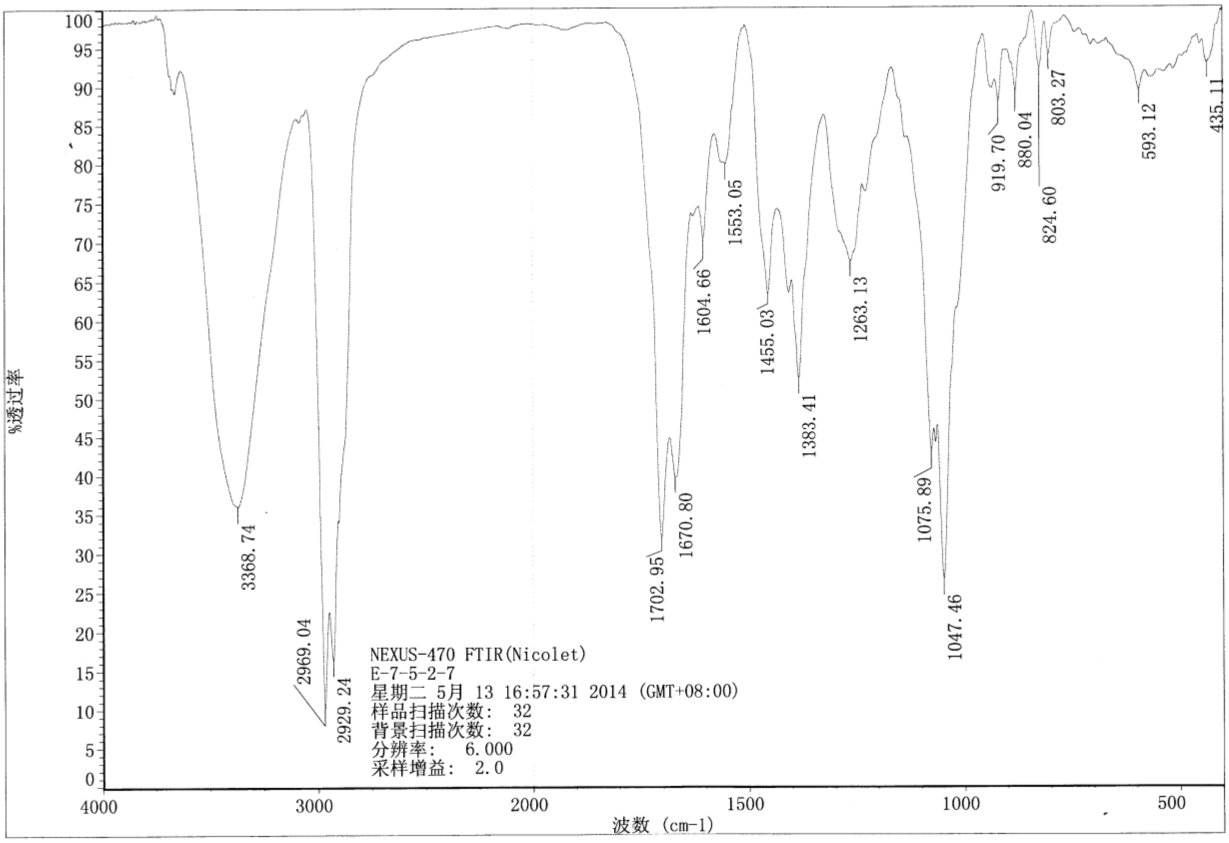

Figure S48. HR-ESI-TOF-MS spectrum of compound **6**.

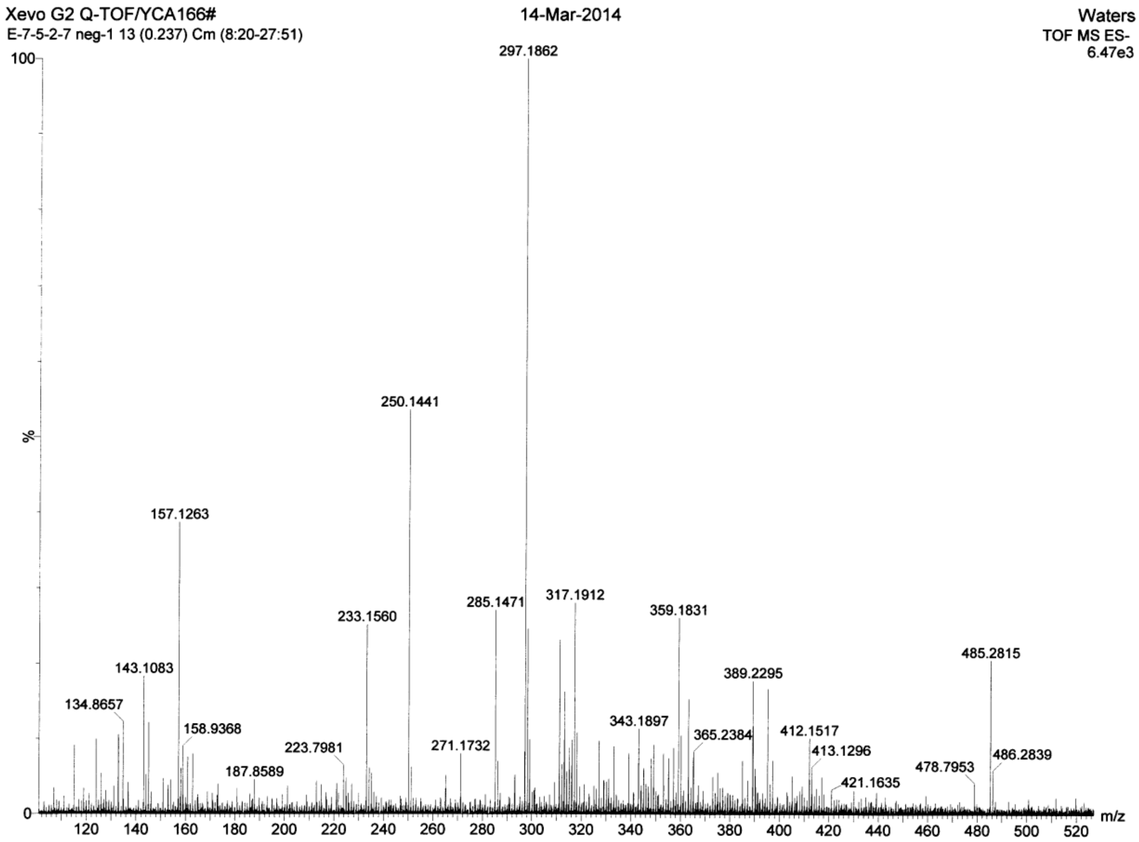

Figure S49. ^{1}H-NMR spectrum of compound **6** in $CDCl_3$.

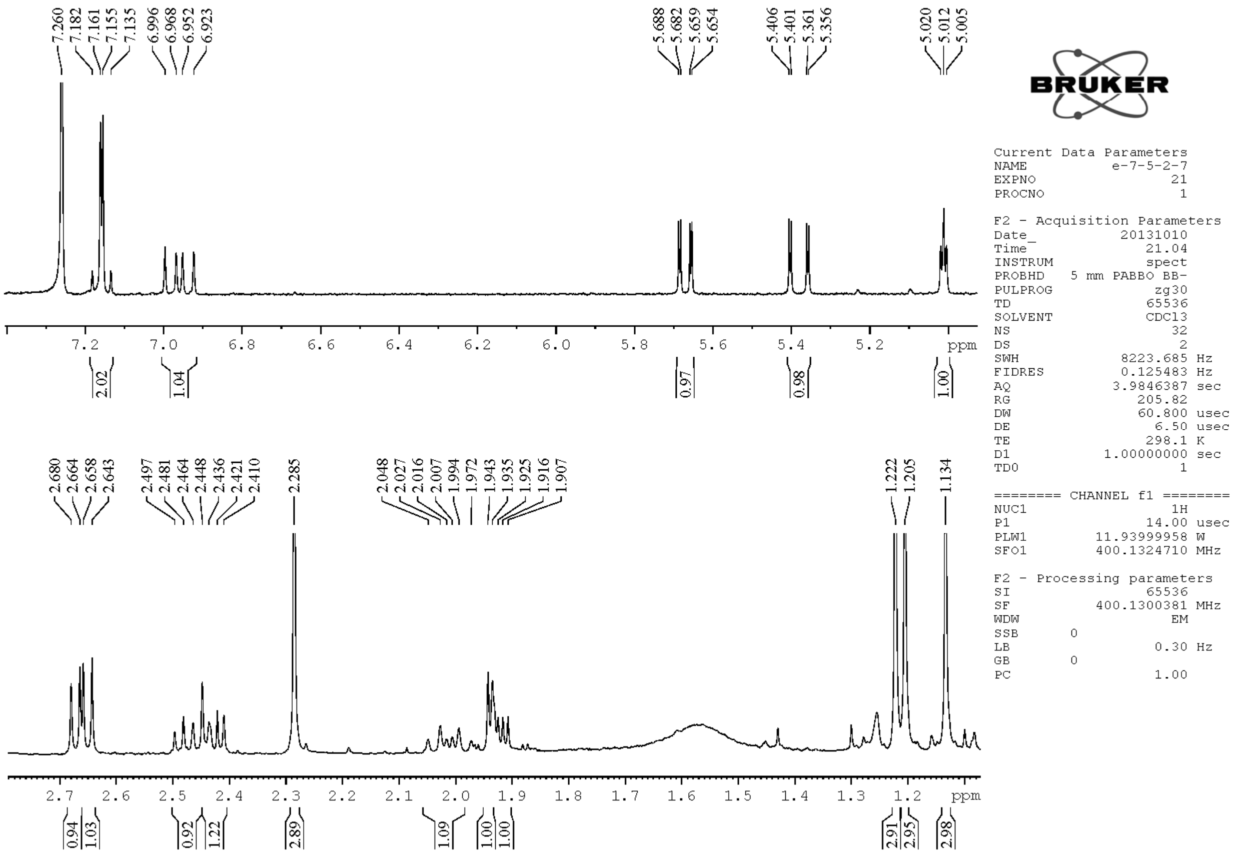

Figure S50. ^{13}C-NMR spectrum of compound **6** in $CDCl_3$.

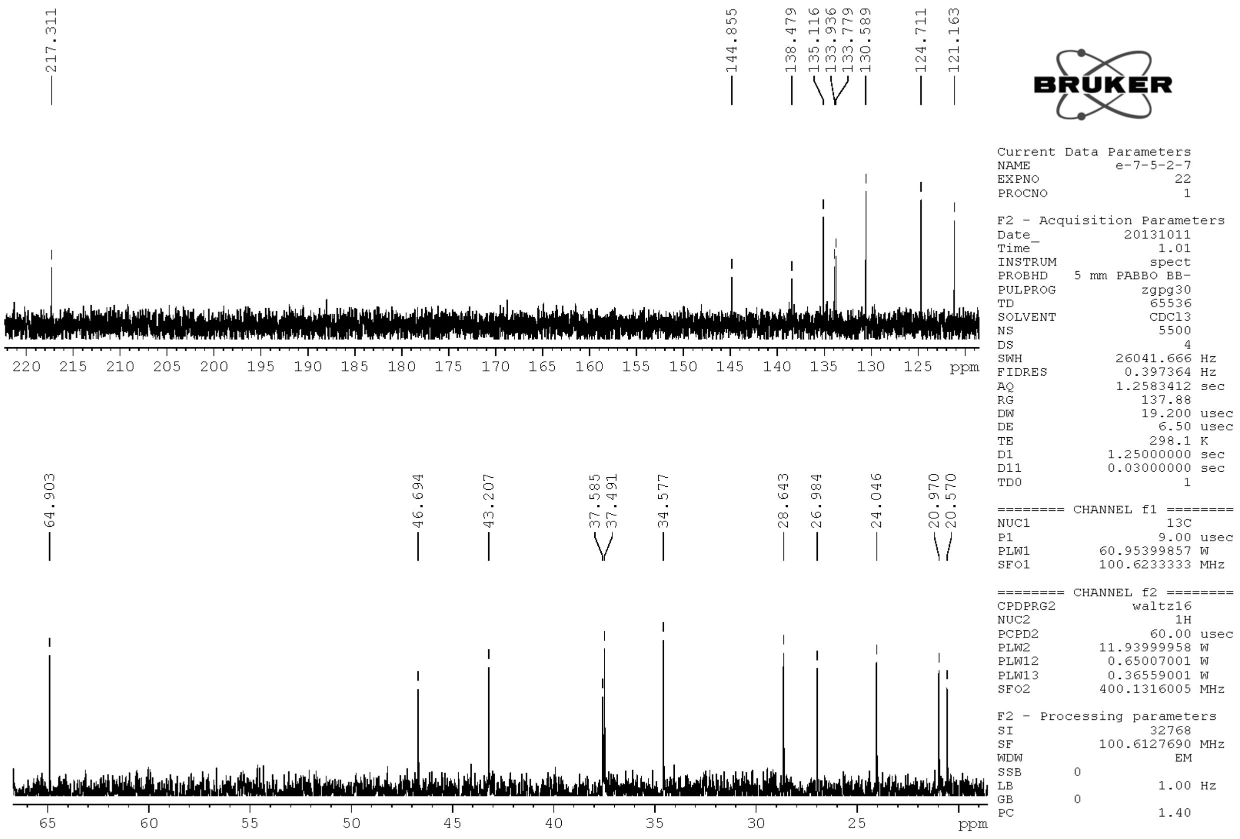

Figure S51. ^{1}H^{1}H COSY spectrum of compound **6** in $CDCl_3$.

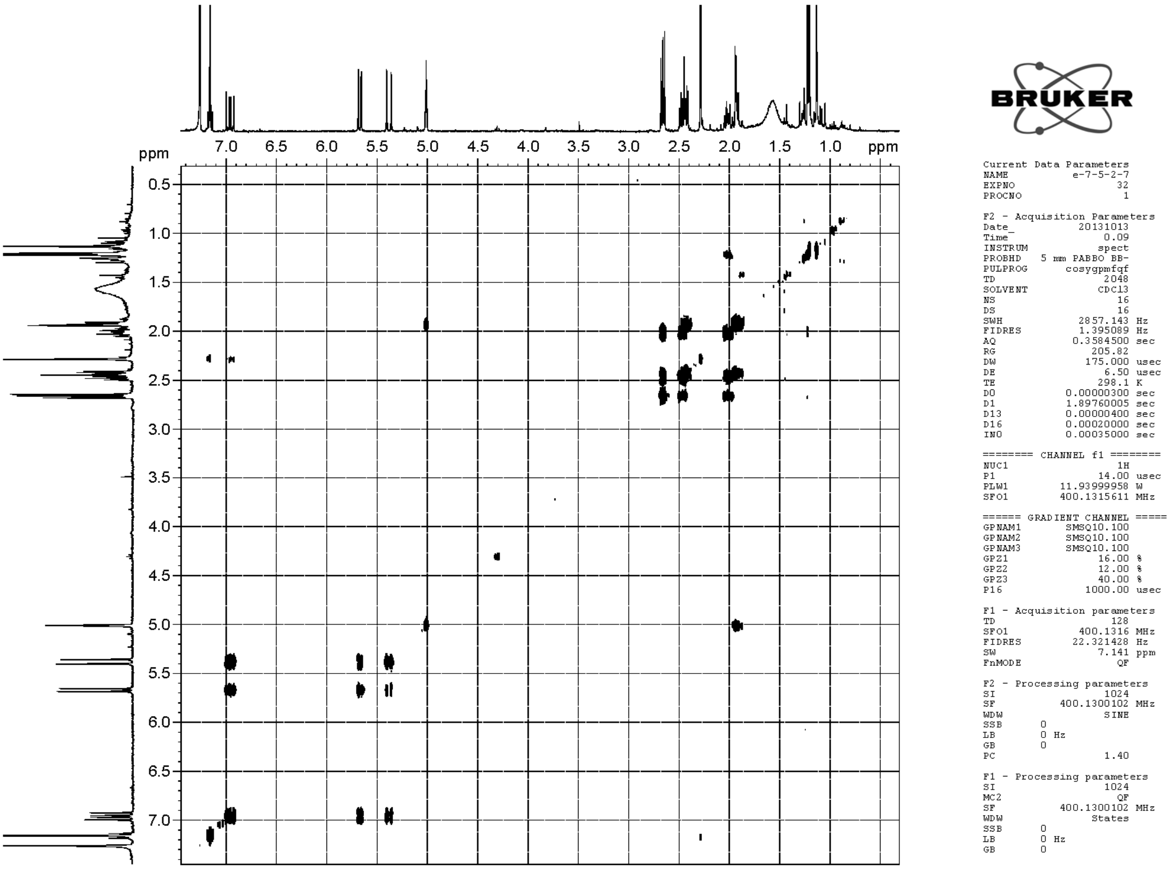

Figure S52. HSQC spectrum of compound **6** in $CDCl_3$.

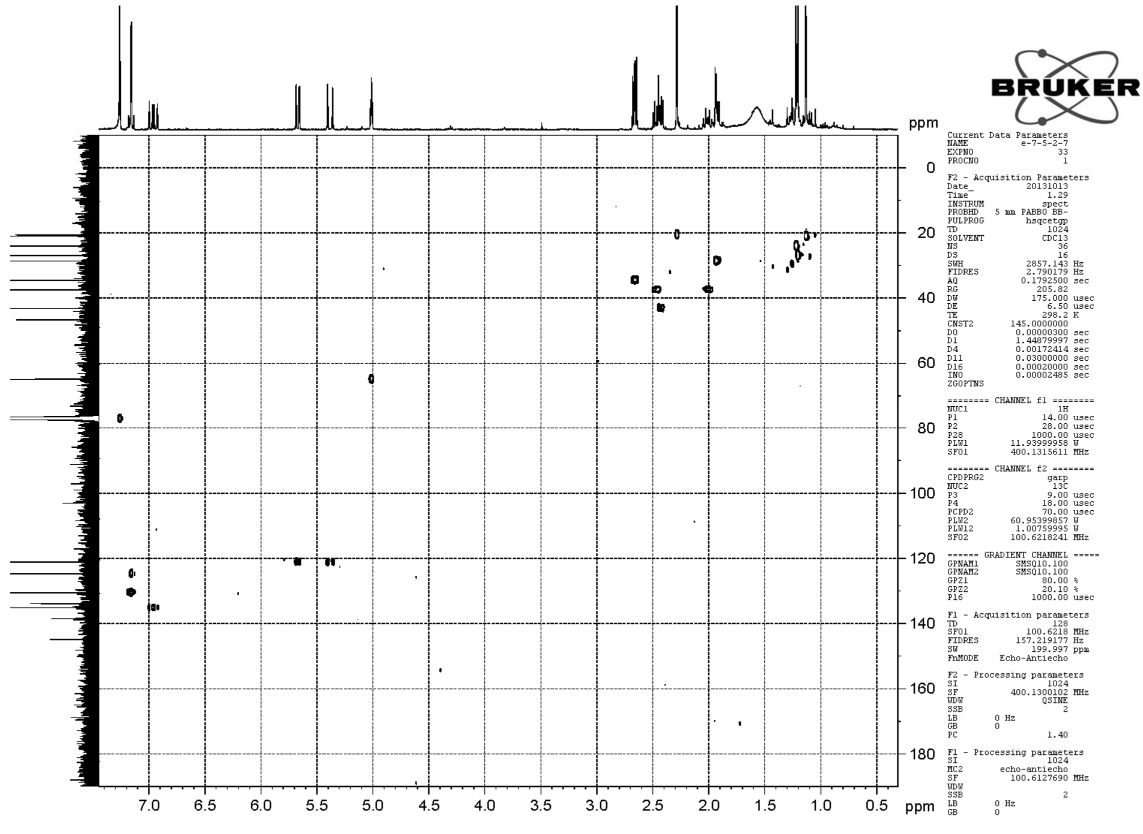

Figure S53. HMBC spectrum of compound **6** in $CDCl_3$.

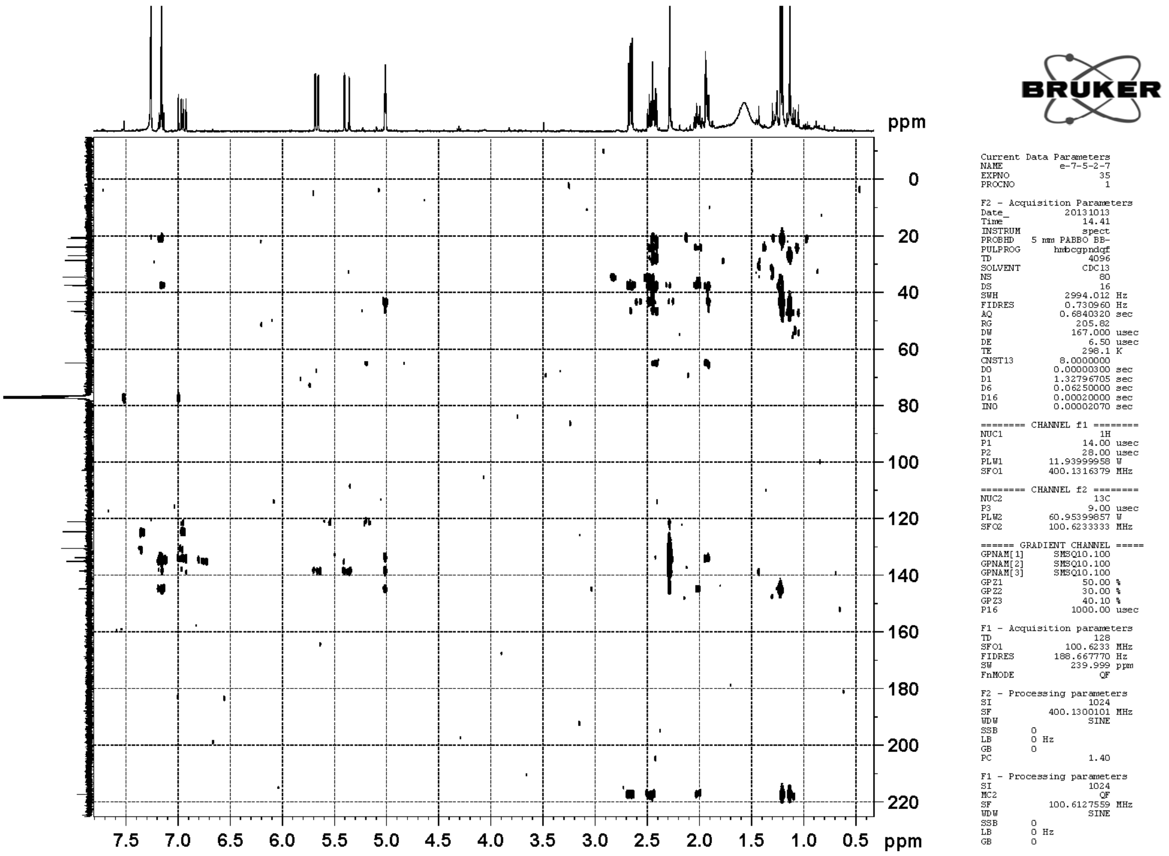

Figure S54. NOESY spectrum of compound **6** in $CDCl_3$.

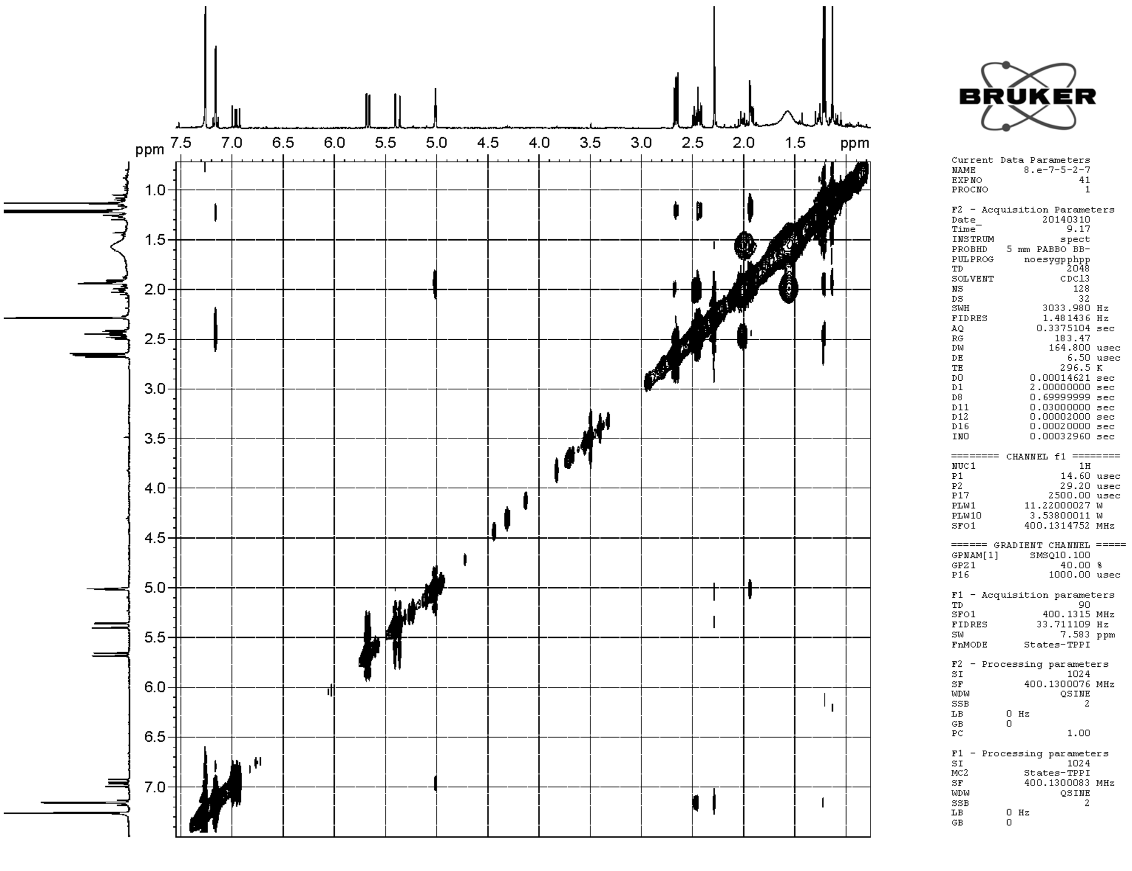

Figure S55. UV spectrum of compound **7** in CH_3OH.

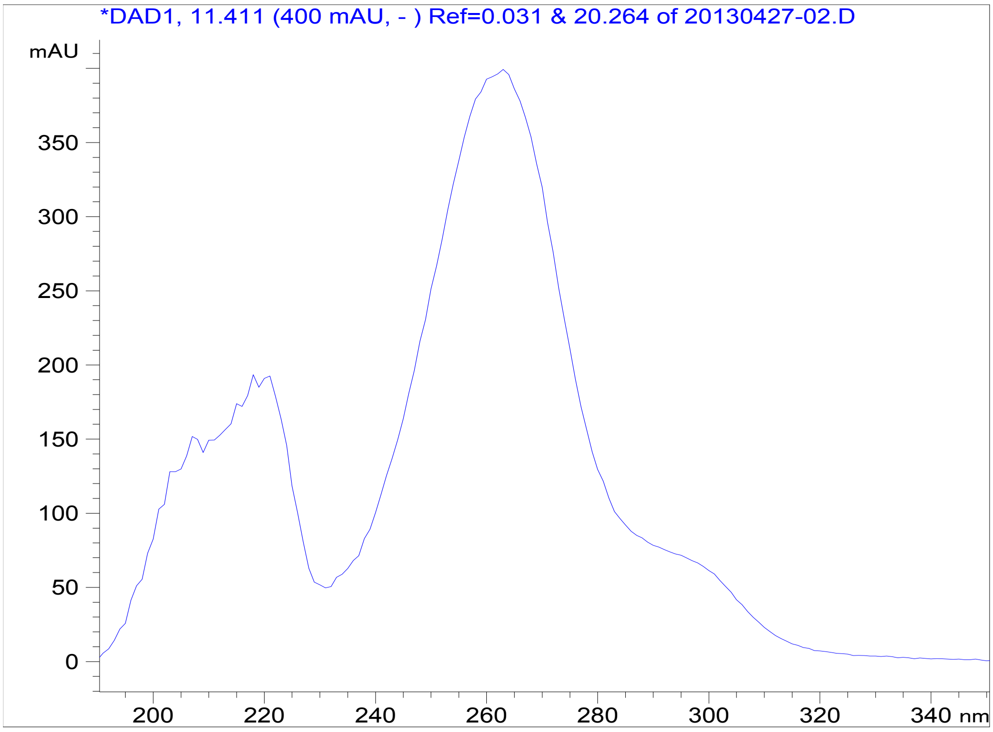

Figure S56. IR spectrum of compound **7**.

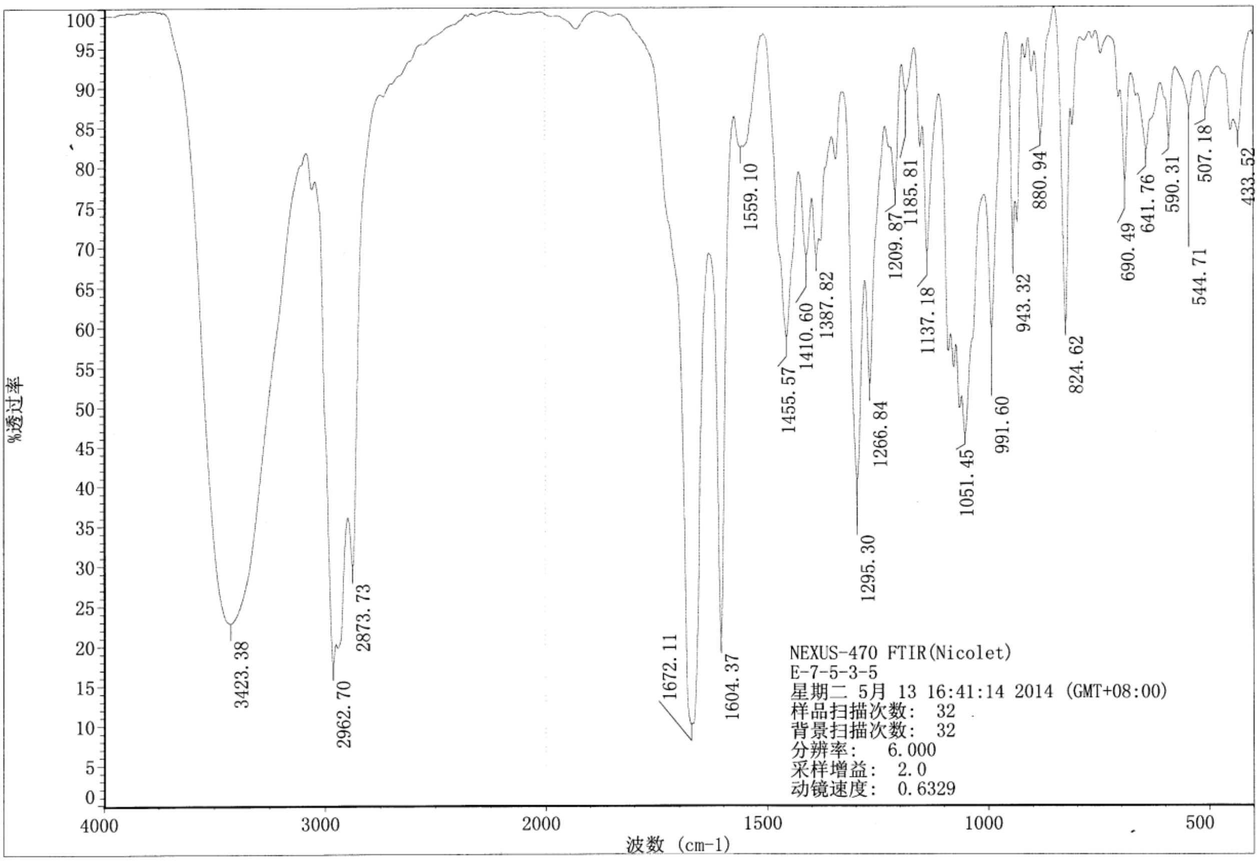

Figure S57. HR-ESI-TOF-MS spectrum of compound **7**.

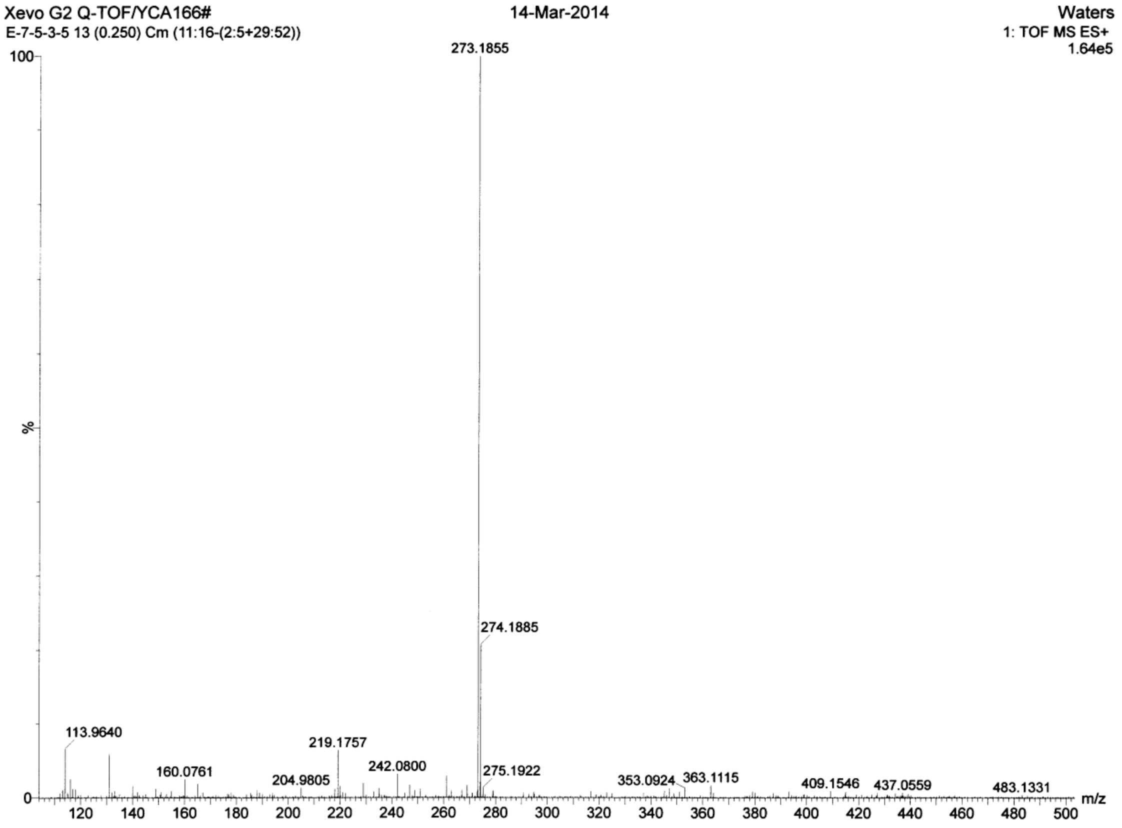

Figure S58. ^{1}H-NMR spectrum of compound **7** in $CDCl_3$.

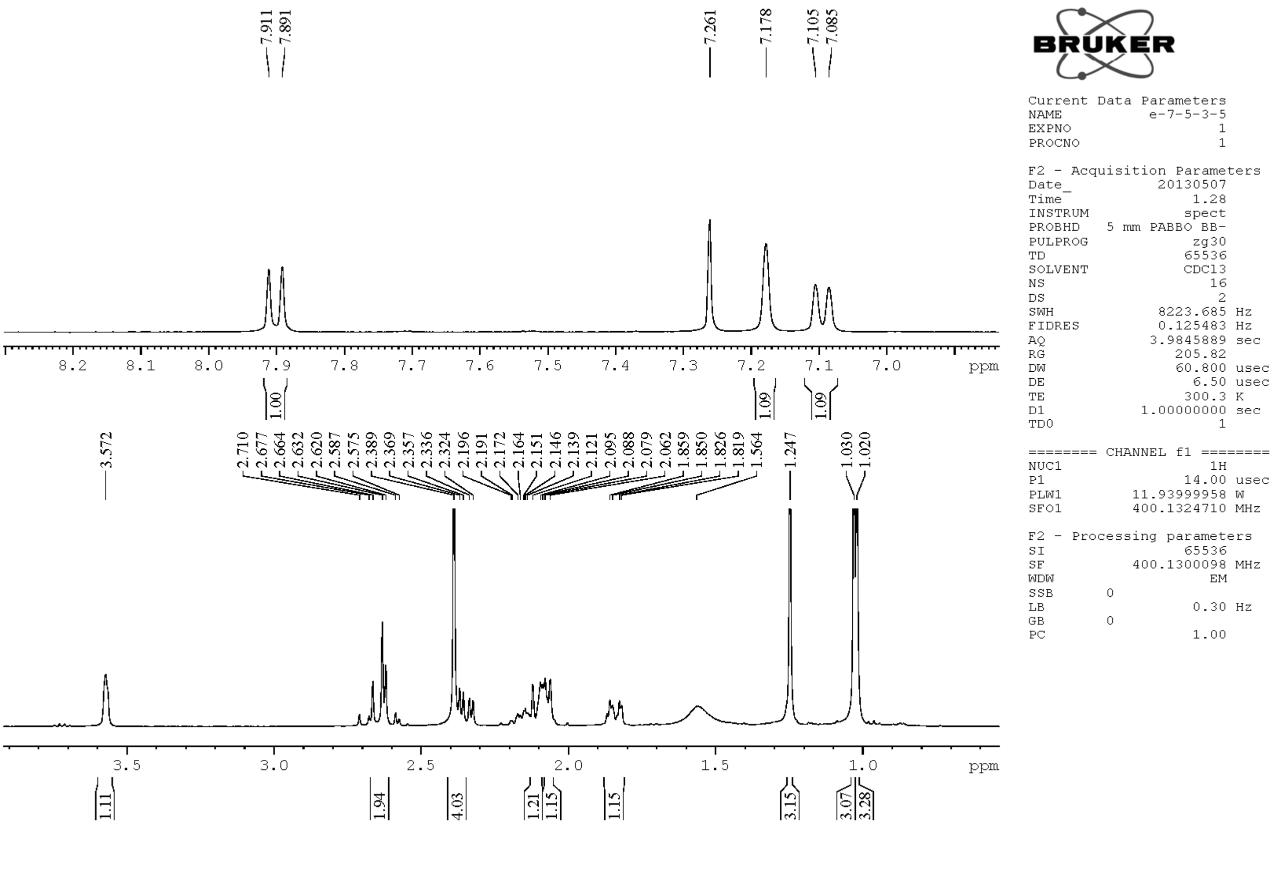

Figure S59. ^{13}C-NMR spectrum of compound **7** in $CDCl_3$.

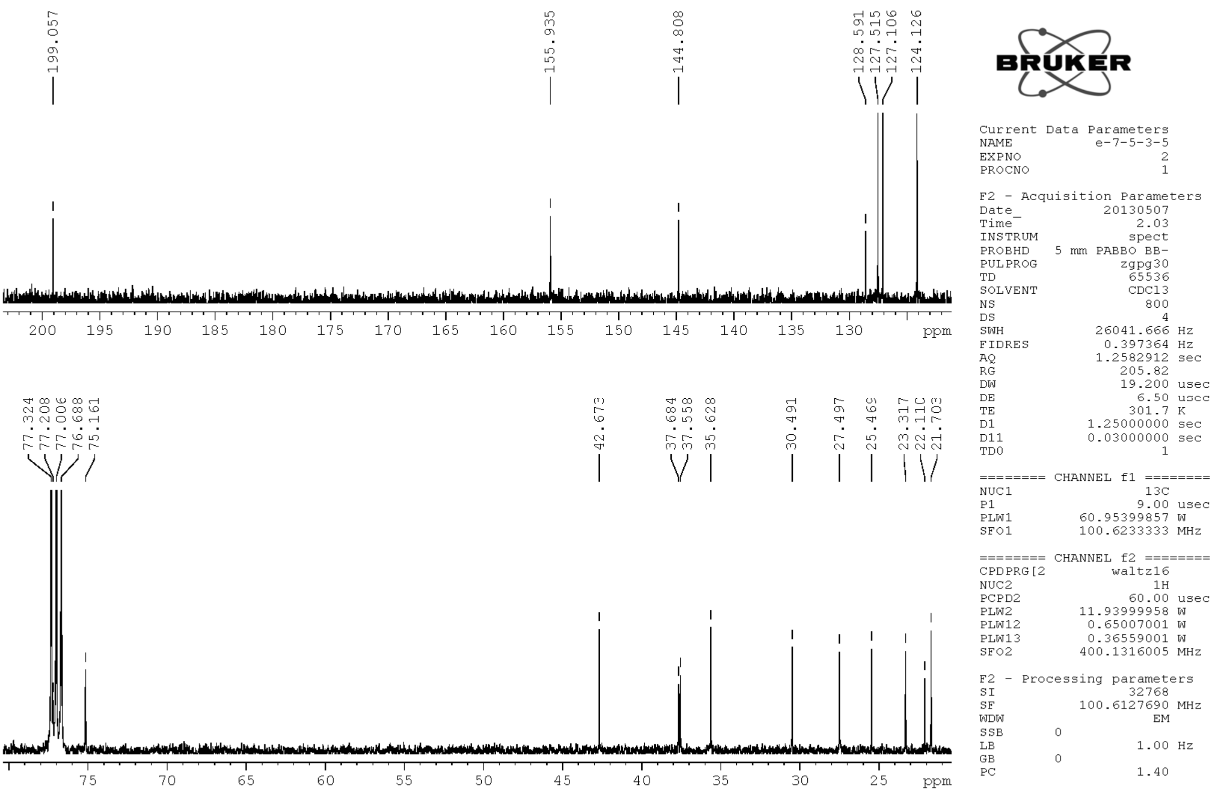

Figure S60. ^{1}H^{1}H COSY spectrum of compound **7** in $CDCl_3$.

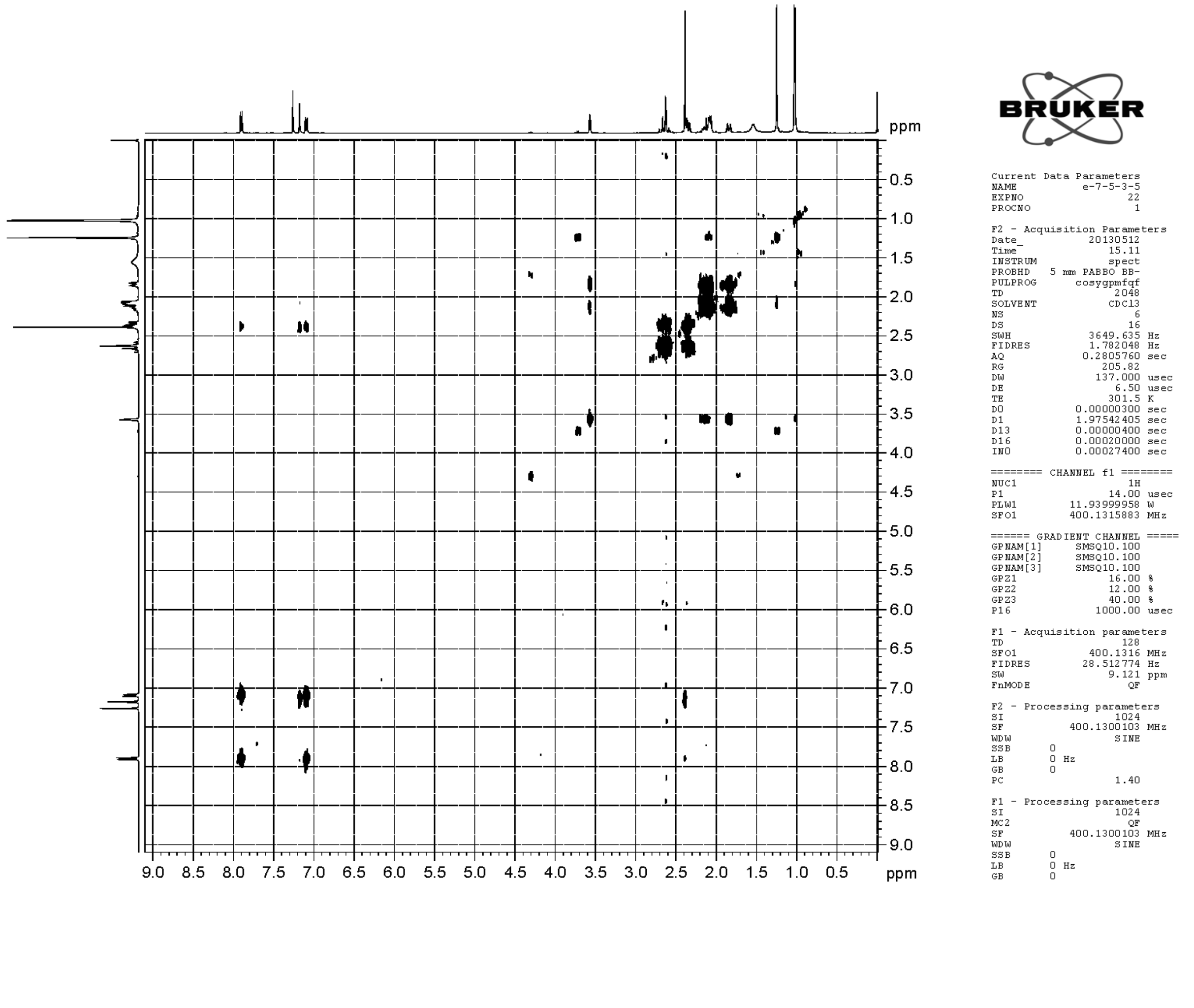

Figure S61. HSQC spectrum of compound **7** in $CDCl_3$.

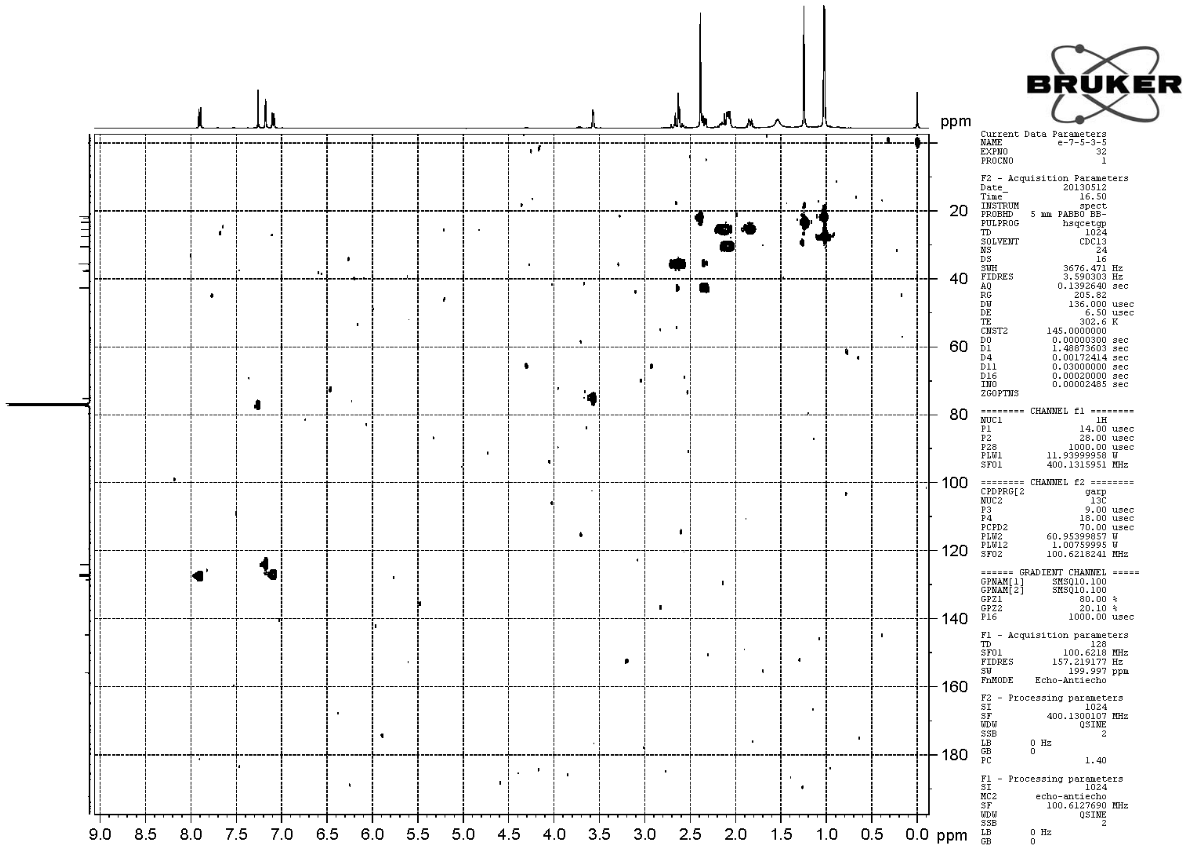

Figure S62. HMBC spectrum of compound **7** in $CDCl_3$.

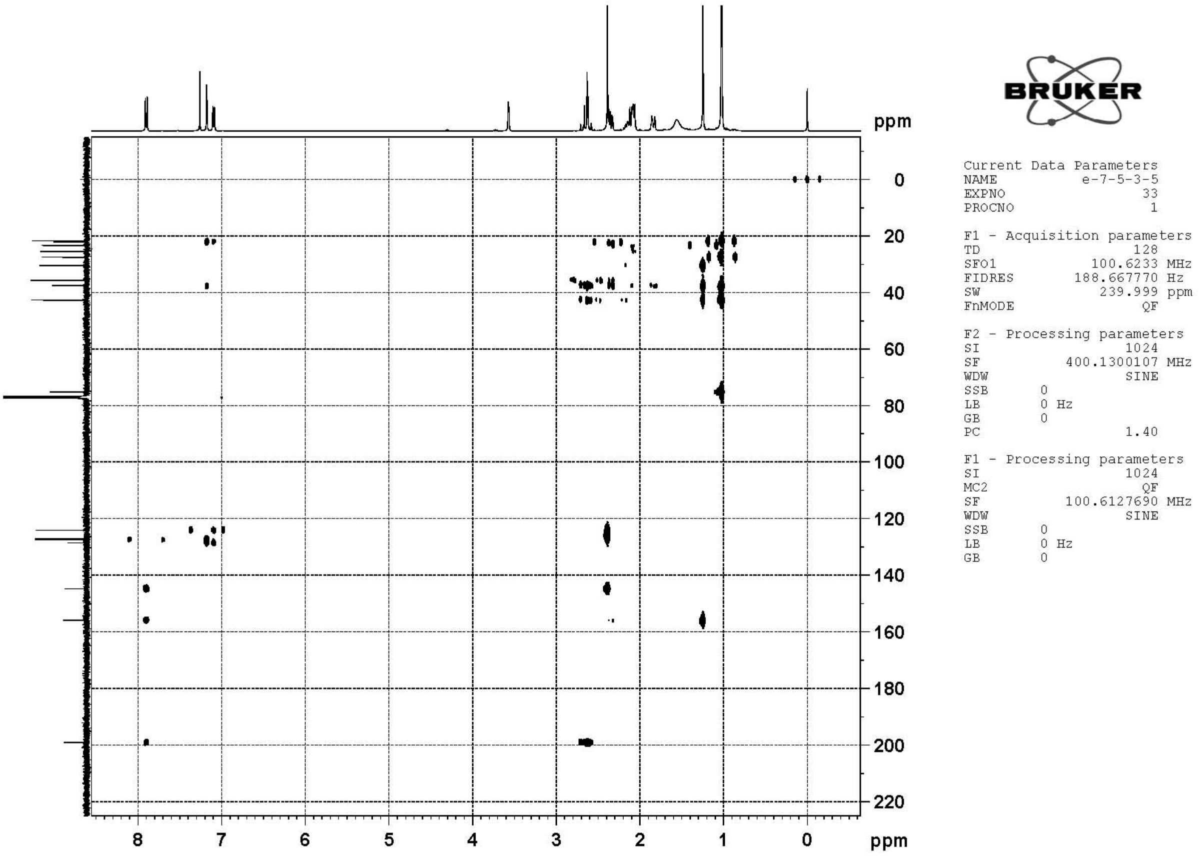

Figure S63. NOESY spectrum of compound **7** in $CDCl_3$.

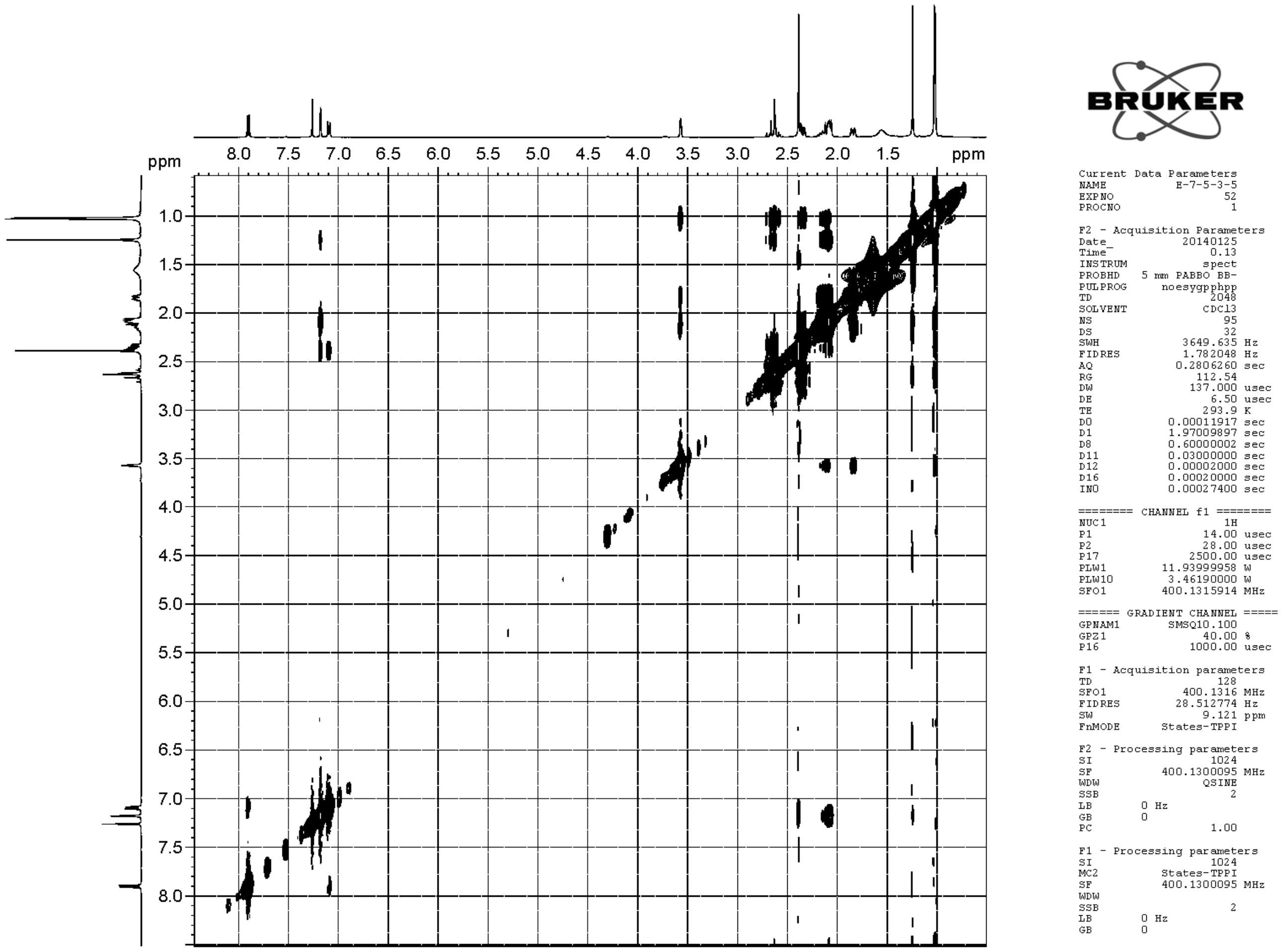

Supplement: Supplementary File 1 [file molecules-19-18966-s001.pdf]
